# Activation of the Aryl Hydrocarbon Receptor Dampens the Severity of Inflammatory Skin Conditions

**DOI:** 10.1016/j.immuni.2014.04.019

**Published:** 2014-06-19

**Authors:** Paola Di Meglio, João H. Duarte, Helena Ahlfors, Nick D.L. Owens, Ying Li, Federica Villanova, Isabella Tosi, Keiji Hirota, Frank O. Nestle, Ulrich Mrowietz, Michael J. Gilchrist, Brigitta Stockinger

**Affiliations:** 1 Division of Molecular Immunology, MRC National Institute for Medical Research, Mill Hill, London NW7 1AA, UK; 2 Division of Systems Biology, MRC National Institute for Medical Research, Mill Hill, London NW7 1AA, UK; 3 St. John’s Institute of Dermatology, King’s College London and NIHR Biomedical Research Centre, London SE1 9RT, UK; 4 Psoriasis Center, Department of Dermatology, University Medical Center Schleswig-Holstein, Campus Kiel, 24105 Kiel, Germany

## Abstract

Environmental stimuli are known to contribute to psoriasis pathogenesis and that of other autoimmune diseases, but the mechanisms are largely unknown. Here we show that the aryl hydrocarbon receptor (AhR), a transcription factor that senses environmental stimuli, modulates pathology in psoriasis. AhR-activating ligands reduced inflammation in the lesional skin of psoriasis patients, whereas AhR antagonists increased inflammation. Similarly, AhR signaling via the endogenous ligand FICZ reduced the inflammatory response in the imiquimod-induced model of skin inflammation and AhR-deficient mice exhibited a substantial exacerbation of the disease, compared to AhR-sufficient controls. Nonhematopoietic cells, in particular keratinocytes, were responsible for this hyperinflammatory response, which involved upregulation of AP-1 family members of transcription factors. Thus, our data suggest a critical role for AhR in the regulation of inflammatory responses and open the possibility for novel therapeutic strategies in chronic inflammatory disorders.

## Highlights

• Physiological AhR signals reduce psoriasis gene expression in patient biopsies
• Blocking AhR signals exacerbates psoriasis gene expression in patient biopsies
• AhR-deficient mice show exacerbated skin inflammation in imiquimod model
• Absence of AhR on mouse or human keratinocytes causes excessive inflammation

## Graphical Abstract

**Figure:**
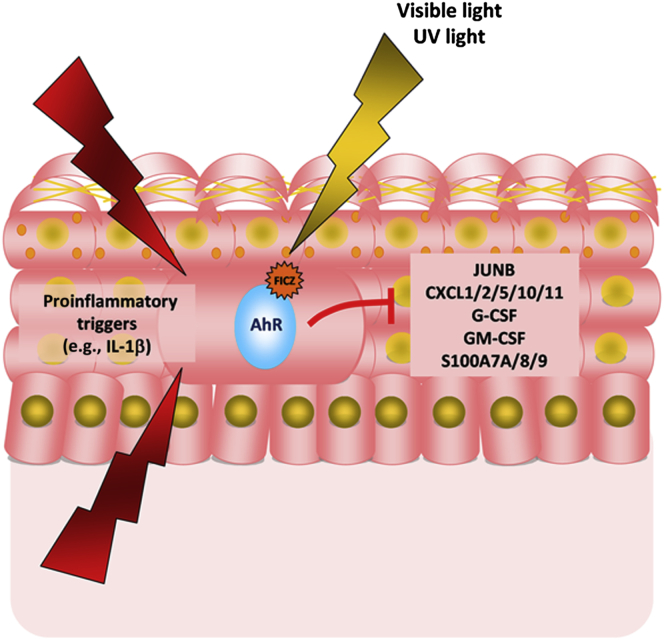

## Teaser

The aryl hydrocarbon receptor (AhR), a transcription factor that responds to environmental signals, interacts with a wide range of genes involved in inflammatory responses. Di Meglio et al. show that physiological stimulation of AhR ameliorates skin inflammation in mice and humans.

## Introduction

The skin is the organ most exposed to environmental insults, and its complex cellular network constitutes an immunological barrier that is crucial for the maintenance of homeostasis (Di Meglio et al., 2011). It is therefore likely that inflammatory disorders of the skin involve environmental factors. One such disorder is plaque-type psoriasis, a disease with complex etiopathogenesis, characterized by epidermal hyperproliferation and prominent immune infiltrates (Nestle et al., 2009). Cross-talk between innate, adaptive, and epithelial or stromal cells, such as keratinocytes and fibroblasts, underpins the disease pathology (Lowes et al., 2013). A total of 36 disease-associated loci have been identified as contributing to psoriasis (Tsoi et al., 2012). Environmental risk factors, on the other hand, remain less well defined on a mechanistic basis (Tagami, 1997). Although no mouse model can fully recapitulate the development and features of psoriasis (Gudjonsson et al., 2007), topical application of the imiquimod (IMQ)-containing cream Aldara induces a psoriasiform skin inflammation, which exhibits most of the crucial traits (Swindell et al., 2011) including acanthosis, parakeratosis, neutrophil recruitment, and involvement of the IL-23-IL-17-IL-22 pathway (van der Fits et al., 2009), and is thus increasingly used to dissect the mechanisms of psoriasis pathogenesis.

In order to investigate the potential influence of environmental factors on inflammatory skin disease, we have focused on the ligand-dependent transcription factor aryl hydrocarbon receptor (AhR), which responds to environmental stimuli and plays an important role in the maintenance of intestinal homeostasis. Work from our lab and others has shown that AhR-deficient mice have an inherent weakness of the gut barrier (Kiss et al., 2011; Lee et al., 2012; Li et al., 2011; Qiu et al., 2012). The wide expression of AhR in several cell types of the skin suggests a role for AhR signaling also at this barrier organ. The AhR is a member of the bHLH-PAS family of transcription factors best known for mediating the toxic effects of environmental contaminants such as TCDD (dioxin) and a range of other xenobiotic substances. However, its evolutionary conservation from invertebrate species onward points to a physiological role that does not involve xenobiotic stimuli (Hahn et al., 2006; McMillan and Bradfield, 2007). Endogenous ligands of AhR are found as indoles and flavonoids either of dietary origin (e.g., indolo[3,2-b]carbazole, ICZ) (Bjeldanes et al., 1991; Gillner et al., 1985) or, like the high-affinity ligand 6-formylindolo[3,2-b]carbazole (FICZ), derived from tryptophan metabolism via UV or visible light exposure (Rannug and Fritsche, 2006), which has been found to be physiologically relevant in human skin (Katiyar et al., 2000; Rannug and Fritsche, 2006).

Combining the analysis of psoriasis patient skin biopsies with that of a mouse model of psoriasiform inflammation, we showed that AhR signaling in nonhematopoietic cells plays a central role in preventing excessive skin inflammation.

## Results

### AhR Ligation in Human Skin Biopsies Modulates Psoriasis-Relevant Genes

**Figure 1:**
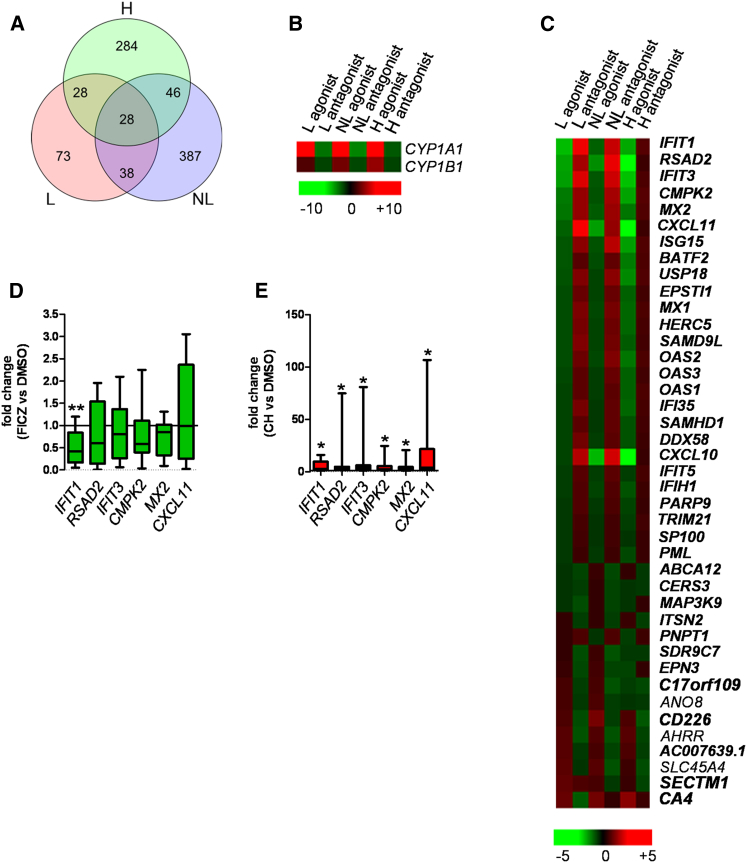
AhR Ligation in Human Skin Biopsies Modulates Psoriasis-Relevant Genes Lesional (L) and nonlesional (NL) skin biopsies from eight psoriasis patients were quartered: one quarter of each was used as baseline and the remaining three quarters were cultured with either vehicle control, the AhR agonist FICZ, or the AhR antagonist CH-223191 for 16 hr. Whole-skin biopsies from five healthy donors (H) were processed in the same way. All samples were subjected to RNA sequencing. (A) Venn diagram showing genes significantly regulated by either FICZ or CH-223191 in at least one out of the three tissue types analyzed (L, NL, H). (B) Heat map of known AhR-target genes modulated by FICZ and CH-223191. Color indicates mean fold change, with green representing decreased and red increased gene expression. (C) Heat map of genes belonging to the “psoriasis transcriptome” (upregulated genes shown in bold) and modulated by FICZ or CH-223191. Genes are sorted by decreasing fold change for agonist effect on L skin. (D) qPCR validation for six top modulated genes downregulated in L skin by agonist-induced AhR activation. Box and whiskers denoting minimum and maximum values are shown. Wilcoxon signed rank test (for *CMPK2*) or paired t test (all other genes) was performed. (E) qPCR validation of six top modulated genes upregulated in NL skin by antagonist-induced AhR inhibition. Box and whiskers denoting minimum and maximum values are shown. Wilcoxon signed rank test was performed.

In order to address whether the AhR pathway has a role in human skin pathology, we investigated whether AhR activation via the agonist FICZ or inhibition via the antagonist CH-223191 (Kim et al., 2006) would cause transcriptional changes in psoriasis-related genes. Full-thickness skin biopsies were obtained from lesional (L) and uninvolved nonlesional (NL) skin of eight psoriatic patients not receiving any systemic treatment and from healthy human subjects (N) serving as control (Table S1 available online). The biopsies were quartered and one segment was reserved for RNA sequencing without any treatment. The remaining three quarters were cultured with DMSO (vehicle control), FICZ, or CH-223191 for 16 hr. After treatment all samples were subjected to RNA sequencing. Analysis of the untreated L and NL psoriasis skin samples provided us with genes differentially regulated in the two tissue types, or the “psoriasis transcriptome.” The top 25 most significantly regulated genes (Table S2) were in keeping with published data sets (Gudjonsson et al., 2010; Tian et al., 2012) with several members of the S100 protein family significantly upregulated in L versus NL skin. Next, we identified the transcriptional changes induced by exposure to AhR agonist or antagonist. A flowchart describing step by step our filtering criteria is shown in Figure S1. We identified 884 AhR-modulated genes, defined as genes significantly regulated by either the agonist or the antagonist in at least one out of the three tissue types analyzed (L, NL, H) (Figure 1A). As expected, expression of *CYP1A* and *CYP1B1*, well-characterized AhR-target genes, were found to be highly upregulated in the presence of agonist and downregulated by the antagonist in all three tissue types (Figure 1B). The list of AhR-modulated genes was reduced to 41 genes belonging to the “psoriasis transcriptome,” which were mainly upregulated in untreated L skin (Figure 1C, psoriasis-upregulated genes are shown in bold, and Table S3). Out of these, 29 (70%) were reduced after FICZ-induced AhR activation. This effect was most prominent in L skin, as confirmed by qRT-PCR showing a downward trend for the top five regulated genes (*IFIT*, *RSAD2*, *IFIT3*, *CMPK2*, *MX2*), which reached statistical significance for IFIT1 (Figure 1D). Conversely, treatment with AhR antagonist was able to increase expression of these genes in NL skin, with statistically significant fold change increase for all genes validated by qRT-PCR (Figure 1E). Ingenuity pathway analysis showed that 26 out of 41 psoriasis-relevant genes modulated by AhR belong to the type I and II IFN pathway, which is known to be upregulated in psoriasis (Figure S1). Thus, AhR appears to play a critical role in modulating the severity of psoriasis. In order to study the influence of AhR in more detail, we employed the mouse model of IMQ-induced psoriasiform inflammation.

### AhR-Deficient Mice Develop Exacerbated IMQ-Induced Psoriasiform Skin Inflammation

**Figure 2:**
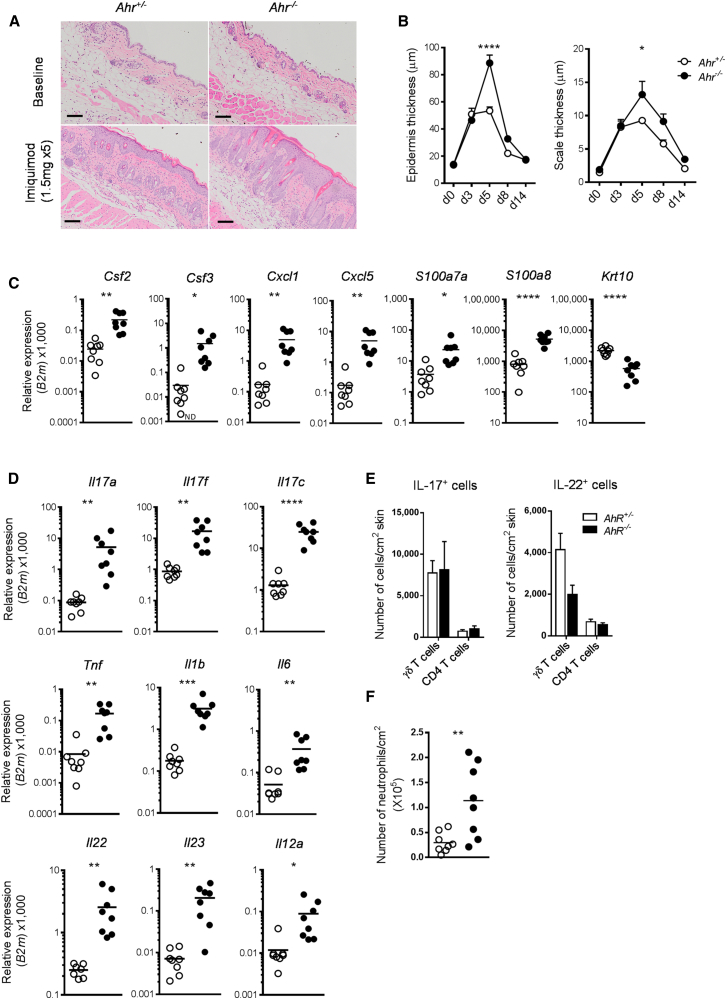
Exacerbated Skin Inflammation in *Ahr*-Deficient Mice (A) Representative images of H&E staining of skin sections from untreated (baseline) and IMQ-treated *Ahr*^+/−^ (open circles) and *Ahr*^−/−^ (filled circles) mice at day 5 (scale bars represent 100 μm). (B) Quantification of epidermal (left) and scale thickness (right) of *Ahr*^*+*/−^ (open circles) and *Ahr*^−/−^ (filled circles) at different time points after initiation of IMQ treatment. (C and D) mRNA expression of proinflammatory mediators and keratinocyte differentiation marker in whole skin from *Ahr*^*+*/−^ (open circles) and *Ahr*^−/−^ (filled circles) mice at day 5. (E) Number of CD4 and γδ T cells expressing IL-17 (left) and IL-22 (right) per cm^2^ skin of IMQ-treated *Ahr*^+/−^ (white bars) and *Ahr*^*−/−*^ (black bars) mice obtained by intracellular cytokine staining. (F) Number of neutrophils (×10^5^) per cm^2^ of skin. Plots show mean ± SEM, n = 3–5 mice per group or mean and values of individual mice, n = 8 mice per group. Results from one representative experiment of two independent experiments are shown. ^∗^p < 0.05, ^∗∗^p < 0.01, ^∗∗∗^p < 0.001, and ^∗∗∗∗^p < 0.0001.

Treatment of AhR-deficient (*Ahr*^−/−^) and AhR-heterozygous littermate control (*Ahr*^+/−^) mice with IMQ over a 5-day period resulted in scaling and parakeratosis of the Stratum corneum and epidermal acanthosis and widespread inflammatory infiltrates, as seen by visual inspection (Figure S2A) and in H&E-stained skin sections (Figure 2A). Untreated skin of *Ahr*^−/−^ and *Ahr*^+/−^ littermate controls was histologically indistinguishable, but upon treatment the thickening of both epidermis and Stratum corneum was significantly increased in *Ahr*^−/−^ mice (Figure 2B). Nevertheless, the exacerbated skin pathology elicited by IMQ treatment in the absence of AhR signaling abated after termination of treatment. Quantitative RT-PCR analysis of inflamed skin from *Ahr*^−/−^ mice revealed statistically significant increased expression of growth factors and chemokines involved in neutrophil attraction (*Csf2*, *Csf3*, *Cxcl1*, *Cxcl5*) and of antimicrobial peptides typically present in psoriasis lesions (*S100a7a*, *S100a8*), as well as reduced expression of the keratinocyte differentiation marker *Krt10* (Figure 2C). Moreover, mRNA expression of a number of proinflammatory cytokines, including *Il17a*, *Il17c*, *Il23*, *Il22*, and *Il1b*, which are crucially involved in psoriatic skin inflammation (Di Cesare et al., 2009; van der Fits et al., 2009), was significantly increased in the skin of *Ahr-*deficient mice (Figure 2D). Upregulation of *Il1b* mRNA in *Ahr*^−/−^ mice preceded that of IL-17 (Figure S2B) and remained increased at protein level on day 5 (Figure S2C). Although there was substantial infiltration of T cells producing IL-17 cell type cytokines, the majority of which were γδ T cells (Figure S2D) as previously reported (Pantelyushin et al., 2012), absolute numbers of IL-17- and IL-22-producing CD4 and γδ T cells did not differ between *Ahr*^+/−^ and *Ahr*^−/−^ mice (Figure 2E). In agreement with our earlier finding (Martin et al., 2009), AhR-deficient γδ T cells appear to make more IL-17 on a per cell basis, thus accounting for the increase in IL-17 observed in the skin of *Ahr*^−/−^ mice (Figures S2E and S2F). In line with the increase in neutrophil-recruiting chemokines, there was significantly more neutrophil infiltration into the skin in *Ahr*^−/−^ mice (Figure 2F), whereas the number of both macrophages and dendritic cells (DCs) did not differ between the two groups (Figure S2G), and we did not observe substantial infiltration of IL-17-producing CD8 T cells or innate lymphoid cells (data not shown). Thus, absence of AhR signaling led to heightened inflammation and exacerbated skin pathology. This phenotype was not restricted to the psoriasisform inflammation induced by IMQ, but also extended to a model of delayed-type hypersensitivity (DTH) skin reactions in *Ahr*-deficient mice, which showed enhanced neutrophil infiltration and increased inflammatory chemokine expression (data not shown).

### AhR Activation by FICZ Ameliorates IMQ-Induced Psoriasiform Skin Inflammation

**Figure 3:**
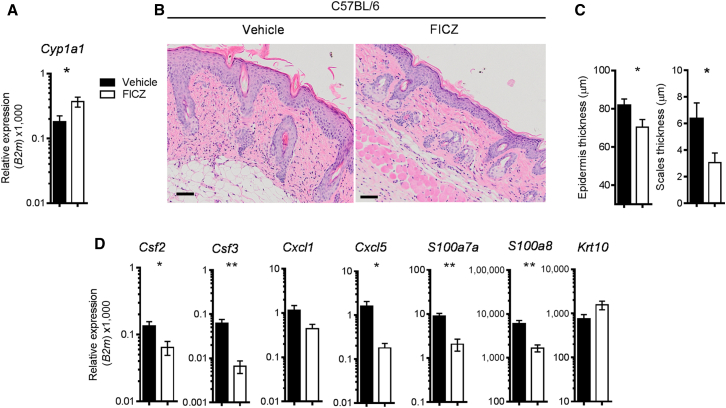
Skin AhR Activation by FICZ Ameliorates IMQ-Induced Psoriasis-like Skin Inflammation (A) *Cyp1a1* expression in skin of IMQ-treated C57BL/6 mice receiving vehicle (black bars) or FICZ (white bars) i.p. for 6 days. (B) H&E staining of skin sections (day 6) from IMQ-treated C57BL/6 mice receiving vehicle or FICZ. (C) Quantification of epidermal (left) and scale thickness (right) at day 6 of vehicle-treated (black bars) or FICZ-treated (white bars) mice. (D) mRNA expression of psoriasis-relevant genes in whole skin from IMQ-treated C57BL/6 mice receiving vehicle (black bars) or FICZ (white bars). Plots show mean ± SEM; n = 5 mice per group. Results from one representative experiment of three independent experiments are shown. ^∗^p < 0.05, ^∗∗^p < 0.01.

Next, we asked whether deliberate triggering of the AhR pathway would have a beneficial effect on skin pathology as seen in human psoriatic skin. To this end, wild-type mice received intraperitoneal injections of either FICZ or olive oil (vehicle control) daily during the course of IMQ treatment. FICZ administration upregulated expression of *Cyp1a1* mRNA in the skin, as compared to mice receiving vehicle only (Figure 3A), and resulted in attenuated psoriasiform skin inflammation, with milder parakeratosis and cell infiltration (Figure 3B), statistically significant reduction in epidermal and scale thickness (Figure 3C), and reduced expression of proinflammatory mediators (Figures 3D and S3). Thus, activation of the AhR pathway in vivo results in amelioration of psoriasiform skin pathology.

### AhR Deficiency in Nonhematopoietic Cells Causes Exacerbated Skin Inflammation

**Figure 4:**
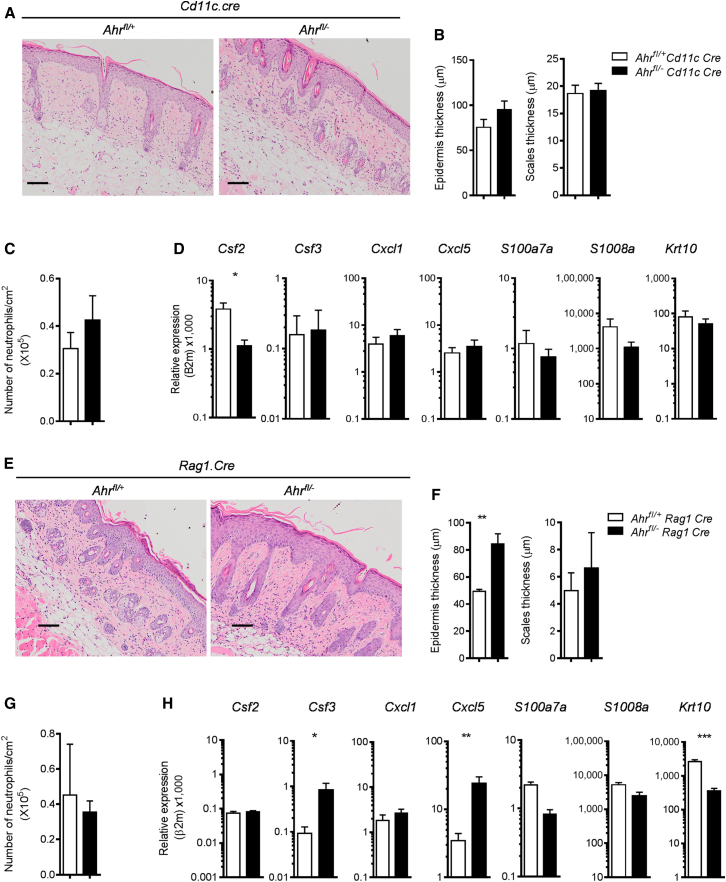
AhR Deficiency in Dendritic Cells or in T and B Cells Does Not Recapitulate the Phenotype of *Ahr*^−/−^ Mice (A) Representative images of H&E staining of skin sections from imiquimod-treated *Ahr*^fl/+^ or *Ahr*^fl/−^*Cd11c.Cre* mice at day 5 (scale bars represent 100 μm). (B) Quantification of epidermal (left) and scale thickness (right) at day 5. (C) Number of neutrophils per cm^2^ of skin as determined by FACS analysis of Ly6G^+^ cells. (D) mRNA expression of psoriasis-relevant genes in whole skin from *Ahr*^fl/+^ (white bars) or *Ahr*^fl/−^*Cd11c.Cre* mice (black bars) at day 5. (E) Representative images of H&E staining of skin sections from imiquimod-treated *Ahr*^fl/+^ or *Ahr*^fl/−^*Rag1.Cre* mice (scale bars represent 100 μm). (F) Quantification of epidermal (left) and scale thickness (right) at day 5. (G) Number of neutrophils per cm^2^ of skin as determined by FACS analysis of Ly6G^+^ cells. (H) mRNA expression of psoriasis -relevant genes in whole skin from *Ahr*^fl/+^ (white bars) or *Ahr*^fl/−^*Rag1.Cre* mice (black bars) at day 5. Plots show mean + SEM; n = 4–6 mice per group. Results from one representative experiment of two independent experiments per mouse strain are shown. ^∗^p < 0.05, ^∗∗^p < 0.01, and ^∗∗∗^p < 0.001.

In order to identify the AhR-expressing cell type responsible for the hyperinflammatory skin response seen in *Ahr*^*−*/−^ mice, we generated mice with conditional deletion of AhR in distinct immune cell subsets in the skin. *Ahr*^fl/−^ *Cd11c*.Cre or *Ahr*^fl/−^ *Rag1*.Cre mice, in which AhR is deleted in DCs and some macrophage subsets or in T and B cells, respectively, were treated with IMQ in order to address the contribution of these cells to the exacerbated skin inflammation seen in complete AhR deficiency. qPCR analysis confirmed the deletion of AhR in DCs or in T cells of these mice (Figures S4A and S4C). Lack of AhR in DCs or macrophages did not result in increased inflammation above that observed in control *Ahr*^fl/+^ *Cd11c*.Cre mice (Figures 4A–4D and S4B). Lack of AhR in T and B cells resulted in increased skin acanthosis and reduced keratinocyte differentiation, but no difference in skin scaling, expression of the majority of inflammatory mediators, or number of neutrophils when compared to control mice (Figures 4E–4H and S4D). These observations ruled out a role for DCs or macrophages in driving the exacerbated skin inflammation seen in *Ahr*^−/−^ mice, but left open the possibility that activation of the AhR pathway in T or B lymphocytes is important for skin homeostasis.

**Figure 5:**
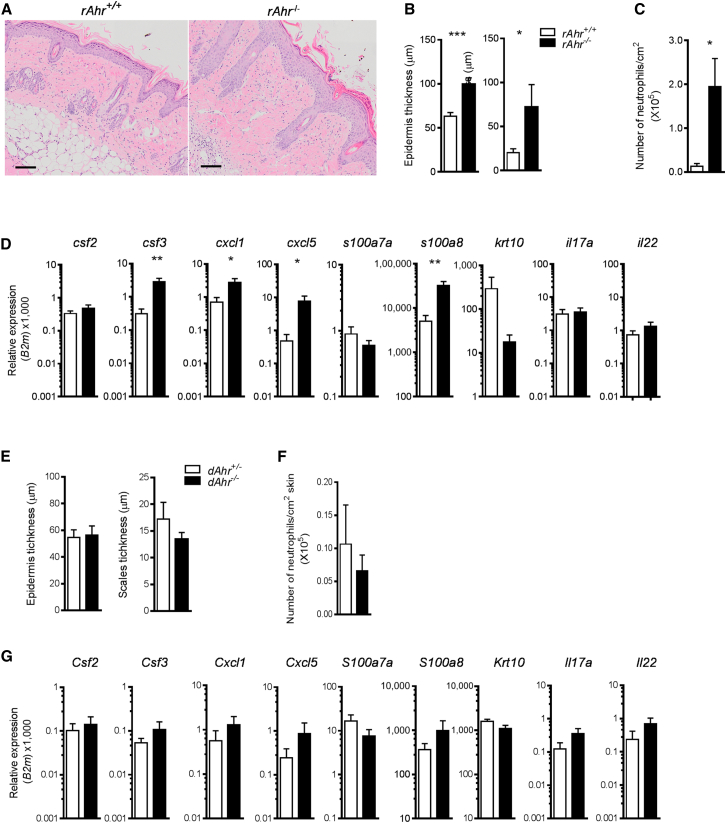
Lack of AhR in Nonhematopoietic Cells Recapitulates Exacerbated Skin Inflammation of *Ahr*^−/−^ Mice (A) Representative images of H&E staining of skin sections from IMQ-treated *Ahr*^+/+^ → *Ahr*^+/+^*Rag1*^−/−^ (rAhR^+/+^) or *Ahr*^+/+^ → *Ahr*^*−/−*^*Rag1*^*−/−*^ (rAhR^−/−^) BM chimeras (scale bars represent 100 μm). (B) Quantification of epidermal (left) and scale thickness (right) of rAhR^+/+^ (white bars) or rAhR^−/−^ (black bars) chimeras at day 5. (C) Number of neutrophils per cm^2^ of skin. (D) mRNA expression of psoriasis-relevant genes in whole skin from rAhR^+/+^ (white bars) or rAhR^−/−^ (black bars) chimeras at day 5. (E) Quantification of epidermal (left) and scale thickness (right) of *Ahr*^*+/−*^*→ Ahr*^*+/+*^*Rag1*^*−/−*^ (dAhR^+/−^, white bars) or *Ahr*^*−/−*^*→ Ahr*^*+/+*^*Rag1*^*−/−*^ (dAhR^−/−^, black bars) chimeras at day 5. (F) Number of neutrophils per cm^2^ of skin. (G) mRNA expression of psoriasis-relevant genes in whole skin from dAhR^+/−^ (white bars) or dAhR^−/−^ (black bars) chimeras at day 5. Plots show mean + SEM; n = 5–7 mice per group. Results from one representative experiment of two independent experiments per each set of chimeras are shown. ^∗^p < 0.05, ^∗∗^p < 0.01, and ^∗∗∗^p < 0.001.

Next, we addressed the contribution of *Ahr* deficiency in nonhematopoietic skin cells such as keratinocytes and fibroblasts. We generated bone marrow (BM) chimeras in which hematopoietic cells were of wild-type origin, whereas the nonhematopoietic compartment was either *Ahr*^−/−^ (rAhR^−/−^) or wild-type (rAhR^+/+^) by reconstituting *Ahr*^−/−^*Rag1*^−/−^ and control *Ahr*^*+/+*^ *Rag1*^−/−^ hosts with BM from *Ahr* wild-type donors. AhR deficiency in the nonhematopoietic compartment recapitulated the hyperinflammatory phenotype of full AhR^−/−^ mice with exacerbated epidermal pathology (Figures 5A and 5B), increased neutrophil recruitment (Figure 5C), overexpression of inflammatory markers, and reduced keratinocyte differentiation (Figure 5D). In contrast, expression of both IL-17 and IL-22 was not different in the two experimental groups, making it unlikely that these cytokines are responsible for the hyperinflammatory skin response of *Ahr*^−/−^ mice. In line with this, treatment of *Ahr*^−/−^ mice with neutralizing antibody to IL-17A did not improve their exaggerated response (Figures S4E–S4G).

BM chimeras in which the nonhematopoietic cells were of wild-type origin, whereas the hematopoietic compartment was either *Ahr*^+/−^ (dAhR^+/−^) or *Ahr* deficient (dAhR^−/−^), did not show increased inflammation above that observed in control mice (Figures 5E–5G). Disease severity was not increased in *Ahr*^−/−^*Rag1*^−/−^ versus *Ahr*^+/−^ *Rag1*^*−*/−^ (data not shown), emphasizing that the cross-talk between adaptive immune cells and epidermal cells is essential for the exacerbated pathology observed in *Ahr*-deficient mice. Therefore, AhR deficiency in the nonhematopoietic skin compartment is necessary and sufficient for the development of an exacerbated psoriasiform skin response in the presence of a fully functioning adaptive immune system.

### AhR-Deficient Keratinocytes Show Exacerbated Response to Proinflammatory Cytokines

**Figure 6:**
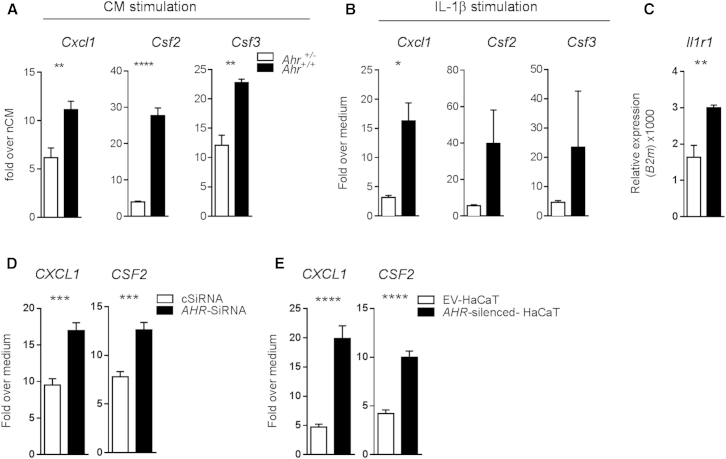
*Ahr-*Deficient Murine and Human Keratinocytes Show Exacerbated Response to Inflammatory Stimuli (A) mRNA expression of proinflammatory mediators in *Ahr*^+/−^ (white bars) and *Ahr*^*−*/−^ (black bars) murine keratinocytes stimulated for 24 hr with conditioned medium nCM and iCM. Data are expressed as fold change over stimulation with iCM over nCM medium. (B) mRNA expression of proinflammatory mediators in *Ahr*^+/−^ (white bars) and *Ahr*^−/−^ (black bars) murine keratinocytes stimulated for 24 hr with recombinant IL-1β (10 ng/ml). Data expressed as fold change over medium control. (C) *Il1r1* mRNA expression in unstimulated murine primary keratinocytes from *Ahr*^+/−^ (white bars) and *Ahr*^*−*/−^ (black bars) mice. (D) mRNA expression of proinflammatory mediators in human primary keratinocytes, transiently transfected for 48 hr with a nontargeting control SiRNA (cSiRNA, white bars) or in which AhR was transiently silenced (AhR-SiRNA, black bars), and stimulated for further 24 hr with human recombinant IL-1β (10 ng/ml). (E) mRNA expression of proinflammatory mediators in human keratinocytes HaCaT cell lines, stable transfected with an empty vector (EV-HaCaT, white bars), or in which AhR had been stable silenced (AhR-silenced HaCaT, black bars), and stimulated for 24 hr with human recombinant IL-1β. Data expressed as fold change over medium control. Results from one representative experiment of two or three independent experiments are shown. Plots show mean ± SEM; n = 3–6 wells per group. ^∗^p < 0.05, ^∗∗^p < 0.01, and ^∗∗∗∗^p < 0.0001.

Our results showed that physiological AhR activation can ameliorate the inflammatory program in nonhematopoietic skin cells. In order to discriminate between the inflammatory response of epithelial and stromal cells, we assessed the response of *Ahr*-sufficient or -deficient keratinocytes to the immune activators produced in the early phase of IMQ-induced skin inflammation. We therefore stimulated keratinocytes from *Ahr*^+/−^ and *Ahr*^*−*/−^ mice in vitro with conditioned medium (CM) from in-vitro-reactivated skin cells obtained from either naive (nCM) or 2-day IMQ-treated (iCM) wild-type mice. *Ahr*^−/−^ keratinocytes responded to iCM by significantly overexpressing proinflammatory cytokine and chemokine mRNA as compared to heterozygous controls (Figure 6A). *Ahr*^−/−^ fibroblasts also displayed an increased response as compared to their *Ahr*^+/−^ counterparts, although to a lesser extent (data not shown). IL-1β is one of the mediators in the early phase of the IMQ-induced skin inflammation model (Walter et al., 2013), and this cytokine was overrepresented in the conditioned medium obtained from wild-type IMQ-treated mice, far exceeding other cytokines tested such as TNF, IL-23, and IL-17A (data not shown). Indeed, recombinant IL-1β could replace iCM, causing comparable upregulation of inflammatory markers on keratinocytes (Figure 6B), whereas neutralizing IL-1β abrogated the proinflammatory response to the skin cell conditioned medium (Figure S5A). In addition, we also found increased expression of *Il1r1* in ex vivo *Ahr*^−/−^ keratinocytes (Figure 6C).

Furthermore, similar hyperresponsiveness was found in human keratinocytes upon knockdown of *AHR* expression. Thus, normal primary keratinocytes in which *AHR* expression was reduced by *AHR*-SiRNA showed overexpression of proinflammatory mediators compared to keratinocytes transfected with a nontargeting SiRNA (cSiRNA) (Figures 6D and S5B). Similar results were also obtained in the spontaneously transformed keratinocyte cell line HaCaT, in which *AHR* had been stably silenced (*AHR*-silenced HaCaT), when compared to cells transfected with an empty vector (EV-HaCaT) (Figure 6E; Fritsche et al., 2007).

Taken together, these results show that nonhematopoietic skin cells, particularly keratinocytes, require AhR to control expression of inflammatory mediators in response to inflammatory stimuli, such as IL-1β. Therefore we conclude that AhR deficiency in both epithelial and stromal cells results in a cell-intrinsic overreaction to inflammatory stimuli, leading to exacerbated skin pathology in vivo.

### AhR Modulates *JunB* Expression in Keratinocytes

**Figure 7:**
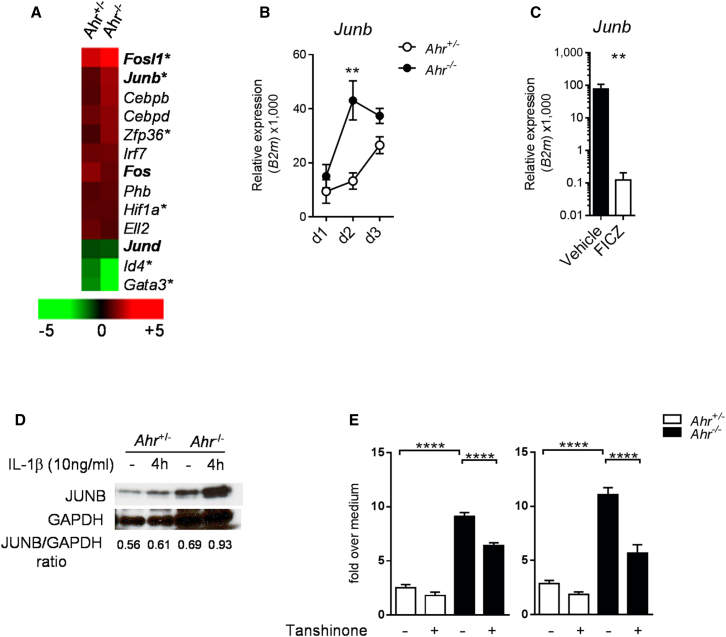
*Ahr*-Deficient Keratinocytes Display Increased Levels of JunB (A) Heat map visualization of IMQ-regulated, psoriasis-annotated transcription factors in whole skin of *Ahr*^+/−^ and *Ahr*^−/−^ mice on day 2 of IMQ treatment. Green color indicates decreased and red color increased gene expression, expressed as mean fold change in IMQ-treated as compared to corresponding untreated mice. AP-1 family transcription factors are marked in bold. Asterisks indicate genes differentially expressed in *Ahr*^*−*/−^ versus *Ahr*^+/−^ skin at day 2 of IMQ treatment. (B) Time course of *Junb* mRNA expression in whole skin from *Ahr*^+/−^ (open circles) and *Ahr*^*−/−*^ (filled circles) mice. (C) *Junb* mRNA expression in whole skin from IMQ-treated C57BL/6 mice receiving vehicle (black bars) or FICZ (white bars) at day 6. (D) Immunoblot showing JunB protein levels in *Ahr*^+/−^ (white bars) and *Ahr*^−/−^ (black bars) murine keratinocytes stimulated for 4 hr with recombinant IL-1β (10 ng/ml). Values denotes JunB/GAPDH densitometry ratio. (E) mRNA expression of *Csf2* and *Csf3* in murine keratinocytes stimulated for 24 hr with recombinant IL-1β (10 ng/ml) with or without Tanshinone (1 μM). Data expressed as fold change over medium control. Plots show mean ± SEM; n = 3–6 wells or mice per group. Results from one representative experiment of two independent experiments are shown. ^∗∗^p < 0.01, ^∗∗∗^p < 0.001, and ^∗∗∗∗^p < 0.0001.

The AhR is thought to be involved in extensive cross-talk with other transcription factors and multiple signaling pathways, which makes the systematic analysis of physiological interactions a challenging task. To gain more insights about how AhR modulates keratinocyte activation, we focused on the early phase of the IMQ treatment and performed microarray analysis of whole skin from *Ahr*^+/−^ or *Ahr*^−/−^ mice treated for 2 days with IMQ. By using Ingenuity pathway analysis, we identified a number of psoriasis-annotated transcription factors regulated by the IMQ treatment and found an overrepresentation of the activator protein-1 (AP-1) family of transcription factors (Figure 7A). AP-1 regulates a range of biological mechanisms, including keratinocyte differentiation and proliferation (Bata-Csorgo and Szell, 2012) and epithelial cell immune activation (Wang et al., 2013). Also, the AP-1 family member JunB, whose activity is transcriptionally regulated (Karin et al., 1997), is increased in psoriasis and localizes to keratinocyte nuclei within the hyperplastic epidermis of psoriasis lesions (Haider et al., 2006; Swindell et al., 2013). We found the expression of *Junb* significantly increased in the skin of *Ahr*^−/−^ mice as compared to *Ahr*^+/−^ mice in the early phase (day 2) of IMQ-induced inflammation (Figure 7B), whereas whole skin isolated from FICZ-treated wild-type mice showed reduced *Junb* levels (Figure 7C), suggesting that AhR can control the expression of this AP-1 family member. Moreover, isolated keratinocytes from *Ahr*^−/−^ mice significantly upregulated JunB protein (Figure 7D) and mRNA (Figure S6) in response to IL-1β and iCM. Finally, inhibition of the AP-1 pathway with the inhibitor Tanshinone IIA (Tseng et al., 2013) resulted in decreased expression of proinflammatory genes in *Ahr*^*−*/−^ keratinocytes (Figure 7E). These findings show that AhR controls the expression of other transcription factors responsible for establishing an inflammatory transcriptional program in keratinocytes, e.g., AP-1, and thus constitutes a crucial regulator in the development of skin inflammatory processes.

## Discussion

The contribution of environmental factors to the pathogenesis of inflammatory disorders is well known, but the nature and mode of action of such stimuli remains ill defined. We show here that environmental signals transmitted via AhR dampen the inflammatory response in both mouse and human skin. Lack of *Ahr* causes hyperinflammation, whereas deliberate AhR activation with the endogenous ligand FICZ ameliorates the inflammatory profile in both human psoriasis samples and the mouse model of psoriasiform skin inflammation. Our data emphasize the cross-talk between cells of the immune system and nonhematopoetic cells during inflammation, and it is now widely recognized that such interactions crucially underpin both the homeostasis of the skin environment and its dysregulation in diseases such as psoriasis (Di Meglio et al., 2011; Lowes et al., 2013). The focus for therapeutic intervention in psoriasis is currently on modulating inflammatory immune parameters such as IL-17, IL-12, IL-23, or TNF (Lowes et al., 2013), which are the immune drivers of skin pathology in both human disease and the mouse model. In agreement with the literature (Pantelyushin et al., 2012; Van Belle et al., 2012; van der Fits et al., 2009), we found many parameters linked to the IL-17 program highly upregulated in IMQ-induced skin inflammation and even further exacerbated in *Ahr*^−/−^ mice. However, our data show that immune cells are not the main cause for the hyperreactivity observed in *Ahr*-deficient mice. Instead, the response of nonhematopoietic skin cells, primarily keratinocytes but also skin fibroblasts, to inflammatory stimuli was severely dysregulated in the absence of AhR. However, in vivo blockade of this cytokine was not sufficient to dampen the exaggerated skin response of *Ahr*-deficient mice (data not shown), suggesting that multiple inflammatory pathways are involved.

It was important to delineate contributions by different cell types because it was suggested, for instance, that the epidermal TCR-γδ population, which interacts with keratinocytes (Chodaczek et al., 2012) and is absent in *Ahr*^−/−^ mice (Kadow et al., 2011; Li et al., 2011), may fulfil protective functions in cutaneous inflammation (Girardi et al., 2006; Kadow et al., 2011). However, mice with selective *Ahr* deficiency resulting from Rag1 Cre-mediated deletion also lack this population, yet did not display the widespread overreaction seen in *Ahr*^−/−^ skin although they displayed some abnormalities in the epidermis. All bone-marrow chimeras lacked epidermal γδ T cells because the hosts were Rag1 deficient and epidermal γδ T cells cannot be reconstituted by BM from adult mice, yet only those chimeras with *Ahr* deficiency in the nonhematopoietic compartment displayed the full phenotype seen in *Ahr*-deficient mice.

Both psoriasis pathogenesis in humans and the IMQ model of psoriasiform skin inflammation in the mouse are T cell dependent and rely on the cross-talk between adaptive immune cells and epidermal cells. Thus, it is not surprising that in the complete absence of effector T cells, such as in *Rag1*^*−*/−^ mice, lack of AhR in keratinocytes did not result in increased disease severity.

IL-23-producing myeloid DCs are critical for the IMQ model, whereas plasmacytoid DCs (pDCs) and the type I interferon pathway are dispensable in this mouse model (Wohn et al., 2013). We did not detect infiltration of pDCs in the skin (data not shown) and *Ahr* deficiency restricted to CD11c-expressing antigen-presenting cells did not recapitulate the hyperinflammation of *Ahr*^−/−^ mice, indicating that these cells, although important for establishing inflammation in the first place, were not drivers for the overreaction seen in *Ahr*^−/−^ mice. Of note is the effect of *Ahr* deletion in different cell types on expression of IL-17 and IL-22. In agreement with previous data (Martin et al., 2009), we found that complete *Ahr* deficiency resulted in higher levels of IL-17 probably due to the predominant infiltration of γδ T cells, which produce more IL-17 in *Ahr*-deficient mice. This effect, however, was not evident when *Ahr* deficiency was restricted to T and B cells, suggesting that additional interactions with other *Ahr*-deficient cells, e.g., APCs, contribute to IL-17 induction. Furthermore, in contrast to our previous demonstration that in-vitro-differentiated Th17 cells or γδ T cells require AhR stimulation for IL-22 production, IL-22 was readily detectable in the skin of *Ahr*-deficient mice. It is conceivable that the inflammatory milieu in the skin can provide other factors that could override the requirement for AhR. Finally, the systemic application of the AhR ligand FICZ caused a reduction rather than an increase in IL-17 and IL-22. This is in contrast to the effect of FICZ in vitro or in localized application during EAE, but consistent with its suppressive effect when administered systemically (Duarte et al., 2013).The complexities of cellular interactions in an inflammatory environment that shape these variables require further dissection. However, in the context of our study, we contend that IL-17 and IL-22, although important for the development of psoriasiform inflammation, are not the reason for the exacerbated response of *Ahr*-deficient mice.

The IMQ model of psoriasiform skin inflammation in mice and the pathogenesis process in psoriasis have different kinetics, inflammatory components, and cellular mediators, but nevertheless share critical immunopathological features. Although dispensable for the IMQ model, both type I and II interferon pathways play an important role in human psoriasis (Bowcock et al., 2001). Type I IFN is critical in the early phases of disease initiation in a clinically relevant skin xenotransplant model where it triggers activation and expansion of autoimmune T cells, leading to fully fledged psoriasis plaque formation (Nestle et al., 2005).

A strong IFN-γ genomic (Bowcock et al., 2001) and cellular (Austin et al., 1999) signature is present in psoriasis, and intradermal injection of IFN-γ has been shown to induce several molecular and histological features characteristic of psoriatic lesions in both healthy and psoriatic human skin (Johnson-Huang et al., 2012). *AHR* ligation in human skin biopsies strikingly modulated type I and II interferon pathways, particularly normalizing the proinflammatory signature present in L skin. On the other hand, pharmacological blockade of the *AHR* pathway in ex vivo human skin biopsies and genetic deletion of *Ahr* in the mouse model resulted in an exacerbation of the inflammatory skin signature, whereas activation of the pathway ameliorated both.

Perhaps not surprisingly, human and mouse data did not show mechanistic similarities in terms of genes or pathways affected by AhR. This could be due to the very different ontogeny of the inflammatory skin response, which is provoked by a single agent and short lasting in the mouse, whereas it is multifactorial and chronic in patients. In contrast, the lack of effect of AhR-mediated activation on other relevant psoriasis gene signatures in human samples, such as the antimicrobial response and tissue remodeling, which are affected in the mouse model, could be the result of the short-term culture of the skin biopsies. However, both murine and human keratinocytes lacking AhR were overreactive to proinflammatory stimuli, suggesting shared aspects of pathology, which may diverge later on in the chronic phase of human psoriasis.

It remains to be clarified what the direct targets of AhR are and how these are linked to the inflammatory networks that are affected. The postulated extensive interaction of AhR with other transcription factors, which may be cell type specific, presents a substantial challenge in identifying direct as well as indirect AhR-mediated effects in inflammatory responses. We found dysregulation of the expression of the AP-1 family member *Junb*, which was substantially upregulated in the inflamed skin of *Ahr*-deficient mice and could be readily induced in in vitro keratinocytes stimulated with IL-1β. Moreover, blocking the AP-1 pathway dampened the increase of proinflammatory genes in *Ahr*-deficient keratinocytes. Whereas deletion of *Junb* together with *Jund* in mouse keratinocytes resulted in a skin phenotype resembling psoriasis (Zenz et al., 2005), *JUNB* is upregulated in psoriatic skin (Haider et al., 2006) and a recent comprehensive meta-analysis of the psoriasis transcriptome has revealed an enrichment of AP-1-binding sites among psoriasis-increased genes and pinpointed the increased expression of *JUNB* in psoriasis skin to keratinocytes (Swindell et al., 2013), suggesting a role for the AP-1 pathway and JunB as a critical checkpoint of epidermal homeostasis.

The emerging pattern from analysis of physiological functions of AhR in immune cell types indicates that it affects radically different gene patterns in different cells types (Kiss et al., 2011; Lee et al., 2012; Li et al., 2011; Nguyen et al., 2010; Qiu et al., 2012; Veldhoen et al., 2008), suggesting that AhR may be a modulator of gene expression with different target genes. In keratinocytes, AhR signaling favors epidermal differentiation, thus promoting skin barrier formation (van den Bogaard et al., 2013) and as shown here AhR signaling exerts an anti-inflammatory effect in keratinocytes. More research will be needed to identify the molecular mechanisms underlying the function of AhR in the control of skin homeostasis during inflammatory responses.

Under physiological conditions, AhR signaling appears to be tightly regulated and endogenous ligands such as FICZ are rapidly metabolized via the activity of CYP enzymes that are downstream targets of AhR activation, most notably CYP1A1, the main extrahepatic cytochrome p450 enzyme under control of the AhR (Wincent et al., 2009). In fact it is now assumed that prolonged AhR signaling in response to TCDD, for example, causes dysregulation of its physiological functions (Bock and Köhle, 2006; Mitchell and Elferink, 2009). In keeping with this, expression of a constitutive active form of AhR in keratinocytes caused skin lesions (Tauchi et al., 2005), and we and others (van den Bogaard et al., 2013) have shown beneficial consequences of physiological AhR activation in skin. Interestingly, kynureninase (*KYNU*), an enzyme of the tryptophan catabolism degrading the putative AhR agonist kynurenine, is one of the consistently upregulated genes in psoriatic skin in our data set and in the literature (Tian et al., 2012), and other genes of the tryptophan pathway have also been found to be differentially regulated in psoriatic skin (Gudjonsson et al., 2010). Increased levels of KYNU enzyme might not only reduce kynurenine levels in psoriastic skin but also deplete tryptophan in the tissue, thus interfering in the formation of other AhR ligands such as FICZ. It is possible that deregulation in tryptophan catabolism observed in psoriatic skin might decrease AhR activation and thus result in increased expression of inflammatory mediators in the skin. Thus, reduced levels of endogenous AhR ligands in psoriatic skin might account for the lack of AhR-mediated control of skin homeostasis. It is important also to consider that dietary ligands, which are of fundamental importance in the AhR-mediated control of intestinal homeostasis, may exert effects in tissues other than the gastrointestinal tract. It is worth mentioning that metabolic syndrome and Crohn’s disease are frequently observed comorbidities of psoriasis (Davidovici et al., 2010).

Thus, beneficial effects of AhR activation open the possibility of therapeutic intervention in chronic inflammatory skin disease, but further research is needed to understand the mechanism underlying the physiological consequences of AhR signaling in the immune system.

## Experimental Procedures

Details on human skin biopsy culture, animals, RNA isolation, qPCR, RNA sequencing, microarray analysis, and keratinocyte cultures are listed in Supplemental Information.

### Human Subjects

Lesional and nonlesional psoriasis skin biopsies were obtained from patients of European descendent recruited at the Psoriasis Center, University Medical Center Schleswig-Holstein, Kiel (Germany) and not receiving any systemic treatment at the time of visit. Four skin biopsies were obtained from discarded healthy skin from donors of European descendent undergoing plastic surgery procedures at Guy’s and St. Thomas’ Hospital, London (UK). Full patient and healthy control demographics are in Table S1. Our study was conducted in accordance with the Helsinki Declaration, with written informed consent obtained from each volunteer, and approved by the institutional review board of University of Kiel Medical School and Guy’s and St. Thomas’ Hospital.

### Imiquimod Model of Psoriasiform-like Skin Inflammation

Shaved mouse dorsal skin was treated daily for 5 consecutive days with 30 mg Aldara cream containing 5% Imiquimod (IMQ, Meda AB). On day 5, full-thickness skin biopsies of the treated area were collected with a 8 mm biopsy puncher; skin was either snap frozen in liquid N_2_ for RNA extraction, fixed in neutral buffered formalin (Sigma) for histopathology analysis, or digested as described below to achieve single-cell suspensions. In some experiments, wild-type C57BL/6 mice received vehicle (olive oil) or 100 μg/kg FICZ (Enzo) intraperitoneally on the day before starting the IMQ treatment and then daily until the day of analysis.

### Skin Histopathology

Fixed skin was embedded in paraffin and tissue sections were deparaffinized and stained with H&E for histological analysis. Images were acquired at ×10 magnification with an Olympus VS120 slide scanner. Average epidermal and scale thickness was quantified by a researcher blind to the experimental groups who took five measurements per three sections for each mouse.

### Flow Cytometry Analysis of Skin-Infiltrating Cells

Two 8 mm punch biopsies were minced and shook in a digestion cocktail (400 μg/ml Liberase TL [Roche] and 1 mg/ml collagenase D [Roche] in IMDM medium) for 2 hr at 37°C, then mashed through a 70 μl cell strainer. Flow cytometry was performed with antibodies by Biolegend and cells were acquired on a FACSCantoII (BD). For intracellular cytokine staining, cells were stimulated for 4 hr with PdBU (500 ng/ml) and ionomycin (500 ng/ml) in the presence of brefeldin A (1 μg/ml) and Fc block (BD Bioscience), while simultaneously stained for surface markers, then fixed with 3.8% PFA, permeabilized with 0.1% NP-40, and stained for IL-17A and IL-22 (Biolegend).

### Conditioned Medium from Skin Cell Suspension of Naive or IMQ-Treated Wild-Type Mice

Conditioned media was obtained from supernatants of skin cell cultures from either naive mouse skin or from skin of wild-type mice treated with IMQ for 2 days: after obtaining single-cell suspensions as described earlier, cells were activated with PdBU and ionomycin for 30 min at 37°C in RPMI medium supplemented with 10% fetal calf serum and 2% Pen-Strep-Gln solution (cRPMI), washed to remove PdBU and ionomycin, and incubated in cRPMI for 3 hr at 37°C; the resulting cell-free supernatant was then used as conditioned media.

### Total Cellular Extract and Immunoblot

Primary keratinocytes were washed two times with ice-cold PBS and lysed with NP40 cell lysis buffer (Life Technologies). Protein concentration was determined by the Bio-Rad protein assay kit (Bio-Rad). Cell lysates (20 μg) were separated by SDS-PAGE, transferred onto PVDF membranes (GE Healthcare), and probed with a primary antibody against mouse-JunB (1:1,000, Cell Signaling) and then with anti-rabbit immunoglobulin coupled to peroxidase (1:10,000; GE Healthcare). The immune complexes were visualized by the enhanced chemiluminescence method (Merck Millipore), and results were analyzed by Adobe Photoshop software and normalized to GAPDH.

### Statistical Analysis

Statistical analysis was performed with Prism v.5.0 (GraphPad Software). For in vivo experiments, values are expressed as the mean + SEM of *n* animals and data shown are representative of at least two independent experiments. Comparisons were calculated by unpaired t test, if two groups were assessed, or one-way analysis of variance and Bonferroni-corrected p value for multiple comparisons, if more than two groups were assessed. For qPCR validation of human RNA sequencing, fold changes of treatment versus vehicle control-induced gene expression were assessed for normal Gaussian distribution with D’Agostino & Pearson omnibus normality test and then analyzed by paired two-tailed t test or Wilcoxon signed rank test, as appropriate and shown as box and whiskers (Min-Max). The level of statistically significant difference was defined as p ≤ 0.05.

## Author contributions

P.D.M. and J.H.D. jointly designed and performed the experiments, analyzed and interpreted the data, and wrote the manuscript.

## Accession Numbers

The microarray data are available in the Gene Expression Omnibus (GEO) database (http://www.ncbi.nlm.nih.gov/gds) under the accession number GSE47607. The RNA-seq data are available under the accession number GSE47944.

## Supplemental Information

Document S1. Tables S1–S3, Figures S1–S6, and Supplemental Experimental Procedures

Document S2. Article plus Supplemental Information
