# Supplementary material for: Activation of the Aryl Hydrocarbon Receptor Dampens the Severity of Inflammatory Skin Conditions
Source: Immunity. 2014 Jun 19;40(6):989–1001. doi: 10.1016/j.immuni.2014.04.019 (PMC4067745; doi:10.1016/j.immuni.2014.04.019)
Supplement: Document S1. Tables S1–S3, Figures S1–S6, and Supplemental Experimental Procedures [file mmc1.pdf]

Immunity, Volume *40*

Supplemental Information

Activation of the Aryl Hydrocarbon Receptor Dampens the Severity of Inflammatory Skin Conditions

Paola Di Meglio, João H. Duarte, Helena Ahlfors, Nick D.L. Owens, Ying Li, Federica Villanova, Isabella Tosi, Keiji Hirota, Frank O. Nestle, Ulrich Mrowietz, Michael J. Gilchrist, and Brigitta Stockinger

Di Meglio et al.

Supplemental Information

Inventory:

Supplemental Data

Supplemental Table 1, related to Figure 1

Supplemental Table 2, related to Figure 1

Supplemental Table 3, related to Figure 1

Supplemental Figure 1, related to Figure 1

Supplemental Figure 2, related to Figure 2

Supplemental Figure 3, related to Figure 3

Supplemental Figure 4, related to Figure 4

Supplemental Figure 5, related to Figure 6

Supplemental Figure 6, related to Figure 7

Supplemental Experimental Procedures

Supplemental References

Table S1

Sample ID	Age (yrs)	Gender	PASI	Age of Onset	Treatment
**H1**	54	F	NA	NA	NA
**H2**	53	F	NA	NA	NA
**H3**	74	F	NA	NA	NA
**H4**	36	F	NA	NA	NA
**H5**	37	F	NA	NA	NA
**P1**	56	M	4.2	35	topical steroids+Vitamin D
**P2**	52	M	7.9	51	topical steroids+Vitamin D
**P3**	63	F	4.1	53	topical steroids+Vitamin D
**P4**	56	F	2.8	31	topical steroids+Vitamin D
**P5**	30	M	12.5	24	topical steroids
**P6**	56	F	4.2	53	topical Vitamin D
**P7**	60	M	15.6	44	topical steroids+Vitamin D
**P8**	45	M	9.6	38	topical steroids+Vitamin D

Table S1 related to Fig.1: Demographics of healthy donors (H) and psoriasis patients (P). PASI: Psoriasis Area Severity Index; NA: Not applicable

Table S2

Increased expression in Lesional Skin				Decreased expression in Lesional Skin			
**Gene**	**FC: L vs NL**	**p**	**FDR**	**Gene**	**FC: L vs NL**	**p**	**FDR**
S100A9	113.785	2.41E-73	4.37E-69	ZSCAN18	0.273345	4.96E-52	3.15E-48
S100A8	105.068	2.76E-73	4.37E-69	PHF17	0.386304	7.10E-41	2.51E-37
TCN1	192.55	9.32E-71	9.86E-67	IL37	0.0528742	6.59E-38	1.90E-34
TPBG	3.75752	3.61E-56	2.86E-52	TMEM99	0.264457	2.18E-37	5.76E-34
S100A7A	1050.83	2.37E-48	1.25E-44	RP11-54F2.1	0.137792	1.33E-32	2.49E-29
C10orf99	15.8586	7.96E-45	3.61E-41	AQP9	0.127827	1.54E-31	2.57E-28
GJB2	17.0579	5.51E-42	2.19E-38	RORC	0.129265	2.84E-31	4.29E-28
ADAMDEC1	19.2404	1.10E-39	3.50E-36	C1orf95	0.207568	1.96E-29	2.59E-26
KYNU	30.0741	2.19E-36	5.35E-33	F3	0.188385	2.12E-29	2.69E-26
GLYCTK	2.59476	6.06E-36	1.37E-32	C7orf41	0.392176	2.24E-28	2.73E-25
FAM110C	3.86474	9.93E-33	2.05E-29	ZNF134	0.571151	2.92E-28	3.44E-25
CXCR6	8.189	1.03E-32	2.05E-29	KRT77	0.0426745	6.26E-28	7.10E-25
S100A7	62.428	9.70E-32	1.71E-28	CDH20	0.188352	3.31E-27	3.39E-24
S100A12	155.573	1.92E-31	3.04E-28	ANKRD33B	0.123088	6.69E-27	6.44E-24
IRAK2	3.02766	5.24E-31	7.57E-28	HS3ST6	0.099026	3.41E-26	3.00E-23
PLBD1	3.58343	1.10E-29	1.52E-26	GJB4	0.0621355	1.61E-25	1.33E-22
PI3	172.237	9.53E-28	1.04E-24	ACAT1	0.522441	1.64E-25	1.33E-22
TRIM14	2.74193	2.52E-27	2.67E-24	WNK2	0.152976	2.46E-25	1.91E-22
ARNTL2	3.4689	3.63E-27	3.60E-24	RAB3B	0.0944153	3.38E-25	2.49E-22
CCRN4L	3.04026	7.40E-27	6.91E-24	RGMB	0.346188	3.68E-25	2.65E-22
MPZL2	4.98868	8.31E-27	7.53E-24	CLDN1	0.283213	7.86E-25	5.20E-22
PRKCQ	11.3831	8.95E-26	7.68E-23	UGT3A2	0.032518	9.23E-25	5.98E-22
CHFR	1.4794	2.22E-25	1.76E-22	IGFL3	0.308453	1.31E-24	8.31E-22
ABCG4	25.4933	3.16E-25	2.38E-22	SOX5	0.264699	1.44E-24	8.98E-22
TMPRSS11D	81.6717	3.92E-25	2.77E-22	ANKH	0.51391	4.68E-24	2.61E-21

Table S2 related to Fig.1: Top 25 most significantly up- (left) and down-(right) regulated genes in lesional (L) *versus* non lesional (NL) psoriasis skin. FC: Fold Change; FDR: False Discovery Rate

Table S3

Gene	p: L+Ag	p: L+Ant	p: NL+Ag	p: NL+Ant	p: H+Ag	p: H+Ant	FC: L+Ag	FC: L+Ant	FC: NL+Ag	FC: NL+Ant	FC: H+Ag	FC: H+Ant	FC: L vs NL	FC: L vs H	FC: NL vs H	p: L vs NL	FDR: L vs NL
IFIT1	3.48E-03	0.0108741	0.0170926	0.000227536	0.0225637	0.400104	0.271201	4.67802	0.521284	3.94389	0.299311	1.65426	3.98471	4.12642	1.03556	0.010481	0.0483474
RSAD2	0.0125038	0.0475	0.00356989	0.000657162	0.00647929	0.837316	0.311598	3.18077	0.355403	4.62689	0.141145	1.16174	6.63058	6.82605	1.02948	0.00259334	0.0155029
IFIT3	0.00178354	0.008592	0.0334997	0.000150454	0.0161344	0.542856	0.32585	4.28298	0.579456	4.00026	0.261064	1.45715	3.32806	3.5218	1.05821	0.000493625	0.00398706
CMPK2	5.75E-03	0.0307245	3.27E-02	0.000829602	1.85E-02	0.349753	0.416924	2.89875	0.620467	3.15291	0.313114	1.66452	5.70972	4.95197	0.867287	0.000482138	0.0039072
MX2	0.00441559	0.015765	0.000402428	0.000383315	0.0762318	0.38643	0.440633	2.92039	0.571176	2.98869	0.432097	1.57498	2.83934	2.57779	0.907883	1.75E-03	1.13E-02
CXCL11	0.0787357	0.00260271	0.0533645	0.0409604	0.00987624	0.850044	0.538549	4.89391	0.324067	2.91547	0.10063	1.16908	2.85354	4.28917	1.50311	0.0137723	0.0601424
ISG15	0.0526282	0.01692	0.02408	0.000277227	0.00478694	0.305322	0.580267	2.69065	0.621024	3.55862	0.325571	1.61215	5.20431	4.78882	0.920165	0.00015084	0.001471
BATF2	0.0376235	0.106463	0.0416507	0.0184969	0.0235779	0.398252	0.583251	1.68586	0.650493	1.91794	0.417733	1.47454	6.71214	7.88844	1.17525	2.14E-09	8.48E-08
USP18	0.0900872	0.0201236	0.16868	0.00039072	0.0219202	0.351794	0.632513	2.5312	0.789233	2.66018	0.368804	1.56976	2.33296	2.83589	1.21557	0.00191217	0.0120768
EPSTI1	0.0533567	0.0372977	0.200401	0.0106382	0.119217	0.569919	0.654629	1.9546	0.750517	1.96289	0.536965	1.32026	5.14566	4.04371	0.785849	2.85E-07	6.56E-06
MX1	0.155754	0.0245986	0.172298	0.00371374	0.0597084	0.345131	0.655059	2.42122	0.728437	2.31971	0.49316	1.47952	8.45471	7.99345	0.945444	1.23E-06	2.35E-05
HERC5	0.0490263	0.0153939	0.107631	0.00189839	0.0431622	0.412223	0.66046	2.33987	0.767212	2.13846	0.488092	1.43281	1.6389	1.44528	0.88186	0.00560793	0.0291585
SAMD9L	0.0719968	0.0139822	0.169597	0.00327813	0.0738712	0.62234	0.660822	2.45586	0.783635	2.1843	0.543905	1.25636	1.88143	1.85172	0.984211	1.48E-06	2.77E-05
OAS2	0.134484	0.03811	0.171003	0.00644379	0.0339668	0.34688	0.664964	2.07892	0.691782	2.47631	0.437252	1.53101	12.1097	9.52759	0.786774	3.50E-06	5.84E-05
OAS3	0.0859894	0.0415683	0.265737	0.00674979	0.0480101	0.475588	0.726269	1.76326	0.810531	2.00849	0.520991	1.29722	5.58755	6.44058	1.15267	1.22E-15	1.76E-13
OAS1	0.20419	0.0480811	0.358342	0.0175433	0.0375915	0.276218	0.751428	1.8323	0.831491	1.79828	0.539406	1.47208	4.36701	5.42686	1.2427	2.02E-06	3.61E-05
IFI35	0.135327	0.0161809	0.468571	0.00477355	0.00870422	0.346032	0.752037	2.26911	0.895073	1.93329	0.511845	1.36559	1.47068	1.63651	1.11275	0.000363791	0.0030786
SAMHD1	0.145706	0.0490059	6.44E-01	0.00764016	0.156807	0.519317	0.753535	1.76536	0.911706	1.88208	0.609311	1.31541	1.73972	0.858043	0.493209	2.54E-06	4.40E-05
DDX58	0.175383	0.0150247	0.0923704	0.0024866	0.0816268	0.667979	0.757376	2.24734	0.802057	1.84373	0.634436	1.17508	2.1968	2.43703	1.10935	0.000123005	0.00124191
CXCL10	0.487771	0.00328835	0.0135834	0.00924241	0.0021157	0.928375	0.79966	3.52321	0.274128	4.07699	0.0399647	1.08999	4.2683	4.21451	0.987399	3.26E-04	2.80E-03
IFIT5	0.152304	0.0311479	0.125576	0.00158227	0.398726	0.379979	0.810979	1.66286	0.839452	1.79556	0.826243	1.37541	1.7717	1.5422	0.870465	0.000172904	0.00165117
IFIH1	0.240949	0.0207676	0.0581865	0.00576315	0.0889081	0.594458	0.826696	1.60736	0.790982	1.55465	0.635887	1.22203	2.79037	2.7434	0.983168	3.66E-07	8.16E-06
PARP9	0.483088	0.0495434	0.550367	0.0263906	0.283422	0.699644	0.885571	1.58936	0.909073	1.53066	0.774291	1.12674	3.74152	3.46242	0.925403	1.31E-13	1.29E-11
TRIM21	0.421847	0.0361962	0.8446	0.0100575	0.0988905	0.172658	0.898907	1.44382	0.987141	1.31866	0.813844	1.26434	1.746	1.59127	0.911378	1.07E-07	2.76E-06
SP100	0.297059	0.0373078	0.736999	0.0171899	0.978099	0.720029	0.907692	1.28074	0.977784	1.2972	0.996368	1.07463	1.46259	1.58434	1.08325	1.46E-04	1.43E-03
PML	5.14E-01	0.0380827	5.45E-01	0.0357884	0.00216225	0.675957	0.947273	1.1998	0.951043	1.24419	0.730519	1.05116	1.90067	1.96576	1.03424	1.11E-14	1.36E-12
ABCA12	0.871735	0.0415706	0.567731	0.02446	0.8455	0.776657	0.975855	0.781377	1.06889	0.738881	1.02829	0.947053	2.40896	2.22326	0.922914	1.42E-08	4.65E-07
CERS3	0.860371	0.0470667	0.68809	0.0455622	0.760594	0.507273	0.98282	0.838116	1.03718	0.794505	0.965676	0.904386	1.75026	1.80434	1.03089	5.52E-07	1.16E-05
MAP3K9	0.940543	0.00437561	0.697828	0.034043	0.853444	0.959826	0.988056	0.683579	1.04274	0.764078	0.97056	1.00978	1.56707	1.71202	1.09249	1.22E-04	1.23E-03
ITSN2	0.862977	0.0262858	0.718483	0.0345619	0.301638	0.446254	1.01603	0.867357	1.02797	0.843763	1.12197	0.844362	1.8204	2.09055	1.1484	1.49E-13	1.45E-11
PNPT1	0.798705	0.0434721	0.72886	0.0033737	0.388444	0.660447	1.03467	1.50751	0.963264	1.54112	0.822087	1.15757	1.6149	1.62388	1.00556	0.000670717	0.0051315
SDR9C7	0.757351	0.00377752	0.27857	0.00644368	0.61257	0.568835	1.04368	0.673535	1.1808	0.642406	0.938203	0.923249	2.20369	2.07802	0.942974	2.84E-07	6.54E-06
EPN3	0.581481	0.0308122	0.0381057	0.0271062	0.11925	0.931338	1.10236	0.700177	1.29464	0.747489	0.784158	1.01736	1.75086	1.84926	1.0562	6.74E-03	3.38E-02
C17orf109	0.0388934	0.187718	0.00686563	0.000135386	0.353922	0.185742	1.40405	0.832905	1.42698	0.614318	0.827568	0.74017	1.4519	1.4531	1.00083	3.87E-03	2.15E-02
ANO8	0.00537266	0.585833	0.000286363	0.0311761	0.668587	0.458632	1.41476	0.947504	1.45735	0.816553	0.955439	0.880626	0.648481	0.840267	1.29575	0.0099654	0.0463966
CD226	0.0453382	0.0383645	0.00292798	0.507886	0.0918451	0.152844	1.54971	0.652754	2.2563	0.858376	1.53213	0.577957	3.99776	2.4301	0.607865	1.52E-13	1.46E-11
AHRR	4.93E-05	0.000454801	7.83E-05	0.00151583	2.67E-05	0.0202052	1.7016	0.616542	1.63431	0.614336	1.6939	0.790912	0.614884	0.705078	1.14668	5.85E-04	4.59E-03
AC007639.1	0.00094916	0.453371	0.00354623	0.00445649	0.0160581	0.153311	1.71587	0.890429	1.41016	0.742814	1.49826	0.831918	1.62255	1.72446	1.06281	0.000639772	0.0049292
SLC45A4	3.91E-02	0.884789	9.58E-05	2.05E-03	2.14E-01	0.0260426	1.82936	0.970944	1.67703	0.640865	1.19485	0.673148	0.516201	0.60455	1.17115	0.00280904	0.0165431
SECTM1	7.74E-05	0.0741765	0.0105874	0.291886	0.569507	0.106064	1.85991	1.48154	1.40952	0.877685	1.09692	0.754916	1.42806	0.936017	0.655444	0.0421514	0.145896
CA4	0.0198649	0.110305	0.00126367	0.914333	0.00808135	0.761636	1.958	0.567383	2.17619	1.03372	2.73737	1.17579	0.569679	0.840651	1.47566	0.0320201	0.117859

Table S3 related to Fig.1: Psoriasis-relevant genes regulated by ex vivo ligation of AhR in human skin

FC: Fold change; Ag: Agonist; Ant: Antagonist; H: Healthy; L: lesional psoriasis skin; NL: non-lesional psoriasis skin; p: *p* value; FDR: False Discovery Rate

Figure S1

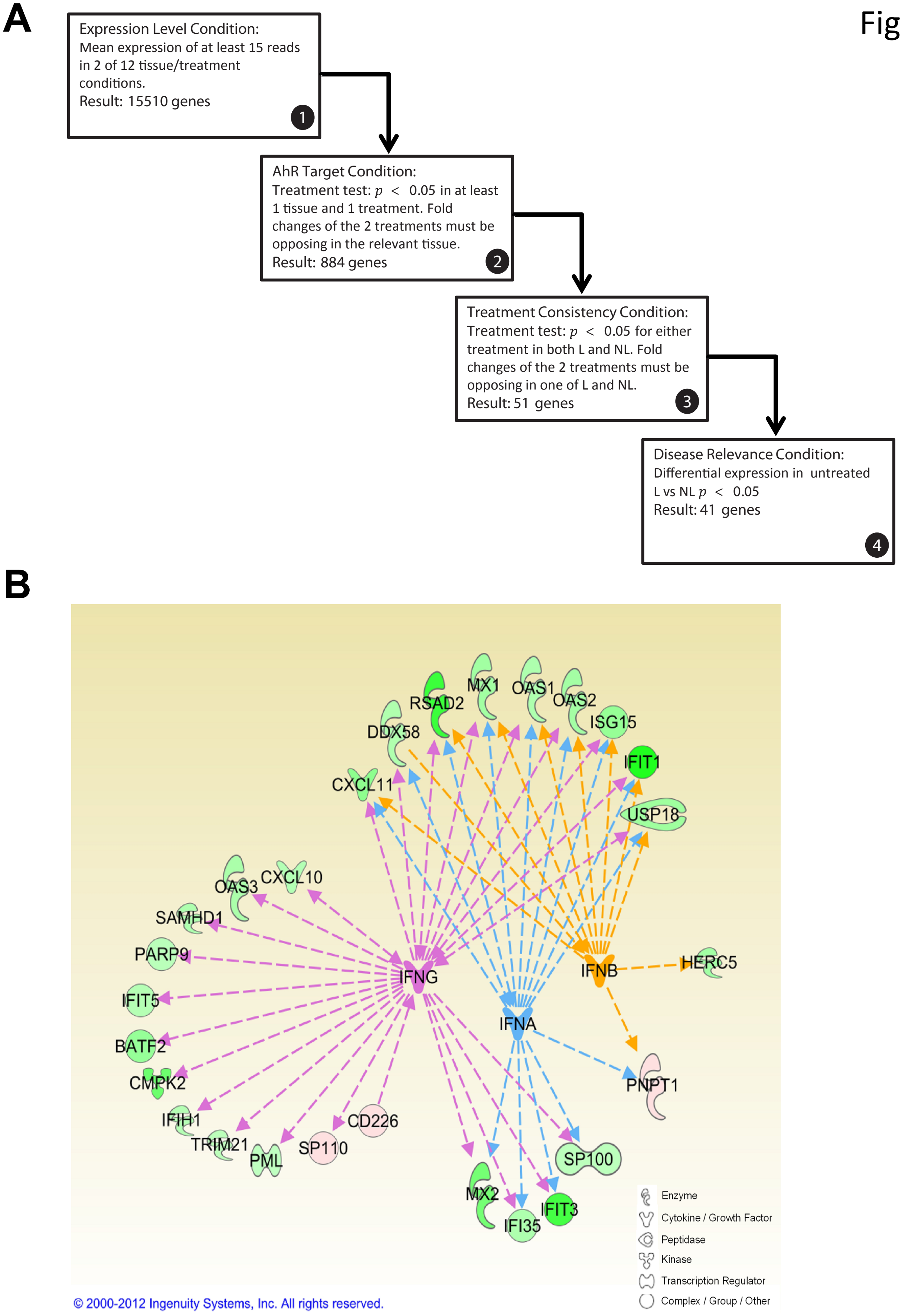

Figure S1 related to Fig.1: (A) Flow chart showing filtering criteria for RNA sequencing gene expressing analysis. (B) Network showing the molecular relationships between gene products modulated by AhR agonist in lesional skin. Genes are represented as nodes and their biological relationship is represented as an edge; the intensity of the node colour indicates the degree of up- (red) or down- (green) regulation

Figure S2

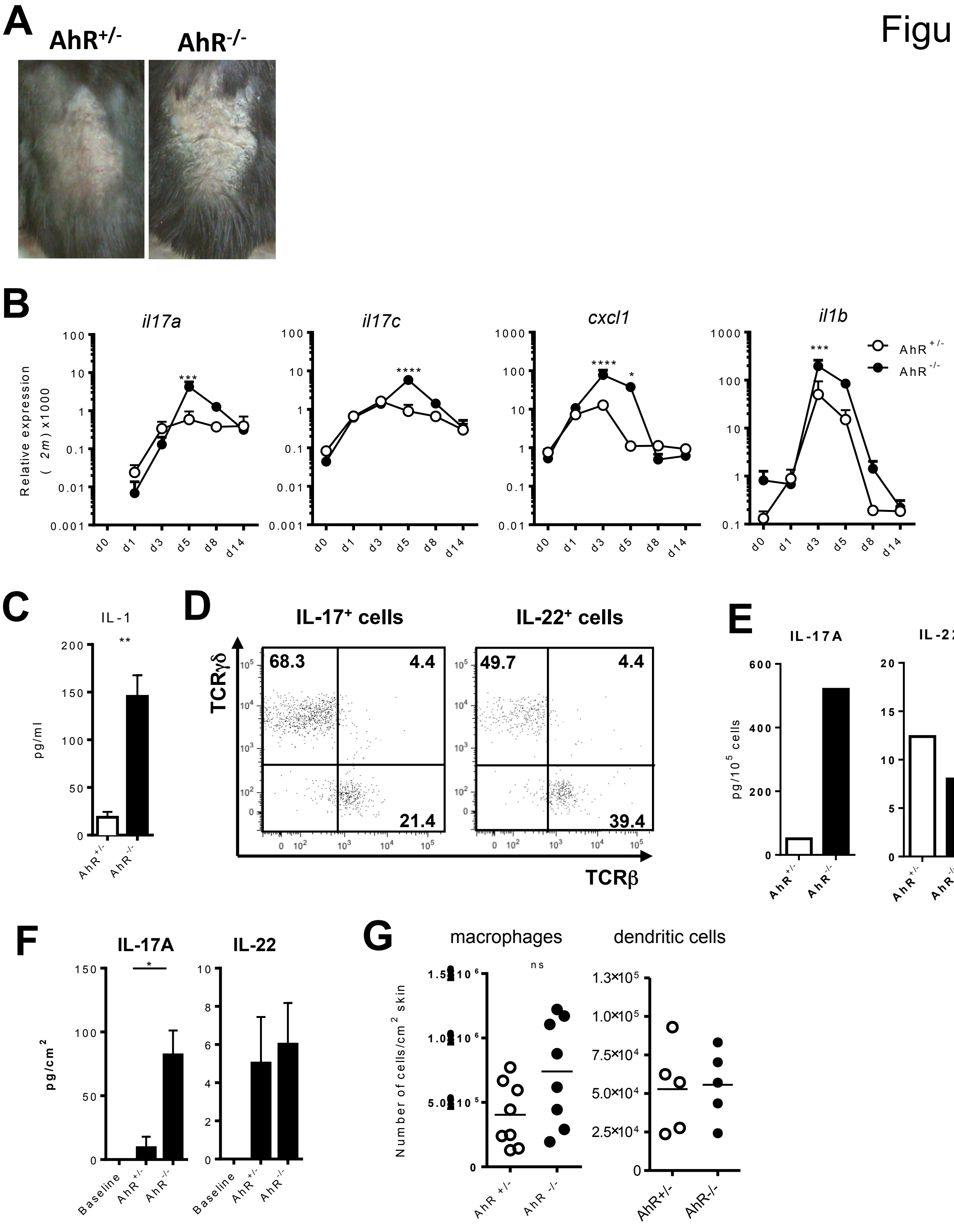

Figure S2 related to Fig.2: (A) Back skin of imiquimod-treated AhR$^{+/-}$ and AhR$^{-/-}$ mice 5 days after treatment initiation. (B) Kinetics of proinflammatory gene expression in whole-skin from imiquimod-treated AhR$^{+/-}$ and AhR$^{-/-}$ mice. (C) IL-1β protein levels from Pdbu/ Ionomycin-restimulated skin cell suspension from day 5 imiquimod-treated mice. (D) FACS dot plot showing expression of TCRβ and TCRγδ in IL-17 (left) and IL-22 (right) - expressing cells in the skin of day 5 imiquimod-treated AhR$^{+/-}$ mice. (E) IL-17A and IL-22 protein levels produced by Pdbu/Ionomycin-restimulated FACS-sorted eYFP from day 5 imiquimod-treated IL-17A fate reporter mice. (F) IL-17A and IL-22 protein levels on whole skin lysates from day 5 imiquimod-treated mice. (G) Number of macrophages (CD11b $^{+}$F4/80^{+}) and dendritic cells (CD11c^{+}) found in imiquimod-treated skin on day 5 after initiating treatment.

Figure S3

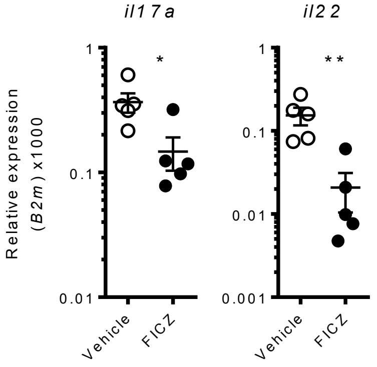

Figure S3 related to Fig.3: mRNA expression of *il17a* and *il22* in skin of imiquimod-treated mice with or without daily FICZ administration on day 5 after initiating treatment.

Figure S4

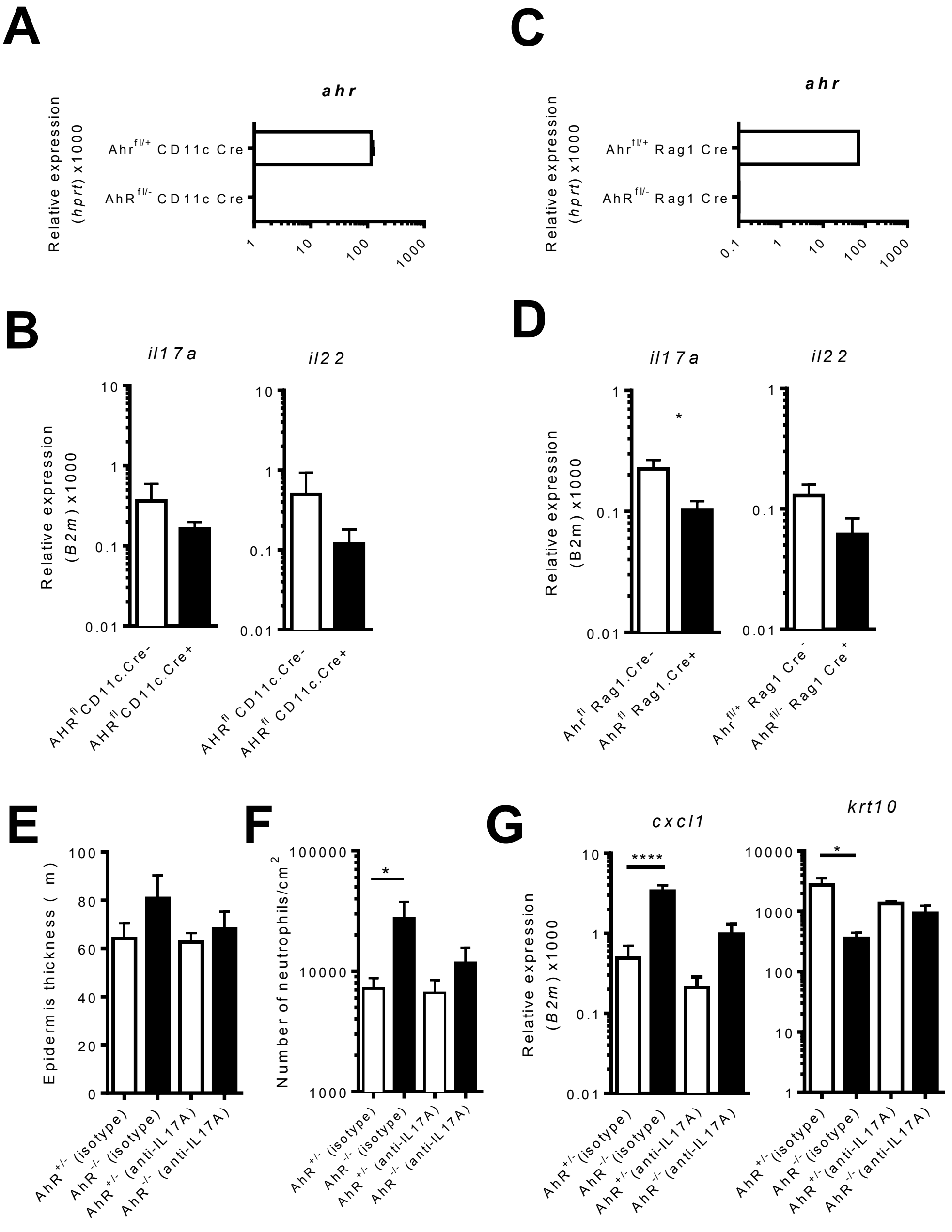

Figure S4 related to Fig.4: (A) qPCR showing deletion of AhR in sorted dendritic cells from AhR^{fl}CD11c.Cre mice. (B) mRNA expression of *il17a* and *il22* in skin of imiquimod-treated AhR^{fl}CD11c.Cre mice on day 5 after initiating treatment. (C) qPCR showing deletion of AhR in sorted T cells from AhR^{fl}CD11c.Cre mice. (D) mRNA expression of *il17a* and *il22* in skin of imiquimod-treated AhR^{fl}Rag1.Cre mice on day 5 after initiating treatment. (E) Quantification of epidermal thickness of imiquimod-treated $AhR^{+/-}$ and $AhR^{-/-}$ mice treated with either isotype or anti-IL-17A antibody,on day 5 after initiating treatment.(F) Number of neutrophils per cm^2 of skin as determined by FACS analysis of Ly6G+ cells in imiquimod-treated $AhR^{+/-}$ and $AhR^{-/-}$ mice treated with either isotype or anti-IL-17A antibody. (G) mRNA expression of cxcl1 and krt10 in skin of imiquimod-treated $AhR^{+/-}$ and $AhR^{-/-}$ mice treated with either isotype or anti-IL-17A antibody.* $p<0.05$.

Figure S5

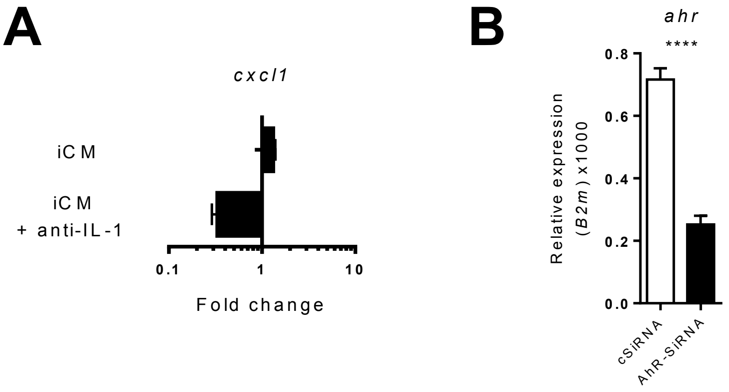

Figure S5 related to Fig.6: (A) *cxcl1* mRNA expression in AhR-deficient keratinocytes stimulated for 24 hours with iCM in the presence or absence of neutralizing anti-IL-1β (10 mg/ml). Data are expressed as fold change over keratinocytes stimulated with in-vitro-reactivated skin cells obtained from naïve wild-type mice. Plots show mean ± SEM. *n* = 2-3 wells per group. (B) *ahr* mRNA expression in human primary keratinocytes, transiently transfected for 48 hours with a non-targeting control SiRNA (cSiRNA, white bars) or in which AhR was transiently silenced (AhR-SiRNA, black bars) and stimulated for further 24 hours with human recombinant IL-1β(10 ng/ml)

Figure S6

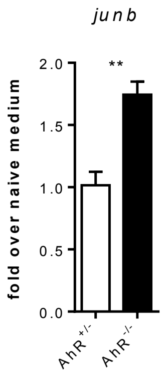

Figure S6 related to Fig.7: (A) *Junb* mRNA expression in $AhR^{+/-}$ (white bars) and $AhR^{-/-}$ (black bars) keratinocytes stimulated for 24 hours with conditioned medium from in-vitro-reactivated skin cells obtained from IMQ-treated wild-type mice. Data expressed as fold change over stimulation with medium conditioned by in-vitro-reactivated skin cells obtained from naïve wild-type mice. Results from one representative experiment of three independent experiments are shown.

Supplemental Experimental Procedures related to Experimental Procedures

Human skin biopsies culture: Psoriasis skin biopsies were quartered and one quarter of each was stored in RNA-later (Life Technologies) at 4^oC until further use. The remaining three quarters were cultured with either 0.1% DMSO (vehicle control), the AhR agonist FICZ (250 nM) or the AhR antagonist CH-2233191 (3 μM) in IMDM (Sigma) containing 2% Pen-Strep-Gln solution (Sigma) and 10% knockout Serum replacement factor (Life Technologies) at 37°C in humidified 5% CO_2/95% air for 16h. Individual skin biopsies from healthy donors were processed in the same ways as those of patients.

Animals: Mice were bred in the NIMR animal facility under specified pathogen free conditions. All animal experiments were done according to institutional guidelines and Home Office regulations. C57Bl/6, B6.CD45.1, *Ahr*$^{-/-}$ (Schmidt et al., 1996) (Jackson Stock 002727 B6;129-$^{AhRtm3.1Bra/}$J), *Rag1.Cre*(McCormack et al., 2003), *Cd11c.Cre* (Jackson stock 008068 B6.Cg-Tg($^{Itgax-cre}$)1-1Reiz/J(Caton et al., 2007), *Ahr*$^{fl/fl}$ (Jackson Stock 006203 B6.129(FVB-Ahr$^{tm3.1Bra/J}$), B6.*Rag1*$^{-/-}$and *Ahr*$^{-/-}$*Rag1*$^{-/-}$ mice were used in this study. Mice were bred in the NIMR animal facility under specified pathogen free conditions. All animal experiments were done according to institutional guidelines and Home Office regulations.

Generation of BM chimeras: BM cells were obtained from femurs and tibias of donor mice. Sub-lethally irradiated Rag1 deficient recipient mice were reconstituted by intravenous injection of $1x10^7$ BM cells.

RNA isolation and Quantitative RT-PCR (qRT-PCR): Total RNA was obtained using the Mirvana Kit (Life Technologies) (human skin), or a Polytron PT 3000 tissue homogenizer

(Kinematika) along with TRIzol (Life technologies) (mouse skin) or RNeasy Mini Plus Kit (Qiagen) (*in vitro* cultured KCs), each according to the manufacturer's instructions. mRNA was reverse transcribed into cDNA and gene expression was assessed by quantitative RT-PCR using Taqman assays (Life technologies) according to the manufacturers' instructions. For each sample, mRNA abundance was normalized to the amount of Human acidic ribosomal protein *(HuPO)* or mouse beta 2-microglobulin (*β2m*). Data analysis was performed using either the ΔΔCt method and results are expressed as agonist or antagonist fold change versus vehicle control (human skin samples), or the ΔCt method and results are expressed as mRNA relative expression to *β2m* x1000 in arbitrary units (mouse skin samples), or the ΔΔCt method and results for each genotype are expressed as fold change over respective conditioned medium from naïve mice (*in vitro* keratinocytes).

cDNA Libraries preparation:

cDNA libraries were prepared using the Illumina TruSeqTM RNA sample preparation kit (Low-Throughput protocol) according to manufacturer's instructions. Briefly, 500µg of total RNA sample was used for poly-A mRNA selection using streptavidin-coated magnetic beads. The resulting RNA sample was subjected to thermal mRNA fragmentation using Elute, Prime, Fragment Mix from the kit. cDNA was synthesized from enriched and fragmented RNA using reverse transcriptase Super-Script II (Life technologies) according to manufacturer's instructions. The cDNA was further converted into double stranded DNA using the reagents supplied in the kit. The resulting dsDNA was subjected end-repair, dA-tailing, ligation of platform-specific adaptors, purification reactions and library amplification (15 cycles).

RNA-sequencing (Accession number GSE47944)

Libraries were barcoded and run 4 per lane on an Illumina HiSeq 2000. Sequencing yielded an average of 35 million paired end reads per library. Read pairs were mapped to the Ensembl Human GRCh37 transcriptome using bowtie with the options "-v 3 -a --best -y -I 0 -X 10000". Any read pair mapping only to one or more transcripts of a gene constituted a unique gene count, and any read pair mapping to transcripts of different genes was discarded. On average 60.5% of reads produced unique mappings. Read counts were normalised by the total unique mapping reads per library.

RNA sequencing differential expression analysis

We tested for the influence of AhR agonist and antagonist whilst controlling for any tissue culture effect. We first estimate the perturbation due to culture (if any), then we estimate the fold change and significance of the AhR treatment beyond that of the culture. Let g_{ijkp} be the normalised count of gene i in tissue $j = \{\text{"L, NL, N}\}\text{"}$ under treatment k = {U = Untreated, C = DMSO, T=AhR Agonist/Antagonist} in patient p, we assume g_{ijkp} is negative binomially distributed (Cameron and Trived, 1998) with mean μ_{ijk} and variance $\mu_{ijk} + \alpha_{ij}\mu_{ijk}^2$, with α_{ij} the dispersion parameter for gene i in tissue j. We consider the null hypothesis a gene's expression is the result of the mean untreated expression and any culture effects: $\log \mu_{ijk} = \mu_{ij} + x_{Ck}\beta_{C_{ij}}$; and the alternative hypothesis that a gene's expression is due to mean untreated expression, culture and treatment effects: $\log \mu_{ijk} = \mu_{ij} + x_{Ck}\beta_{C_{ij}} + x_{Tk}\beta_{T_{ij}}$. Where, μ_{ij} is the mean untreated expression; $\beta_{C_{ij}}$ and $\beta_{T_{ij}}$ are the log fold changes due to culture and treatment respectively; $x_{Ck} = 1$ when $k = C, T$ and 0 otherwise; $x_{Tk} = 1$ when $k = T$ and 0 otherwise. We compute maximum likelihood estimates of parameters $\alpha_{ij}, \mu_{ij}, \beta_{Cij}$ under the null, and similarly estimates of $\alpha_{ij}, \mu_{ij}, \beta_{Cij}, \beta_{Tij}$ under the alternative hypothesis. We perform a likelihood ratio test: if ℓ_N and ℓ_A are the log-

likelihoods of the null and alternative models respectively then the test statistic $-2(\ell_N - \ell_A)$ is assumed χ_1^2 distributed under the null hypothesis. We apply the test to the 15510 genes that have a mean expression of at least 15 reads in 2 of the 12 tissue/treatment combinations. We select the genes for which we reject the null with $p < 0.05$ in any treatment and tissue, we further require the treatment fold changes must be opposing in the relevant tissue (i.e. β_{Tij} must have different signs for agonist and antagonist). This results in 884 AhR-modulated genes (Fig. 1A). We next filtered for consistency of treatment by requiring one of the two treatments to be significant in both lesional (L) and non-lesional(NL), and that the treatment fold changes must be opposing in one of these two tissues. This results in 51 genes of which we select the 41 most likely to be disease relevant by retaining those differentially expressed in an Untreated L v NL comparison with $p < 0.05$ determined by DESeq (Anders and Huber, 2010).

Microarray gene expression analysis (Accession number GSE47607)

Total RNA was used for the Affymetrix sample preparations (Affymetrix) according to the manufacturer's instructions. Three biological repeats were hybridized to GeneChip Mouse Genome 430 2.0 Arrays. Microarray data were RMA normalized using the R package affy (Bolstad *et al.*, 2003) that is a part of the Bioconductor project (http://www.bioconductor.org). Fold changes were calculated as 2|SLR| if SLR>0 and -2|SLR| if SLR<0, where SLR is the average signal log ratio over the biological replicates. The R package RankProd(Hong *et al.*, 2006) was used to identify regulated genes. Genes having the estimated percentage of false positive predictions pfp<0.01 and fold change FC≥1.5 were considered to be differentially expressed. The gene annotations were obtained from the NetAffx database(Liu *et al.*, 2003). Visualization was performed using Eisen's

Treeview(Eisen *et al.*, 1998). The microarray data were also analyzed for psoriasis associated genes using Ingenuity Pathways Analysis (Ingenuity® Systems, www.ingenuity.com).

Keratinocyte cell culture: For murine primary keratinocyte *in vitro* culture, tails and shaved dorsal mouse skin were processed by mechanical removal of subcutanous fat tissue and floated on 0.5% w/v trypsin (Sigma) for 1 h at 37°C. Epidermal sheets were mechanically separated and suspended in high calcium medium (EMEM medium (Lonza) with 8% calcium-chelated fetal calf serum, 2% Pen-Strep-Gln solution (Sigma) (cEMEM) and 1.3mM $CaCl_2$), submitted to gentle vortexing for 1 min, filtered, and cells seeded in fibronectin- and collagen-coated (BD Biosciences and Life Technologies, respectively) 24-well plates at $3x10^5$ cells/ml in medium-calcium medium (cEMEM supplemented with 0.2mM $CaCl_2$). The following day non-adherent cells were washed away with PBS and cells cultured in low-calcium medium (cEMEM supplemented with 0.05 mM $CaCl_2$). Cells were expanded until reaching 70% confluence and then stimulated with conditioned media (see below) or 10ng/ml IL-1β (R&D Systems), with or without 1 μM Tanshinone IIA (Enzo) for 24 hours.

Human primary keratinocyte were isolated from discarded healthy skin as previously described (Laggner et al., 2011) and cultured in KGM medium (KBM medium, supplemented with KGM-bullet kit, both Lonza) for 2-3 passages. For transient silencing of AhR, cells were seeded in 24-well plates at $35x10^4$ cells/ml and cultured until 65% confluent. Cells were then transfected with 33nM ON-TARGETplus SMARTpool siRNA for human AhR or with ON-TARGETplus Non-targeting siRNA (both Thermo Fisher Scientific Biosciences) using Lipofectamine 2000 (Life Technologies) in KBM medium. Forty-eight hours later, medium was replaced with KGM and cells were stimulated with 10ng/ml human IL-1β for further 24 hours. The human keratinocyte HaCaT cell line, in which AhR had been stable silenced (AhR-silenced HaCaT) or which had been transfected with an empty vector (EV-HaCaT)

(Fritsche et al., 2007), were cultured in DMEM medium supplemented with 10% fetal bovine serum, 2% Pen-Strep-Gln solution, 1 mM sodium pyruvate, 800 μg/ml geneticin (Sigma). Cells were seeded in 24-well plates at 5×10^4 cells/ml and stimulated the following day with 10ng/ml human IL-1β for 24 hours.

***In vivo* IL-17a blockade in the imiquimod model of psoriasiform-like skin inflammation**: *Ahr*$^{+/-}$ and *Ahr*$^{-/-}$, treated daily for 5 consecutive days with IMQ, received 500 μg/mouse anti-IL-17a antibody or isotype control (both BioXcell) intraperitoneally on the day the IMQ treatment was started and then on day 3 of treatment.

Supplementary References

Anders, S., and Huber, W. (2010). Differential expression analysis for sequence count data. Genome Biol *11*, R106.
Bolstad, B.M., Irizarry, R.A., Astrand, M., and Speed, T.P. (2003). A comparison of normalization methods for high density oligonucleotide array data based on variance and bias. Bioinformatics *19*, 185-193.
Cameron, A.C., and Trived, P.K. (1998). Regression Analysis of Count Data (Cambridge University Press).
Caton, M.L., Smith-Raska, M.R., and Reizis, B. (2007). Notch-RBP-J signaling controls the homeostasis of CD8- dendritic cells in the spleen. The Journal of experimental medicine *204*, 1653-1664.
Eisen, M.B., Spellman, P.T., Brown, P.O., and Botstein, D. (1998). Cluster analysis and display of genome-wide expression patterns. Proc Natl Acad Sci U S A *95*, 14863-14868.
Fritsche, E., Schafer, C., Calles, C., Bernsmann, T., Bernshausen, T., Wurm, M., Hubenthal, U., Cline, J.E., Hajimiragha, H., Schroeder, P*., et al.* (2007). Lightening up the UV response by identification of the arylhydrocarbon receptor as a cytoplasmatic target for ultraviolet B radiation. Proc Natl Acad Sci U S A *104*, 8851-8856.
Hong, F., Breitling, R., McEntee, C.W., Wittner, B.S., Nemhauser, J.L., and Chory, J. (2006). RankProd: a bioconductor package for detecting differentially expressed genes in meta-analysis. Bioinformatics *22*, 2825-2827.
Laggner, U., Di Meglio, P., Perera, G.K., Hundhausen, C., Lacy, K.E., Ali, N., Smith, C.H., Hayday, A.C., Nickoloff, B.J., and Nestle, F.O. (2011). Identification of a novel proinflammatory human skin-homing Vgamma9Vdelta2 T cell subset with a potential role in psoriasis. J Immunol *187*, 2783-2793.
Liu, G., Loraine, A.E., Shigeta, R., Cline, M., Cheng, J., Valmeekam, V., Sun, S., Kulp, D., and Siani-Rose, M.A. (2003). NetAffx: Affymetrix probesets and annotations. Nucleic Acids Res *31*, 82-86.
McCormack, M.P., Forster, A., Drynan, L., Pannell, R., and Rabbitts, T.H. (2003). The LMO2 T-cell oncogene is activated via chromosomal translocations or retroviral insertion during gene therapy but has no mandatory role in normal T-cell development. Mol Cell Biol *23*, 9003-9013.
Schmidt, J.V., Su, G.H., Reddy, J.K., Simon, M.C., and Bradfield, C.A. (1996). Characterization of a murine Ahr null allele: involvement of the Ah receptor in hepatic growth and development. Proc Natl Acad Sci U S A *93*, 6731-6736.
