# Supplementary material for: Activation of the Aryl Hydrocarbon Receptor Dampens the Severity of Inflammatory Skin Conditions
Source: Immunity. 2014 Jun 19;40(6):989–1001. doi: 10.1016/j.immuni.2014.04.019 (PMC4067745; doi:10.1016/j.immuni.2014.04.019)
Supplement: Document S2. Article plus Supplemental Information [file mmc2.pdf]

CellPress

Activation of the Aryl Hydrocarbon Receptor Dampens the Severity of Inflammatory Skin Conditions

Paola Di Meglio,[1,5] João H. Duarte,[1,5] Helena Ahlfors,[1] Nick D.L. Owens,[2] Ying Li,[1] Federica Villanova,[3] Isabella Tosi,[3] Keiji Hirota,[1,6] Frank O. Nestle,[3] Ulrich Mrowietz,[4] Michael J. Gilchrist,[2] and Brigitta Stockinger[1,*]
[1]Division of Molecular Immunology
[2]Division of Systems Biology
MRC National Institute for Medical Research, Mill Hill, London NW7 1AA, UK
[3]St. John's Institute of Dermatology, King's College London and NIHR Biomedical Research Centre, London SE1 9RT, UK
[4]Psoriasis Center, Department of Dermatology, University Medical Center Schleswig-Holstein, Campus Kiel, 24105 Kiel, Germany
[5]Co-first author
[6]Present address: Department of Experimental Immunology, Immunology Frontier Research Center, Osaka University, 565-0871 Osaka, Japan
*Correspondence: bstocki@nimr.mrc.ac.uk
http://dx.doi.org/10.1016/j.immuni.2014.04.019
This is an open access article under the CC BY license (http://creativecommons.org/licenses/by/3.0/).

SUMMARY

Environmental stimuli are known to contribute to psoriasis pathogenesis and that of other autoimmune diseases, but the mechanisms are largely unknown. Here we show that the aryl hydrocarbon receptor (AhR), a transcription factor that senses environmental stimuli, modulates pathology in psoriasis. AhR-activating ligands reduced inflammation in the lesional skin of psoriasis patients, whereas AhR antagonists increased inflammation. Similarly, AhR signaling via the endogenous ligand FICZ reduced the inflammatory response in the imiquimod-induced model of skin inflammation and AhR-deficient mice exhibited a substantial exacerbation of the disease, compared to AhR-sufficient controls. Nonhematopoietic cells, in particular keratinocytes, were responsible for this hyperinflammatory response, which involved upregulation of AP-1 family members of transcription factors. Thus, our data suggest a critical role for AhR in the regulation of inflammatory responses and open the possibility for novel therapeutic strategies in chronic inflammatory disorders.

INTRODUCTION

The skin is the organ most exposed to environmental insults, and its complex cellular network constitutes an immunological barrier that is crucial for the maintenance of homeostasis (Di Meglio et al., 2011). It is therefore likely that inflammatory disorders of the skin involve environmental factors. One such disorder is plaque-type psoriasis, a disease with complex etiopathogenesis, characterized by epidermal hyperproliferation and prominent immune infiltrates (Nestle et al., 2009). Crosstalk between innate, adaptive, and epithelial or stromal cells, such as keratinocytes and fibroblasts, underpins the disease pathology (Lowes et al., 2013). A total of 36 disease-associated loci have been identified as contributing to psoriasis (Tsoi et al., 2012). Environmental risk factors, on the other hand, remain less well defined on a mechanistic basis (Tagami, 1997). Although no mouse model can fully recapitulate the development and features of psoriasis (Gudjonsson et al., 2007), topical application of the imiquimod (IMQ)-containing cream Aldara induces a psoriasiform skin inflammation, which exhibits most of the crucial traits (Swindell et al., 2011) including acanthosis, parakeratosis, neutrophil recruitment, and involvement of the IL-23-IL-17-IL-22 pathway (van der Fits et al., 2009), and is thus increasingly used to dissect the mechanisms of psoriasis pathogenesis.

In order to investigate the potential influence of environmental factors on inflammatory skin disease, we have focused on the ligand-dependent transcription factor aryl hydrocarbon receptor (AhR), which responds to environmental stimuli and plays an important role in the maintenance of intestinal homeostasis. Work from our lab and others has shown that AhR-deficient mice have an inherent weakness of the gut barrier (Kiss et al., 2011; Lee et al., 2012; Li et al., 2011; Qiu et al., 2012). The wide expression of AhR in several cell types of the skin suggests a role for AhR signaling also at this barrier organ. The AhR is a member of the bHLH-PAS family of transcription factors best known for mediating the toxic effects of environmental contaminants such as TCDD (dioxin) and a range of other xenobiotic substances. However, its evolutionary conservation from invertebrate species onward points to a physiological role that does not involve xenobiotic stimuli (Hahn et al., 2006; McMillan and Bradfield, 2007). Endogenous ligands of AhR are found as indoles and flavonoids either of dietary origin (e.g., indolo[3,2-b] carbazole, ICZ) (Bjeldanes et al., 1991; Gillner et al., 1985) or, like the high-affinity ligand 6-formylindolo[3,2-b]carbazole (FICZ), derived from tryptophan metabolism via UV or visible light exposure (Rannug and Fritsche, 2006), which has been found to be physiologically relevant in human skin (Katiyar et al., 2000; Rannug and Fritsche, 2006).

Combining the analysis of psoriasis patient skin biopsies with that of a mouse model of psoriasiform inflammation, we showed that AhR signaling in nonhematopoietic cells plays a central role in preventing excessive skin inflammation.

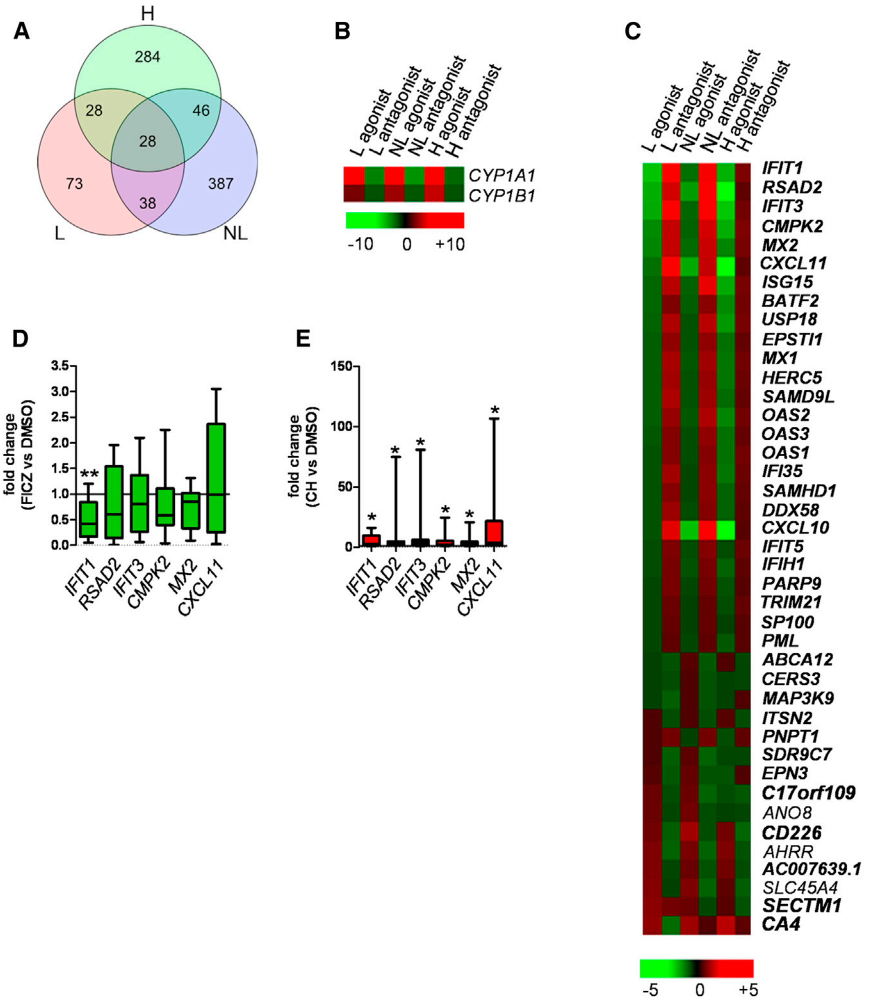

Figure 1. AhR Ligation in Human Skin Biopsies Modulates Psoriasis-Relevant Genes

Lesional (L) and nonlesional (NL) skin biopsies from eight psoriasis patients were quartered: one quarter of each was used as baseline and the remaining three quarters were cultured with either vehicle control, the AhR agonist FICZ, or the AhR antagonist CH-223191 for 16 hr. Whole-skin biopsies from five healthy donors (H) were processed in the same way. All samples were subjected to RNA sequencing.

(A) Venn diagram showing genes significantly regulated by either FICZ or CH-223191 in at least one out of the three tissue types analyzed (L, NL, H).

(B) Heat map of known AhR-target genes modulated by FICZ and CH-223191. Color indicates mean fold change, with green representing decreased and red increased gene expression.

(C) Heat map of genes belonging to the "psoriasis transcriptome" (upregulated genes shown in bold) and modulated by FICZ or CH-223191. Genes are sorted by decreasing fold change for agonist effect on L skin.

(D) qPCR validation for six top modulated genes downregulated in L skin by agonist-induced AhR activation. Box and whiskers denoting minimum and maximum values are shown. Wilcoxon signed rank test (for *CMPK2*) or paired t test (all other genes) was performed.

(E) qPCR validation of six top modulated genes upregulated in NL skin by antagonist-induced AhR inhibition. Box and whiskers denoting minimum and maximum values are shown. Wilcoxon signed rank test was performed.

RESULTS

AhR Ligation in Human Skin Biopsies Modulates Psoriasis-Relevant Genes

In order to address whether the AhR pathway has a role in human skin pathology, we investigated whether AhR activation via the agonist FICZ or inhibition via the antagonist CH-223191 (Kim et al., 2006) would cause transcriptional changes in psoriasis-related genes. Full-thickness skin biopsies were obtained from lesional (L) and uninvolved nonlesional (NL) skin of eight psoriatic patients not receiving any systemic treatment and from healthy human subjects (N) serving as control (Table S1 available online). The biopsies were quartered and one segment was reserved for RNA sequencing without any treatment. The remaining three quarters were cultured with DMSO (vehicle control), FICZ, or CH-223191 for 16 hr. After treatment all samples were subjected to RNA sequencing. Analysis of the untreated L and NL psoriasis skin samples provided us with genes differentially regulated in the two tissue types, or the "psoriasis transcriptome." The top 25 most significantly regulated genes (Table S2) were in keeping with published data sets (Gudjonsson et al., 2010; Tian et al., 2012) with several members of the S100 protein family significantly upregulated in L versus NL skin. Next, we identified the transcriptional changes induced by exposure to AhR agonist or antagonist. A flowchart describing step by step our filtering criteria is shown in Figure S1. We identified 884 AhR-modulated genes, defined as genes significantly regulated by either the agonist or the antagonist in at least one out of the three tissue types analyzed (L, NL, H) (Figure 1A). As expected, expression of *CYP1A* and *CYP1B1*, well-characterized AhR-target genes, were found to be highly upregulated in the presence of agonist and downregulated by the antagonist in all three tissue types (Figure 1B). The list of AhR-modulated genes was reduced to 41 genes belonging to the "psoriasis transcriptome," which were mainly upregulated in untreated L skin (Figure 1C, psoriasis-upregulated genes are shown in bold, and Table S3). Out of these, 29 (70%) were reduced after FICZ-induced AhR activation. This effect was most prominent in L skin, as confirmed by qRT-PCR showing a downward trend for the top five regulated genes (*IFIT*, *RSAD2*, *IFIT3*, *CMPK2*, *MX2*), which reached statistical significance for IFIT1 (Figure 1D). Conversely, treatment with AhR antagonist was able to increase expression of these genes in NL skin, with statistically significant fold change increase for all genes validated by qRT-PCR (Figure 1E). Ingenuity pathway analysis showed that 26 out of 41 psoriasis-relevant genes modulated by AhR belong to the type I and II IFN pathway,

CellPress

which is known to be upregulated in psoriasis (Figure S1). Thus, AhR appears to play a critical role in modulating the severity of psoriasis. In order to study the influence of AhR in more detail, we employed the mouse model of IMQ-induced psoriasiform inflammation.

AhR-Deficient Mice Develop Exacerbated IMQ-Induced Psoriasiform Skin Inflammation

Treatment of AhR-deficient (*Ahr*$^{-/-}$) and AhR-heterozygous littermate control (*Ahr*$^{+/-}$) mice with IMQ over a 5-day period resulted in scaling and parakeratosis of the Stratum corneum and epidermal acanthosis and widespread inflammatory infiltrates, as seen by visual inspection (Figure S2A) and in H&E-stained skin sections (Figure 2A). Untreated skin of *Ahr*$^{-/-}$ and *Ahr*$^{+/-}$ littermate controls was histologically indistinguishable, but upon treatment the thickening of both epidermis and Stratum corneum was significantly increased in *Ahr*$^{-/-}$ mice (Figure 2B). Nevertheless, the exacerbated skin pathology elicited by IMQ treatment in the absence of AhR signaling abated after termination of treatment. Quantitative RT-PCR analysis of inflamed skin from *Ahr*$^{-/-}$ mice revealed statistically significant increased expression of growth factors and chemokines involved in neutrophil attraction (*Csf2*, *Csf3*, *Cxcl1*, *Cxcl5*) and of antimicrobial peptides typically present in psoriasis lesions (*S100a7a*, *S100a8*), as well as reduced expression of the keratinocyte differentiation marker *Krt10* (Figure 2C). Moreover, mRNA expression of a number of proinflammatory cytokines, including *Il17a*, *Il17c*, *Il23*, *Il22*, and *Il1b*, which are crucially involved in psoriatic skin inflammation (Di Cesare et al., 2009; van der Fits et al., 2009), was significantly increased in the skin of *Ahr*-deficient mice (Figure 2D). Upregulation of *Il1b* mRNA in *Ahr*$^{-/-}$ mice preceded that of IL-17 (Figure S2B) and remained increased at protein level on day 5 (Figure S2C). Although there was substantial infiltration of T cells producing IL-17 cell type cytokines, the majority of which were $\gamma\delta$ T cells (Figure S2D) as previously reported (Pantelyushin et al., 2012), absolute numbers of IL-17- and IL-22-producing CD4 and $\gamma\delta$ T cells did not differ between *Ahr*$^{+/-}$ and *Ahr*$^{-/-}$ mice (Figure 2E). In agreement with our earlier finding (Martin et al., 2009), AhR-deficient $\gamma\delta$ T cells appear to make more IL-17 on a per cell basis, thus accounting for the increase in IL-17 observed in the skin of *Ahr*$^{-/-}$ mice (Figures S2E and S2F). In line with the increase in neutrophil-recruiting chemokines, there was significantly more neutrophil infiltration into the skin in *Ahr*$^{-/-}$ mice (Figure 2F), whereas the number of both macrophages and dendritic cells (DCs) did not differ between the two groups (Figure S2G), and we did not observe substantial infiltration of IL-17-producing CD8 T cells or innate lymphoid cells (data not shown). Thus, absence of AhR signaling led to heightened inflammation and exacerbated skin pathology. This phenotype was not restricted to the psoriasisform inflammation induced by IMQ, but also extended to a model of delayed-type hypersensitivity (DTH) skin reactions in *Ahr*-deficient mice, which showed enhanced neutrophil infiltration and increased inflammatory chemokine expression (data not shown).

AhR Activation by FICZ Ameliorates IMQ-Induced Psoriasiform Skin Inflammation

Next, we asked whether deliberate triggering of the AhR pathway would have a beneficial effect on skin pathology as seen in human psoriatic skin. To this end, wild-type mice received intraperitoneal injections of either FICZ or olive oil (vehicle control) daily during the course of IMQ treatment. FICZ administration upregulated expression of *Cyp1a1* mRNA in the skin, as compared to mice receiving vehicle only (Figure 3A), and resulted in attenuated psoriasiform skin inflammation, with milder parakeratosis and cell infiltration (Figure 3B), statistically significant reduction in epidermal and scale thickness (Figure 3C), and reduced expression of proinflammatory mediators (Figures 3D and S3). Thus, activation of the AhR pathway in vivo results in amelioration of psoriasiform skin pathology.

AhR Deficiency in Nonhematopoietic Cells Causes Exacerbated Skin Inflammation

In order to identify the AhR-expressing cell type responsible for the hyperinflammatory skin response seen in *Ahr*$^{-/-}$ mice, we generated mice with conditional deletion of AhR in distinct immune cell subsets in the skin. *Ahr*$^{fl/-}$ *Cd11c*.Cre or *Ahr*$^{fl/-}$ *Rag1*.Cre mice, in which AhR is deleted in DCs and some macrophage subsets or in T and B cells, respectively, were treated with IMQ in order to address the contribution of these cells to the exacerbated skin inflammation seen in complete AhR deficiency. qPCR analysis confirmed the deletion of AhR in DCs or in T cells of these mice (Figures S4A and S4C). Lack of AhR in DCs or macrophages did not result in increased inflammation above that observed in control *Ahr*$^{fl/+}$ *Cd11c*.Cre mice (Figures 4A–4D and S4B). Lack of AhR in T and B cells resulted in increased skin acanthosis and reduced keratinocyte differentiation, but no difference in skin scaling, expression of the majority of inflammatory mediators, or number of neutrophils when compared to control mice (Figures 4E–4H and S4D). These observations ruled out a role for DCs or macrophages in driving the exacerbated skin inflammation seen in *Ahr*$^{-/-}$ mice, but left open the possibility that activation of the AhR pathway in T or B lymphocytes is important for skin homeostasis.

Next, we addressed the contribution of *Ahr* deficiency in nonhematopoietic skin cells such as keratinocytes and fibroblasts. We generated bone marrow (BM) chimeras in which hematopoietic cells were of wild-type origin, whereas the nonhematopoietic compartment was either *Ahr*$^{-/-}$ (rAhR$^{-/-}$) or wild-type (rAhR$^{+/+}$) by reconstituting *Ahr*$^{-/-}$*Rag1*$^{-/-}$ and control *Ahr*$^{+/+}$ *Rag1*$^{-/-}$ hosts with BM from *Ahr* wild-type donors. AhR deficiency in the nonhematopoietic compartment recapitulated the hyperinflammatory phenotype of full AhR$^{-/-}$ mice with exacerbated epidermal pathology (Figures 5A and 5B), increased neutrophil recruitment (Figure 5C), overexpression of inflammatory markers, and reduced keratinocyte differentiation (Figure 5D). In contrast, expression of both IL-17 and IL-22 was not different in the two experimental groups, making it unlikely that these cytokines are responsible for the hyperinflammatory skin response of *Ahr*$^{-/-}$ mice. In line with this, treatment of *Ahr*$^{-/-}$ mice with neutralizing antibody to IL-17A did not improve their exaggerated response (Figures S4E–S4G).

BM chimeras in which the nonhematopoietic cells were of wild-type origin, whereas the hematopoietic compartment was either *Ahr*$^{+/-}$ (dAhR$^{+/-}$) or *Ahr* deficient (dAhR$^{-/-}$), did not show increased inflammation above that observed in control mice (Figures 5E–5G). Disease severity was not increased in *Ahr*$^{-/-}$*Rag1*$^{-/-}$ versus *Ahr*$^{+/-}$ *Rag1*$^{-/-}$ (data not shown),

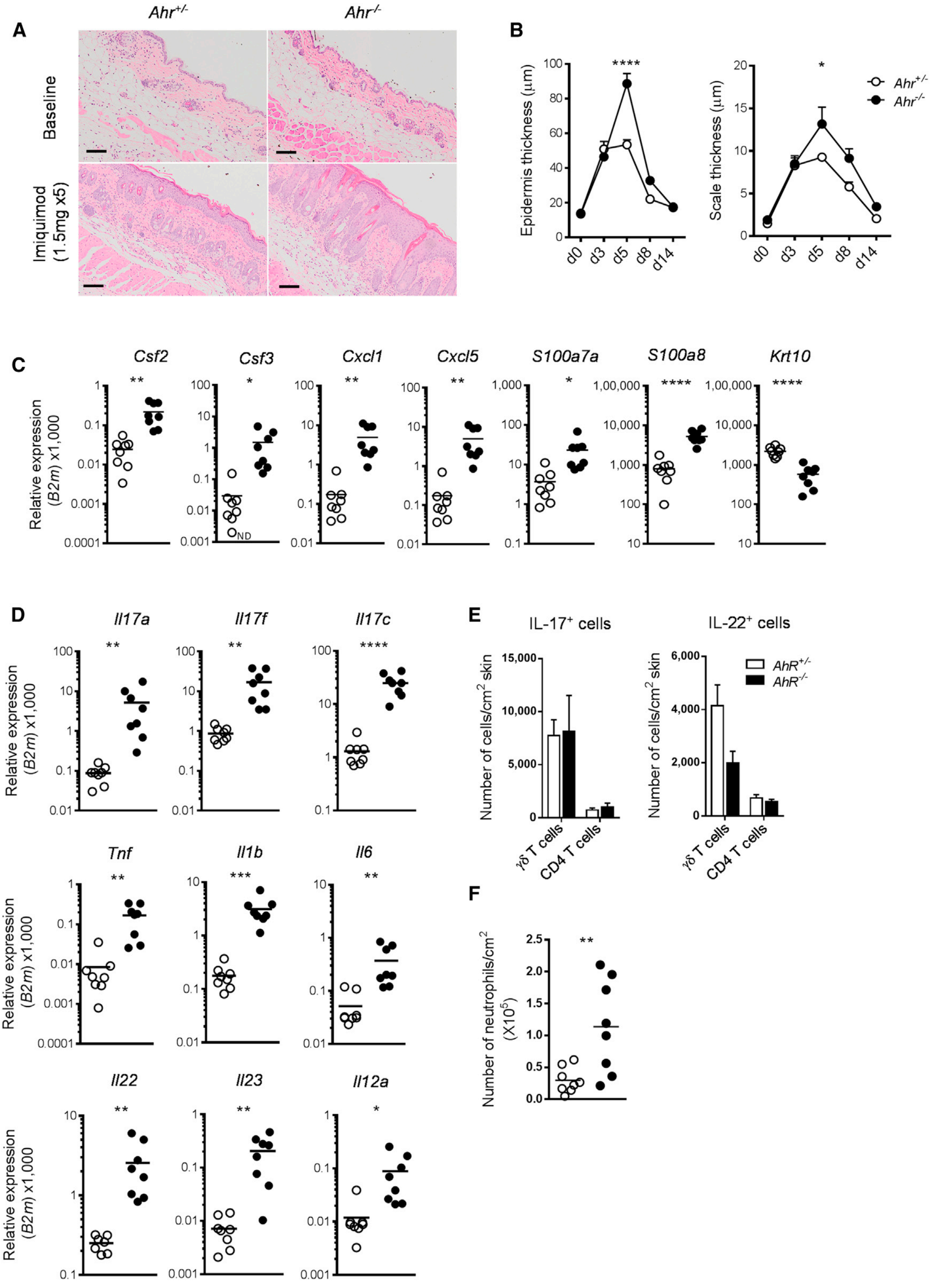

Figure 2. Exacerbated Skin Inflammation in *Ahr*-Deficient Mice

(A) Representative images of H&E staining of skin sections from untreated (baseline) and IMQ-treated *Ahr*$^{+/-}$ (open circles) and *Ahr*$^{-/-}$ (filled circles) mice at day 5 (scale bars represent 100 μm).

(B) Quantification of epidermal (left) and scale thickness (right) of *Ahr*$^{+/-}$ (open circles) and *Ahr*$^{-/-}$ (filled circles) at different time points after initiation of IMQ treatment.

(legend continued on next page)

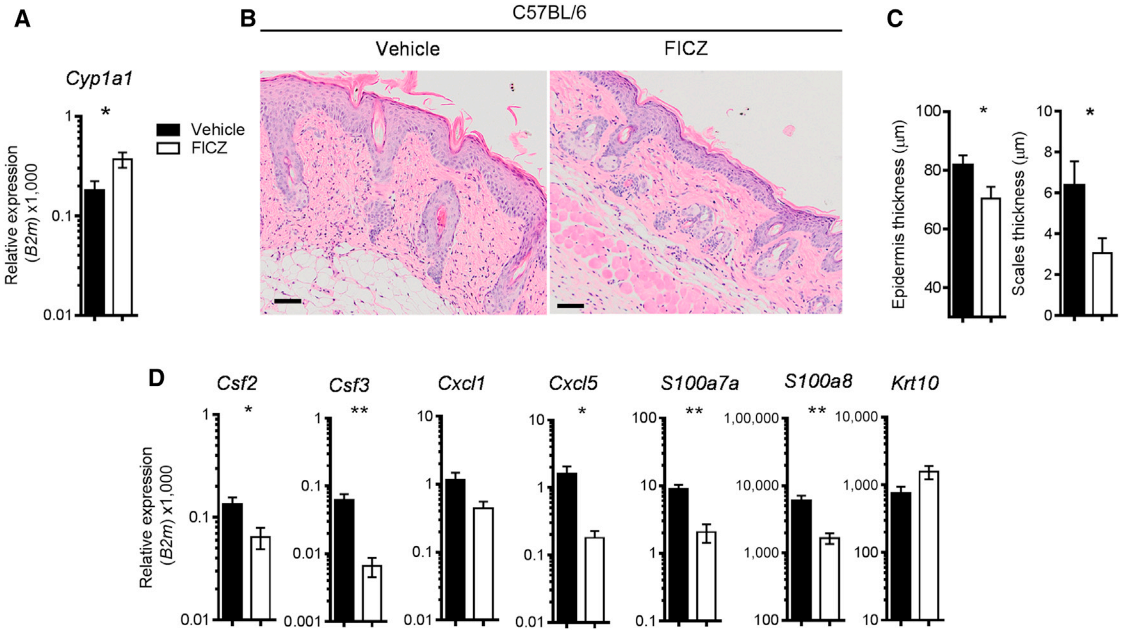

Figure 3. Skin AhR Activation by FICZ Ameliorates IMQ-Induced Psoriasis-like Skin Inflammation

(A) *Cyp1a1* expression in skin of IMQ-treated C57BL/6 mice receiving vehicle (black bars) or FICZ (white bars) i.p. for 6 days.

(B) H&E staining of skin sections (day 6) from IMQ-treated C57BL/6 mice receiving vehicle or FICZ.

(C) Quantification of epidermal (left) and scale thickness (right) at day 6 of vehicle-treated (black bars) or FICZ-treated (white bars) mice.

(D) mRNA expression of psoriasis-relevant genes in whole skin from IMQ-treated C57BL/6 mice receiving vehicle (black bars) or FICZ (white bars).

Plots show mean ± SEM; n = 5 mice per group. Results from one representative experiment of three independent experiments are shown. *p < 0.05, **p < 0.01.

emphasizing that the cross-talk between adaptive immune cells and epidermal cells is essential for the exacerbated pathology observed in *Ahr*-deficient mice. Therefore, AhR deficiency in the nonhematopoietic skin compartment is necessary and sufficient for the development of an exacerbated psoriasiform skin response in the presence of a fully functioning adaptive immune system.

AhR-Deficient Keratinocytes Show Exacerbated Response to Proinflammatory Cytokines

Our results showed that physiological AhR activation can ameliorate the inflammatory program in nonhematopoietic skin cells. In order to discriminate between the inflammatory response of epithelial and stromal cells, we assessed the response of *Ahr*-sufficient or -deficient keratinocytes to the immune activators produced in the early phase of IMQ-induced skin inflammation. We therefore stimulated keratinocytes from $Ahr^{+/-}$ and $Ahr^{-/-}$ mice in vitro with conditioned medium (CM) from in-vitro-reactivated skin cells obtained from either naive (nCM) or 2-day IMQ-treated (iCM) wild-type mice. $Ahr^{-/-}$ keratinocytes responded to iCM by significantly overexpressing proinflammatory cytokine and chemokine mRNA as compared to heterozygous controls (Figure 6A). $Ahr^{-/-}$ fibroblasts also displayed an increased response as compared to their $Ahr^{+/-}$ counterparts, although to a lesser extent (data not shown). IL-1β is one of the mediators in the early phase of the IMQ-induced skin inflammation model (Walter et al., 2013), and this cytokine was overrepresented in the conditioned medium obtained from wild-type IMQ-treated mice, far exceeding other cytokines tested such as TNF, IL-23, and IL-17A (data not shown). Indeed, recombinant IL-1β could replace iCM, causing comparable upregulation of inflammatory markers on keratinocytes (Figure 6B), whereas neutralizing IL-1β abrogated the proinflammatory response to the skin cell conditioned medium (Figure S5A). In addition, we also found increased expression of *Il1r1* in ex vivo $Ahr^{-/-}$ keratinocytes (Figure 6C).

Furthermore, similar hyperresponsiveness was found in human keratinocytes upon knockdown of *AHR* expression. Thus, normal primary keratinocytes in which *AHR* expression was reduced by *AHR*-SiRNA showed overexpression of proinflammatory mediators compared to keratinocytes transfected with

(C and D) mRNA expression of proinflammatory mediators and keratinocyte differentiation marker in whole skin from $Ahr^{+/-}$ (open circles) and $Ahr^{-/-}$ (filled circles) mice at day 5.

(E) Number of CD4 and γδ T cells expressing IL-17 (left) and IL-22 (right) per cm^2 skin of IMQ-treated $Ahr^{+/-}$ (white bars) and $Ahr^{-/-}$ (black bars) mice obtained by intracellular cytokine staining.

(F) Number of neutrophils ($\times 10^5$) per cm^2 of skin.

Plots show mean ± SEM, n = 3–5 mice per group or mean and values of individual mice, n = 8 mice per group. Results from one representative experiment of two independent experiments are shown. *p < 0.05, **p < 0.01, ***p < 0.001, and ****p < 0.0001.

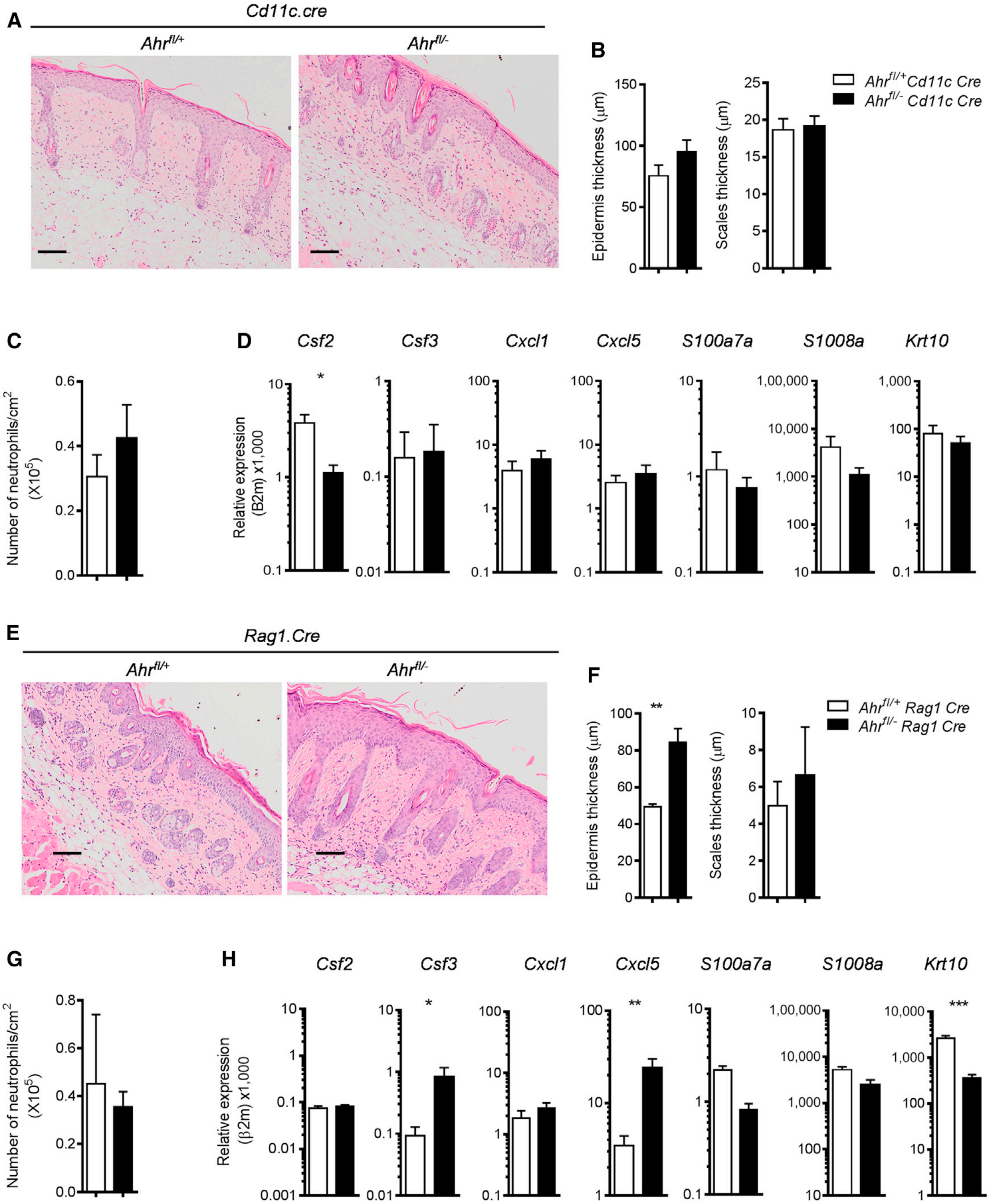

Figure 4. AhR Deficiency in Dendritic Cells or in T and B Cells Does Not Recapitulate the Phenotype of *Ahr*$^{-/-}$ Mice

(A) Representative images of H&E staining of skin sections from imiquimod-treated *Ahr*$^{fl/+}$ or *Ahr*$^{fl/-}$ *Cd11c.Cre* mice at day 5 (scale bars represent 100 μm).
(B) Quantification of epidermal (left) and scale thickness (right) at day 5.
(C) Number of neutrophils per cm^2 of skin as determined by FACS analysis of Ly6G$^+$ cells.
(D) mRNA expression of psoriasis-relevant genes in whole skin from *Ahr*$^{fl/+}$ (white bars) or *Ahr*$^{fl/-}$ *Cd11c.Cre* mice (black bars) at day 5.
(E) Representative images of H&E staining of skin sections from imiquimod-treated *Ahr*$^{fl/+}$ or *Ahr*$^{fl/-}$ *Rag1.Cre* mice (scale bars represent 100 μm).
(F) Quantification of epidermal (left) and scale thickness (right) at day 5.
(G) Number of neutrophils per cm^2 of skin as determined by FACS analysis of Ly6G$^+$ cells.
(H) mRNA expression of psoriasis -relevant genes in whole skin from *Ahr*$^{fl/+}$ (white bars) or *Ahr*$^{fl/-}$ *Rag1.Cre* mice (black bars) at day 5.
Plots show mean + SEM; n = 4–6 mice per group. Results from one representative experiment of two independent experiments per mouse strain are shown. *$p < 0.05$, **$p < 0.01$, and ***$p < 0.001$.

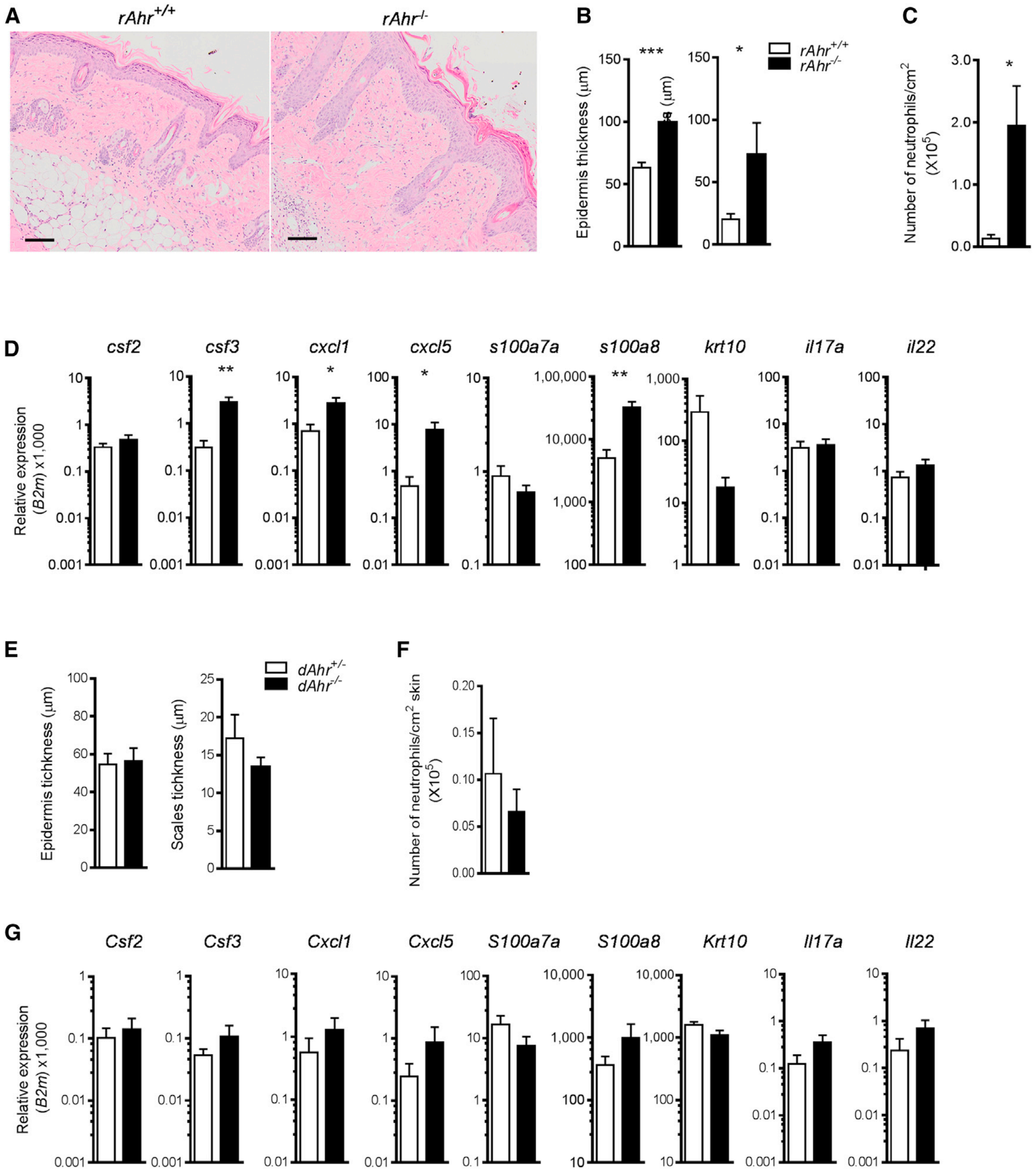

Figure 5. Lack of AhR in Nonhematopoietic Cells Recapitulates Exacerbated Skin Inflammation of $Ahr^{-/-}$ Mice

(A) Representative images of H&E staining of skin sections from IMQ-treated $Ahr^{+/+} \rightarrow Ahr^{+/+}Rag1^{-/-}$ (rAhR$^{+/+}$) or $Ahr^{+/+} \rightarrow Ahr^{-/-}Rag1^{-/-}$ (rAhR$^{-/-}$) BM chimeras (scale bars represent 100 μm).

(B) Quantification of epidermal (left) and scale thickness (right) of rAhR$^{+/+}$ (white bars) or rAhR$^{-/-}$ (black bars) chimeras at day 5.

(C) Number of neutrophils per cm^2 of skin.

(D) mRNA expression of psoriasis-relevant genes in whole skin from rAhR$^{+/+}$ (white bars) or rAhR$^{-/-}$ (black bars) chimeras at day 5.

(E) Quantification of epidermal (left) and scale thickness (right) of $Ahr^{+/-} \rightarrow Ahr^{+/+}Rag1^{-/-}$ (dAhR$^{+/-}$, white bars) or $Ahr^{-/-} \rightarrow Ahr^{+/+}Rag1^{-/-}$ (dAhR$^{-/-}$, black bars) chimeras at day 5.

(F) Number of neutrophils per cm^2 of skin.

(G) mRNA expression of psoriasis-relevant genes in whole skin from dAhR$^{+/-}$ (white bars) or dAhR$^{-/-}$ (black bars) chimeras at day 5.

Plots show mean + SEM; n = 5–7 mice per group. Results from one representative experiment of two independent experiments per each set of chimeras are shown. *$p < 0.05$, **$p < 0.01$, and ***$p < 0.001$.

a nontargeting SiRNA (cSiRNA) (Figures 6D and S5B). Similar results were also obtained in the spontaneously transformed keratinocyte cell line HaCaT, in which *AHR* had been stably silenced (*AHR*-silenced HaCaT), when compared to cells transfected with an empty vector (EV-HaCaT) (Figure 6E; Fritsche et al., 2007).

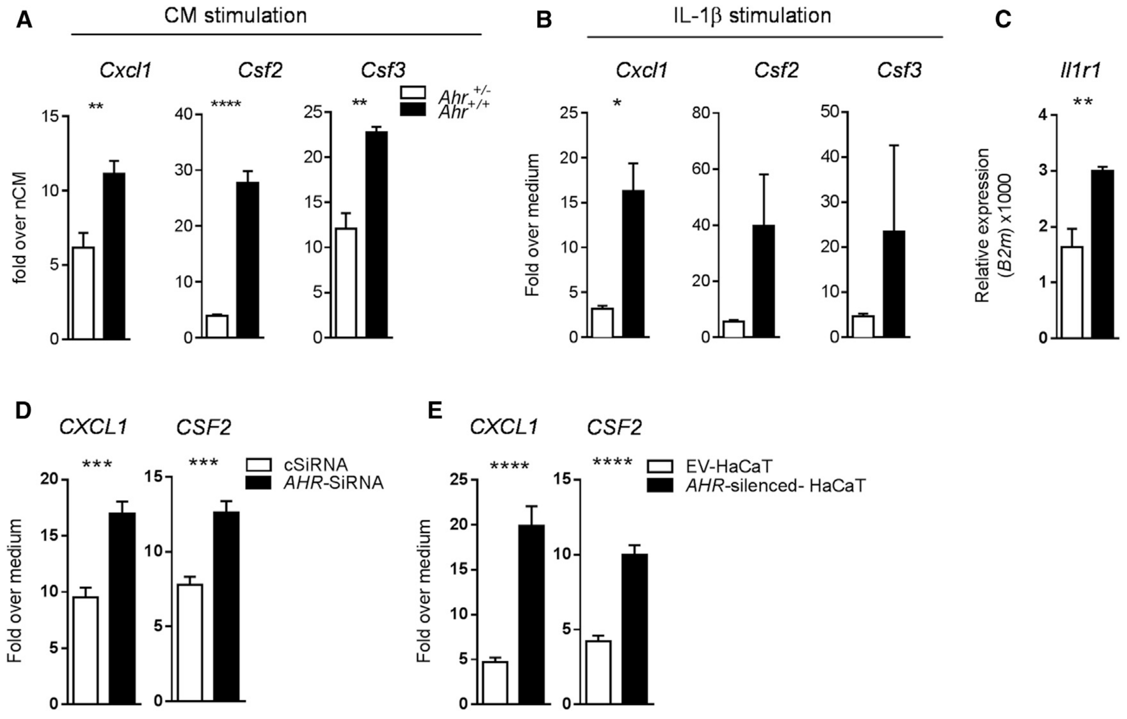

Figure 6. *Ahr*-Deficient Murine and Human Keratinocytes Show Exacerbated Response to Inflammatory Stimuli

(A) mRNA expression of proinflammatory mediators in $Ahr^{+/-}$ (white bars) and $Ahr^{-/-}$ (black bars) murine keratinocytes stimulated for 24 hr with conditioned medium nCM and iCM. Data are expressed as fold change over stimulation with iCM over nCM medium.

(B) mRNA expression of proinflammatory mediators in $Ahr^{+/-}$ (white bars) and $Ahr^{-/-}$ (black bars) murine keratinocytes stimulated for 24 hr with recombinant IL-1β (10 ng/ml). Data expressed as fold change over medium control.

(C) *Il1r1* mRNA expression in unstimulated murine primary keratinocytes from $Ahr^{+/-}$ (white bars) and $Ahr^{-/-}$ (black bars) mice.

(D) mRNA expression of proinflammatory mediators in human primary keratinocytes, transiently transfected for 48 hr with a nontargeting control SiRNA (cSiRNA, white bars) or in which AhR was transiently silenced (AhR-SiRNA, black bars), and stimulated for further 24 hr with human recombinant IL-1β (10 ng/ml).

(E) mRNA expression of proinflammatory mediators in human keratinocytes HaCaT cell lines, stable transfected with an empty vector (EV-HaCaT, white bars), or in which AhR had been stable silenced (AhR-silenced HaCaT, black bars), and stimulated for 24 hr with human recombinant IL-1β. Data expressed as fold change over medium control.

Results from one representative experiment of two or three independent experiments are shown. Plots show mean ± SEM; n = 3–6 wells per group. *p < 0.05, **p < 0.01, and ****p < 0.0001.

Taken together, these results show that nonhematopoietic skin cells, particularly keratinocytes, require AhR to control expression of inflammatory mediators in response to inflammatory stimuli, such as IL-1β. Therefore we conclude that AhR deficiency in both epithelial and stromal cells results in a cell-intrinsic overreaction to inflammatory stimuli, leading to exacerbated skin pathology in vivo.

AhR Modulates *JunB* Expression in Keratinocytes

The AhR is thought to be involved in extensive cross-talk with other transcription factors and multiple signaling pathways, which makes the systematic analysis of physiological interactions a challenging task. To gain more insights about how AhR modulates keratinocyte activation, we focused on the early phase of the IMQ treatment and performed microarray analysis of whole skin from $Ahr^{+/-}$ or $Ahr^{-/-}$ mice treated for 2 days with IMQ. By using Ingenuity pathway analysis, we identified a number of psoriasis-annotated transcription factors regulated by the IMQ treatment and found an overrepresentation of the activator protein-1 (AP-1) family of transcription factors (Figure 7A). AP-1 regulates a range of biological mechanisms, including keratinocyte differentiation and proliferation (Bata-Csorgo and Szell, 2012) and epithelial cell immune activation (Wang et al., 2013). Also, the AP-1 family member JunB, whose activity is transcriptionally regulated (Karin et al., 1997), is increased in psoriasis and localizes to keratinocyte nuclei within the hyperplastic epidermis of psoriasis lesions (Haider et al., 2006; Swindell et al., 2013). We found the expression of *Junb* significantly increased in the skin of $Ahr^{-/-}$ mice as compared to $Ahr^{+/-}$ mice in the early phase (day 2) of IMQ-induced inflammation (Figure 7B), whereas whole skin isolated from FICZ-treated wild-type mice showed reduced *Junb* levels (Figure 7C), suggesting that AhR can control the expression of this AP-1 family member. Moreover, isolated keratinocytes from $Ahr^{-/-}$ mice significantly upregulated JunB protein (Figure 7D) and mRNA (Figure S6) in response to IL-1β and iCM. Finally, inhibition of the AP-1 pathway with the inhibitor Tanshinone IIA (Tseng et al., 2013) resulted in decreased expression of proinflammatory genes in $Ahr^{-/-}$ keratinocytes (Figure 7E). These findings show that AhR controls the expression of other transcription factors responsible for establishing an inflammatory transcriptional program in keratinocytes, e.g., AP-1, and thus constitutes

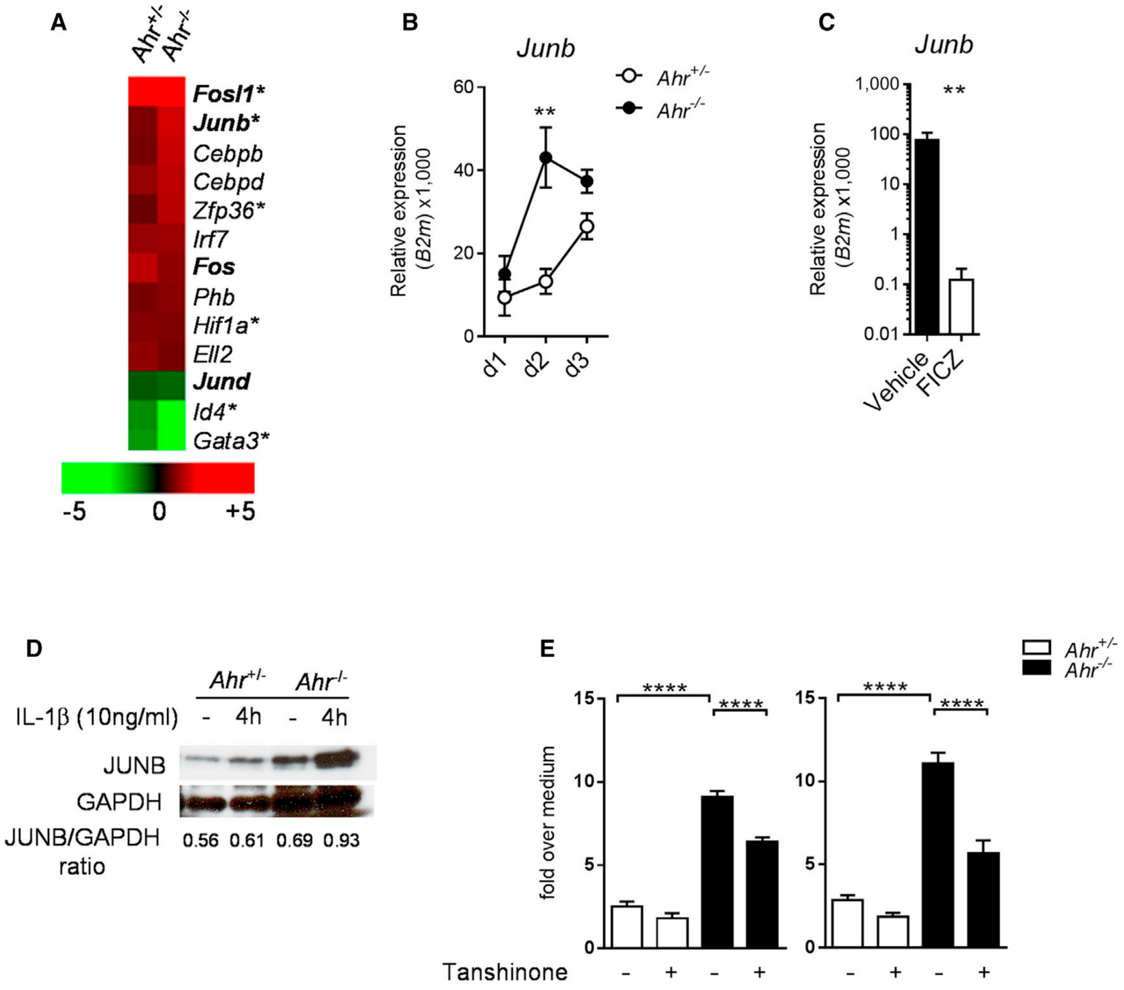

Figure 7. *Ahr*-Deficient Keratinocytes Display Increased Levels of JunB

(A) Heat map visualization of IMQ-regulated, psoriasis-annotated transcription factors in whole skin of $Ahr^{+/-}$ and $Ahr^{-/-}$ mice on day 2 of IMQ treatment. Green color indicates decreased and red color increased gene expression, expressed as mean fold change in IMQ-treated as compared to corresponding untreated mice. AP-1 family transcription factors are marked in bold. Asterisks indicate genes differentially expressed in $Ahr^{-/-}$ versus $Ahr^{+/-}$ skin at day 2 of IMQ treatment.

(B) Time course of *Junb* mRNA expression in whole skin from $Ahr^{+/-}$ (open circles) and $Ahr^{-/-}$ (filled circles) mice.

(C) *Junb* mRNA expression in whole skin from IMQ-treated C57BL/6 mice receiving vehicle (black bars) or FICZ (white bars) at day 6.

(D) Immunoblot showing JunB protein levels in $Ahr^{+/-}$ (white bars) and $Ahr^{-/-}$ (black bars) murine keratinocytes stimulated for 4 hr with recombinant IL-1β (10 ng/ml). Values denotes JunB/GAPDH densitometry ratio.

(E) mRNA expression of *Csf2* and *Csf3* in murine keratinocytes stimulated for 24 hr with recombinant IL-1β (10 ng/ml) with or without Tanshinone (1 μM). Data expressed as fold change over medium control.

Plots show mean ± SEM; n = 3–6 wells or mice per group. Results from one representative experiment of two independent experiments are shown. **$p < 0.01$, ***$p < 0.001$, and ****$p < 0.0001$.

a crucial regulator in the development of skin inflammatory processes.

DISCUSSION

The contribution of environmental factors to the pathogenesis of inflammatory disorders is well known, but the nature and mode of action of such stimuli remains ill defined. We show here that environmental signals transmitted via AhR dampen the inflammatory response in both mouse and human skin. Lack of *Ahr* causes hyperinflammation, whereas deliberate AhR activation with the endogenous ligand FICZ ameliorates the inflammatory profile in both human psoriasis samples and the mouse model of psoriasiform skin inflammation. Our data emphasize the cross-talk between cells of the immune system and nonhematopoetic cells during inflammation, and it is now widely recognized that such interactions crucially underpin both the homeostasis of the skin environment and its dysregulation in diseases such as psoriasis (Di Meglio et al., 2011; Lowes et al., 2013). The focus for therapeutic intervention in psoriasis is currently on modulating inflammatory immune parameters such as IL-17, IL-12, IL-23, or TNF (Lowes et al., 2013), which are the immune drivers of skin pathology in both human disease and the mouse model. In agreement with the literature (Pantelyushin et al., 2012; Van

Belle et al., 2012; van der Fits et al., 2009), we found many parameters linked to the IL-17 program highly upregulated in IMQ-induced skin inflammation and even further exacerbated in *Ahr*$^{-/-}$ mice. However, our data show that immune cells are not the main cause for the hyperreactivity observed in *Ahr*-deficient mice. Instead, the response of nonhematopoietic skin cells, primarily keratinocytes but also skin fibroblasts, to inflammatory stimuli was severely dysregulated in the absence of AhR. However, in vivo blockade of this cytokine was not sufficient to dampen the exaggerated skin response of *Ahr*-deficient mice (data not shown), suggesting that multiple inflammatory pathways are involved.

It was important to delineate contributions by different cell types because it was suggested, for instance, that the epidermal TCR-$\gamma\delta$ population, which interacts with keratinocytes (Chodaczek et al., 2012) and is absent in *Ahr*$^{-/-}$ mice (Kadow et al., 2011; Li et al., 2011), may fulfil protective functions in cutaneous inflammation (Girardi et al., 2006; Kadow et al., 2011). However, mice with selective *Ahr* deficiency resulting from Rag1 Cre-mediated deletion also lack this population, yet did not display the widespread overreaction seen in *Ahr*$^{-/-}$ skin although they displayed some abnormalities in the epidermis. All bone-marrow chimeras lacked epidermal $\gamma\delta$ T cells because the hosts were Rag1 deficient and epidermal $\gamma\delta$ T cells cannot be reconstituted by BM from adult mice, yet only those chimeras with *Ahr* deficiency in the nonhematopoietic compartment displayed the full phenotype seen in *Ahr*-deficient mice.

Both psoriasis pathogenesis in humans and the IMQ model of psoriasiform skin inflammation in the mouse are T cell dependent and rely on the cross-talk between adaptive immune cells and epidermal cells. Thus, it is not surprising that in the complete absence of effector T cells, such as in *Rag1*$^{-/-}$ mice, lack of AhR in keratinocytes did not result in increased disease severity.

IL-23-producing myeloid DCs are critical for the IMQ model, whereas plasmacytoid DCs (pDCs) and the type I interferon pathway are dispensable in this mouse model (Wohn et al., 2013). We did not detect infiltration of pDCs in the skin (data not shown) and *Ahr* deficiency restricted to CD11c-expressing antigen-presenting cells did not recapitulate the hyperinflammation of *Ahr*$^{-/-}$ mice, indicating that these cells, although important for establishing inflammation in the first place, were not drivers for the overreaction seen in *Ahr*$^{-/-}$ mice. Of note is the effect of *Ahr* deletion in different cell types on expression of IL-17 and IL-22. In agreement with previous data (Martin et al., 2009), we found that complete *Ahr* deficiency resulted in higher levels of IL-17 probably due to the predominant infiltration of $\gamma\delta$ T cells, which produce more IL-17 in *Ahr*-deficient mice. This effect, however, was not evident when *Ahr* deficiency was restricted to T and B cells, suggesting that additional interactions with other *Ahr*-deficient cells, e.g., APCs, contribute to IL-17 induction. Furthermore, in contrast to our previous demonstration that in-vitro-differentiated Th17 cells or $\gamma\delta$ T cells require AhR stimulation for IL-22 production, IL-22 was readily detectable in the skin of *Ahr*-deficient mice. It is conceivable that the inflammatory milieu in the skin can provide other factors that could override the requirement for AhR. Finally, the systemic application of the AhR ligand FICZ caused a reduction rather than an increase in IL-17 and IL-22. This is in contrast to the effect of FICZ in vitro or in localized application during EAE, but consistent with its suppressive effect when administered systemically (Duarte et al., 2013).The complexities of cellular interactions in an inflammatory environment that shape these variables require further dissection. However, in the context of our study, we contend that IL-17 and IL-22, although important for the development of psoriasiform inflammation, are not the reason for the exacerbated response of *Ahr*-deficient mice.

The IMQ model of psoriasiform skin inflammation in mice and the pathogenesis process in psoriasis have different kinetics, inflammatory components, and cellular mediators, but nevertheless share critical immunopathological features. Although dispensable for the IMQ model, both type I and II interferon pathways play an important role in human psoriasis (Bowcock et al., 2001). Type I IFN is critical in the early phases of disease initiation in a clinically relevant skin xenotransplant model where it triggers activation and expansion of autoimmune T cells, leading to fully fledged psoriasis plaque formation (Nestle et al., 2005).

A strong IFN-γ genomic (Bowcock et al., 2001) and cellular (Austin et al., 1999) signature is present in psoriasis, and intradermal injection of IFN-γ has been shown to induce several molecular and histological features characteristic of psoriatic lesions in both healthy and psoriatic human skin (Johnson-Huang et al., 2012). *AHR* ligation in human skin biopsies strikingly modulated type I and II interferon pathways, particularly normalizing the proinflammatory signature present in L skin. On the other hand, pharmacological blockade of the *AHR* pathway in ex vivo human skin biopsies and genetic deletion of *Ahr* in the mouse model resulted in an exacerbation of the inflammatory skin signature, whereas activation of the pathway ameliorated both.

Perhaps not surprisingly, human and mouse data did not show mechanistic similarities in terms of genes or pathways affected by AhR. This could be due to the very different ontogeny of the inflammatory skin response, which is provoked by a single agent and short lasting in the mouse, whereas it is multifactorial and chronic in patients. In contrast, the lack of effect of AhR-mediated activation on other relevant psoriasis gene signatures in human samples, such as the antimicrobial response and tissue remodeling, which are affected in the mouse model, could be the result of the short-term culture of the skin biopsies. However, both murine and human keratinocytes lacking AhR were overreactive to proinflammatory stimuli, suggesting shared aspects of pathology, which may diverge later on in the chronic phase of human psoriasis.

It remains to be clarified what the direct targets of AhR are and how these are linked to the inflammatory networks that are affected. The postulated extensive interaction of AhR with other transcription factors, which may be cell type specific, presents a substantial challenge in identifying direct as well as indirect AhR-mediated effects in inflammatory responses. We found dysregulation of the expression of the AP-1 family member *Junb*, which was substantially upregulated in the inflamed skin of *Ahr*-deficient mice and could be readily induced in in vitro keratinocytes stimulated with IL-1β. Moreover, blocking the AP-1 pathway dampened the increase of proinflammatory genes in *Ahr*-deficient keratinocytes. Whereas deletion of *Junb* together with *Jund* in mouse keratinocytes resulted in a skin phenotype resembling psoriasis (Zenz et al., 2005), *JUNB* is upregulated

in psoriatic skin (Haider et al., 2006) and a recent comprehensive meta-analysis of the psoriasis transcriptome has revealed an enrichment of AP-1-binding sites among psoriasis-increased genes and pinpointed the increased expression of *JUNB* in psoriasis skin to keratinocytes (Swindell et al., 2013), suggesting a role for the AP-1 pathway and JunB as a critical checkpoint of epidermal homeostasis.

The emerging pattern from analysis of physiological functions of AhR in immune cell types indicates that it affects radically different gene patterns in different cells types (Kiss et al., 2011; Lee et al., 2012; Li et al., 2011; Nguyen et al., 2010; Qiu et al., 2012; Veldhoen et al., 2008), suggesting that AhR may be a modulator of gene expression with different target genes. In keratinocytes, AhR signaling favors epidermal differentiation, thus promoting skin barrier formation (van den Bogaard et al., 2013) and as shown here AhR signaling exerts an anti-inflammatory effect in keratinocytes. More research will be needed to identify the molecular mechanisms underlying the function of AhR in the control of skin homeostasis during inflammatory responses.

Under physiological conditions, AhR signaling appears to be tightly regulated and endogenous ligands such as FICZ are rapidly metabolized via the activity of CYP enzymes that are downstream targets of AhR activation, most notably CYP1A1, the main extrahepatic cytochrome p450 enzyme under control of the AhR (Wincent et al., 2009). In fact it is now assumed that prolonged AhR signaling in response to TCDD, for example, causes dysregulation of its physiological functions (Bock and Köhle, 2006; Mitchell and Elferink, 2009). In keeping with this, expression of a constitutive active form of AhR in keratinocytes caused skin lesions (Tauchi et al., 2005), and we and others (van den Bogaard et al., 2013) have shown beneficial consequences of physiological AhR activation in skin. Interestingly, kynureninase (*KYNU*), an enzyme of the tryptophan catabolism degrading the putative AhR agonist kynurenine, is one of the consistently upregulated genes in psoriatic skin in our data set and in the literature (Tian et al., 2012), and other genes of the tryptophan pathway have also been found to be differentially regulated in psoriatic skin (Gudjonsson et al., 2010). Increased levels of KYNU enzyme might not only reduce kynurenine levels in psoriastic skin but also deplete tryptophan in the tissue, thus interfering in the formation of other AhR ligands such as FICZ. It is possible that deregulation in tryptophan catabolism observed in psoriatic skin might decrease AhR activation and thus result in increased expression of inflammatory mediators in the skin. Thus, reduced levels of endogenous AhR ligands in psoriatic skin might account for the lack of AhR-mediated control of skin homeostasis. It is important also to consider that dietary ligands, which are of fundamental importance in the AhR-mediated control of intestinal homeostasis, may exert effects in tissues other than the gastrointestinal tract. It is worth mentioning that metabolic syndrome and Crohn's disease are frequently observed comorbidities of psoriasis (Davidovici et al., 2010).

Thus, beneficial effects of AhR activation open the possibility of therapeutic intervention in chronic inflammatory skin disease, but further research is needed to understand the mechanism underlying the physiological consequences of AhR signaling in the immune system.

EXPERIMENTAL PROCEDURES

Details on human skin biopsy culture, animals, RNA isolation, qPCR, RNA sequencing, microarray analysis, and keratinocyte cultures are listed in Supplemental Information.

Human Subjects

Lesional and nonlesional psoriasis skin biopsies were obtained from patients of European descendent recruited at the Psoriasis Center, University Medical Center Schleswig-Holstein, Kiel (Germany) and not receiving any systemic treatment at the time of visit. Four skin biopsies were obtained from discarded healthy skin from donors of European descendent undergoing plastic surgery procedures at Guy's and St. Thomas' Hospital, London (UK). Full patient and healthy control demographics are in Table S1. Our study was conducted in accordance with the Helsinki Declaration, with written informed consent obtained from each volunteer, and approved by the institutional review board of University of Kiel Medical School and Guy's and St. Thomas' Hospital.

Imiquimod Model of Psoriasiform-like Skin Inflammation

Shaved mouse dorsal skin was treated daily for 5 consecutive days with 30 mg Aldara cream containing 5% Imiquimod (IMQ, Meda AB). On day 5, full-thickness skin biopsies of the treated area were collected with a 8 mm biopsy puncher; skin was either snap frozen in liquid N_2 for RNA extraction, fixed in neutral buffered formalin (Sigma) for histopathology analysis, or digested as described below to achieve single-cell suspensions. In some experiments, wild-type C57BL/6 mice received vehicle (olive oil) or 100 μg/kg FICZ (Enzo) intraperitoneally on the day before starting the IMQ treatment and then daily until the day of analysis.

Skin Histopathology

Fixed skin was embedded in paraffin and tissue sections were deparaffinized and stained with H&E for histological analysis. Images were acquired at ×10 magnification with an Olympus VS120 slide scanner. Average epidermal and scale thickness was quantified by a researcher blind to the experimental groups who took five measurements per three sections for each mouse.

Flow Cytometry Analysis of Skin-Infiltrating Cells

Two 8 mm punch biopsies were minced and shook in a digestion cocktail (400 μg/ml Liberase TL [Roche] and 1 mg/ml collagenase D [Roche] in IMDM medium) for 2 hr at 37°C, then mashed through a 70 μl cell strainer. Flow cytometry was performed with antibodies by Biolegend and cells were acquired on a FACSCantoII (BD). For intracellular cytokine staining, cells were stimulated for 4 hr with PdBU (500 ng/ml) and ionomycin (500 ng/ml) in the presence of brefeldin A (1 μg/ml) and Fc block (BD Bioscience), while simultaneously stained for surface markers, then fixed with 3.8% PFA, permeabilized with 0.1% NP-40, and stained for IL-17A and IL-22 (Biolegend).

Conditioned Medium from Skin Cell Suspension of Naive or IMQ-Treated Wild-Type Mice

Conditioned media was obtained from supernatants of skin cell cultures from either naive mouse skin or from skin of wild-type mice treated with IMQ for 2 days: after obtaining single-cell suspensions as described earlier, cells were activated with PdBU and ionomycin for 30 min at 37°C in RPMI medium supplemented with 10% fetal calf serum and 2% Pen-Strep-Gln solution (cRPMI), washed to remove PdBU and ionomycin, and incubated in cRPMI for 3 hr at 37°C; the resulting cell-free supernatant was then used as conditioned media.

Total Cellular Extract and Immunoblot

Primary keratinocytes were washed two times with ice-cold PBS and lysed with NP40 cell lysis buffer (Life Technologies). Protein concentration was determined by the Bio-Rad protein assay kit (Bio-Rad). Cell lysates (20 μg) were separated by SDS-PAGE, transferred onto PVDF membranes (GE Healthcare), and probed with a primary antibody against mouse-JunB (1:1,000, Cell Signaling) and then with anti-rabbit immunoglobulin coupled to peroxidase (1:10,000; GE Healthcare). The immune complexes were visualized by the enhanced chemiluminescence method (Merck Millipore), and results were analyzed by Adobe Photoshop software and normalized to GAPDH.

Statistical Analysis

Statistical analysis was performed with Prism v.5.0 (GraphPad Software). For in vivo experiments, values are expressed as the mean + SEM of *n* animals and data shown are representative of at least two independent experiments. Comparisons were calculated by unpaired t test, if two groups were assessed, or one-way analysis of variance and Bonferroni-corrected p value for multiple comparisons, if more than two groups were assessed. For qPCR validation of human RNA sequencing, fold changes of treatment versus vehicle control-induced gene expression were assessed for normal Gaussian distribution with D'Agostino & Pearson omnibus normality test and then analyzed by paired two-tailed t test or Wilcoxon signed rank test, as appropriate and shown as box and whiskers (Min-Max). The level of statistically significant difference was defined as $p \leq 0.05$.

ACCESSION NUMBERS

The microarray data are available in the Gene Expression Omnibus (GEO) database (http://www.ncbi.nlm.nih.gov/gds) under the accession number GSE47607. The RNA-seq data are available under the accession number GSE47944.

SUPPLEMENTAL INFORMATION

Supplemental Information includes three tables, six figures, and Supplemental Experimental Procedures and can be found with this article online at http://dx.doi.org/10.1016/j.immuni.2014.04.019.

AUTHOR CONTRIBUTIONS

P.D.M. and J.H.D. jointly designed and performed the experiments, analyzed and interpreted the data, and wrote the manuscript.

ACKNOWLEDGMENTS

This work was supported by ERC Advanced Investigator Grant 232782 to B.S. M.J.G. and N.D.L.O. are supported by the Medical Research Council (U117597137). F.O.N, F.V., and I.T. are supported by the National Institute for Health Research (NIHR) Biomedical Research Centre based at Guy's and St Thomas' NHS Foundation Trust and King's College London. The views expressed are those of the authors and not necessarily those of the NHS, the NIHR, or the Department of Health. We would like to thank the Biological Services for breeding and maintenance of our mouse strains. We furthermore acknowledge technical support by the FACS facility, Histology facility, and High Throughput Sequencing Group at the NIMR. We are grateful to psoriatic patients and healthy volunteers for their participation. We thank S.K. Mrowietz for technical assistance, M. Jaskolski and L.-M. Philipp at the Psoriasis Center, University Medical Center Schleswig-Holstein, for help with psoriatic biopsy collection and processing, D. Pennino and H. Sreeneebus at St. John's Institute of Dermatology for provision of human primary keratinocytes and for healthy skin collection, respectively, and J. Krutmann at the Leibniz Research Institute for Environmental Medicine in Düsseldorf (Germany) for provision of AhR-silenced and EV-HaCaT cell lines.

Received: August 2, 2013
Accepted: April 8, 2014
Published: June 5, 2014

REFERENCES

Austin, L.M., Ozawa, M., Kikuchi, T., Walters, I.B., and Krueger, J.G. (1999). The majority of epidermal T cells in Psoriasis vulgaris lesions can produce type 1 cytokines, interferon-gamma, interleukin-2, and tumor necrosis factor-alpha, defining TC1 (cytotoxic T lymphocyte) and TH1 effector populations: a type 1 differentiation bias is also measured in circulating blood T cells in psoriatic patients. J. Invest. Dermatol. *113*, 752–759.

Bata-Csorgo, Z., and Szell, M. (2012). The psoriatic keratinocytes. Expert Rev. Dermatol. *7*, 473–481.

Bjeldanes, L.F., Kim, J.Y., Grose, K.R., Bartholomew, J.C., and Bradfield, C.A. (1991). Aromatic hydrocarbon responsiveness-receptor agonists generated from indole-3-carbinol in vitro and in vivo: comparisons with 2,3,7,8-tetrachlorodibenzo-p-dioxin. Proc. Natl. Acad. Sci. USA *88*, 9543–9547.

Bock, K.W., and Köhle, C. (2006). Ah receptor: dioxin-mediated toxic responses as hints to deregulated physiologic functions. Biochem. Pharmacol. *72*, 393–404.

Bowcock, A.M., Shannon, W., Du, F., Duncan, J., Cao, K., Aftergut, K., Catier, J., Fernandez-Vina, M.A., and Menter, A. (2001). Insights into psoriasis and other inflammatory diseases from large-scale gene expression studies. Hum. Mol. Genet. *10*, 1793–1805.

Chodaczek, G., Papanna, V., Zal, M.A., and Zal, T. (2012). Body-barrier surveillance by epidermal $\gamma\delta$ TCRs. Nat. Immunol. *13*, 272–282.

Davidovici, B.B., Sattar, N., Prinz, J., Puig, L., Emery, P., Barker, J.N., van de Kerkhof, P., Ståhle, M., Nestle, F.O., Girolomoni, G., and Krueger, J.G. (2010). Psoriasis and systemic inflammatory diseases: potential mechanistic links between skin disease and co-morbid conditions. J. Invest. Dermatol. *130*, 1785–1796.

Di Cesare, A., Di Meglio, P., and Nestle, F.O. (2009). The IL-23/Th17 axis in the immunopathogenesis of psoriasis. J. Invest. Dermatol. *129*, 1339–1350.

Di Meglio, P., Perera, G.K., and Nestle, F.O. (2011). The multitasking organ: recent insights into skin immune function. Immunity *35*, 857–869.

Duarte, J.H., Di Meglio, P., Hirota, K., Ahlfors, H., and Stockinger, B. (2013). Differential influences of the aryl hydrocarbon receptor on Th17 mediated responses in vitro and in vivo. PLoS ONE *8*, e79819.

Fritsche, E., Schäfer, C., Calles, C., Bernsmann, T., Bernshausen, T., Wurm, M., Hübenthal, U., Cline, J.E., Hajimiragha, H., Schroeder, P., et al. (2007). Lightening up the UV response by identification of the arylhydrocarbon receptor as a cytoplasmatic target for ultraviolet B radiation. Proc. Natl. Acad. Sci. USA *104*, 8851–8856.

Gillner, M., Bergman, J., Cambillau, C., Fernström, B., and Gustafsson, J.A. (1985). Interactions of indoles with specific binding sites for 2,3,7,8-tetrachlorodibenzo-p-dioxin in rat liver. Mol. Pharmacol. *28*, 357–363.

Girardi, M., Lewis, J.M., Filler, R.B., Hayday, A.C., and Tigelaar, R.E. (2006). Environmentally responsive and reversible regulation of epidermal barrier function by gammadelta T cells. J. Invest. Dermatol. *126*, 808–814.

Gudjonsson, J.E., Johnston, A., Dyson, M., Valdimarsson, H., and Elder, J.T. (2007). Mouse models of psoriasis. J. Invest. Dermatol. *127*, 1292–1308.

Gudjonsson, J.E., Ding, J., Johnston, A., Tejasvi, T., Guzman, A.M., Nair, R.P., Voorhees, J.J., Abecasis, G.R., and Elder, J.T. (2010). Assessment of the psoriatic transcriptome in a large sample: additional regulated genes and comparisons with in vitro models. J. Invest. Dermatol. *130*, 1829–1840.

Hahn, M.E., Karchner, S.I., Evans, B.R., Franks, D.G., Merson, R.R., and Lapseritis, J.M. (2006). Unexpected diversity of aryl hydrocarbon receptors in non-mammalian vertebrates: insights from comparative genomics. J. Exp. Zoolog. A Comp. Exp. Biol. *305*, 693–706.

Haider, A.S., Duculan, J., Whynot, J.A., and Krueger, J.G. (2006). Increased JunB mRNA and protein expression in psoriasis vulgaris lesions. J. Invest. Dermatol. *126*, 912–914.

Johnson-Huang, L.M., Suárez-Fariñas, M., Pierson, K.C., Fuentes-Duculan, J., Cueto, I., Lentini, T., Sullivan-Whalen, M., Gilleaudeau, P., Krueger, J.G., Haider, A.S., and Lowes, M.A. (2012). A single intradermal injection of IFN-γ induces an inflammatory state in both non-lesional psoriatic and healthy skin. J. Invest. Dermatol. *132*, 1177–1187.

Kadow, S., Jux, B., Zahner, S.P., Wingerath, B., Chmill, S., Clausen, B.E., Hengstler, J., and Esser, C. (2011). Aryl hydrocarbon receptor is critical for homeostasis of invariant gammadelta T cells in the murine epidermis. J. Immunol. *187*, 3104–3110.

Karin, M., Liu, Zg., and Zandi, E. (1997). AP-1 function and regulation. Curr. Opin. Cell Biol. *9*, 240–246.

Katiyar, S.K., Matsui, M.S., and Mukhtar, H. (2000). Ultraviolet-B exposure of human skin induces cytochromes P450 1A1 and 1B1. J. Invest. Dermatol. *114*, 328–333.

Kim, S.H., Henry, E.C., Kim, D.K., Kim, Y.H., Shin, K.J., Han, M.S., Lee, T.G., Kang, J.K., Gasiewicz, T.A., Ryu, S.H., and Suh, P.G. (2006). Novel compound 2-methyl-2H-pyrazole-3-carboxylic acid (2-methyl-4-o-tolylazo-phenyl)-amide (CH-223191) prevents 2,3,7,8-TCDD-induced toxicity by antagonizing the aryl hydrocarbon receptor. Mol. Pharmacol. *69*, 1871–1878.

Kiss, E.A., Vonarbourg, C., Kopfmann, S., Hobeika, E., Finke, D., Esser, C., and Diefenbach, A. (2011). Natural aryl hydrocarbon receptor ligands control organogenesis of intestinal lymphoid follicles. Science *334*, 1561–1565.

Lee, J.S., Cella, M., McDonald, K.G., Garlanda, C., Kennedy, G.D., Nukaya, M., Mantovani, A., Kopan, R., Bradfield, C.A., Newberry, R.D., and Colonna, M. (2012). AHR drives the development of gut ILC22 cells and postnatal lymphoid tissues via pathways dependent on and independent of Notch. Nat. Immunol. *13*, 144–151.

Li, Y., Innocentin, S., Withers, D.R., Roberts, N.A., Gallagher, A.R., Grigorieva, E.F., Wilhelm, C., and Veldhoen, M. (2011). Exogenous stimuli maintain intraepithelial lymphocytes via aryl hydrocarbon receptor activation. Cell *147*, 629–640.

Lowes, M.A., Russell, C.B., Martin, D.A., Towne, J.E., and Krueger, J.G. (2013). The IL-23/T17 pathogenic axis in psoriasis is amplified by keratinocyte responses. Trends Immunol. *34*, 174–181.

Martin, B., Hirota, K., Cua, D.J., Stockinger, B., and Veldhoen, M. (2009). Interleukin-17-producing gammadelta T cells selectively expand in response to pathogen products and environmental signals. Immunity *31*, 321–330.

McMillan, B.J., and Bradfield, C.A. (2007). The aryl hydrocarbon receptor sans xenobiotics: endogenous function in genetic model systems. Mol. Pharmacol. *72*, 487–498.

Mitchell, K.A., and Elferink, C.J. (2009). Timing is everything: consequences of transient and sustained AhR activity. Biochem. Pharmacol. *77*, 947–956.

Nestle, F.O., Conrad, C., Tun-Kyi, A., Homey, B., Gombert, M., Boyman, O., Burg, G., Liu, Y.J., and Gilliet, M. (2005). Plasmacytoid predendritic cells initiate psoriasis through interferon-alpha production. J. Exp. Med. *202*, 135–143.

Nestle, F.O., Kaplan, D.H., and Barker, J. (2009). Psoriasis. N. Engl. J. Med. *361*, 496–509.

Nguyen, N.T., Kimura, A., Nakahama, T., Chinen, I., Masuda, K., Nohara, K., Fujii-Kuriyama, Y., and Kishimoto, T. (2010). Aryl hydrocarbon receptor negatively regulates dendritic cell immunogenicity via a kynurenine-dependent mechanism. Proc. Natl. Acad. Sci. USA *107*, 19961–19966.

Pantelyushin, S., Haak, S., Ingold, B., Kulig, P., Heppner, F.L., Navarini, A.A., and Becher, B. (2012). Rorγt+ innate lymphocytes and $\gamma\delta$ T cells initiate psoriasiform plaque formation in mice. J. Clin. Invest. *122*, 2252–2256.

Qiu, J., Heller, J.J., Guo, X., Chen, Z.M., Fish, K., Fu, Y.X., and Zhou, L. (2012). The aryl hydrocarbon receptor regulates gut immunity through modulation of innate lymphoid cells. Immunity *36*, 92–104.

Rannug, A., and Fritsche, E. (2006). The aryl hydrocarbon receptor and light. Biol. Chem. *387*, 1149–1157.

Swindell, W.R., Johnston, A., Carbajal, S., Han, G., Wohn, C., Lu, J., Xing, X., Nair, R.P., Voorhees, J.J., Elder, J.T., et al. (2011). Genome-wide expression profiling of five mouse models identifies similarities and differences with human psoriasis. PLoS ONE *6*, e18266.

Swindell, W.R., Johnston, A., Voorhees, J.J., Elder, J.T., and Gudjonsson, J.E. (2013). Dissecting the psoriasis transcriptome: inflammatory- and cytokine-driven gene expression in lesions from 163 patients. BMC Genomics *14*, 527.

Tagami, H. (1997). Triggering factors. Clin. Dermatol. *15*, 677–685.

Tauchi, M., Hida, A., Negishi, T., Katsuoka, F., Noda, S., Mimura, J., Hosoya, T., Yanaka, A., Aburatani, H., Fujii-Kuriyama, Y., et al. (2005). Constitutive expression of aryl hydrocarbon receptor in keratinocytes causes inflammatory skin lesions. Mol. Cell. Biol. *25*, 9360–9368.

Tian, S., Krueger, J.G., Li, K., Jabbari, A., Brodmerkel, C., Lowes, M.A., and Suárez-Fariñas, M. (2012). Meta-analysis derived (MAD) transcriptome of psoriasis defines the "core" pathogenesis of disease. PLoS ONE *7*, e44274.

Tseng, H.C., Lee, I.T., Lin, C.C., Chi, P.L., Cheng, S.E., Shih, R.H., Hsiao, L.D., and Yang, C.M. (2013). IL-1β promotes corneal epithelial cell migration by increasing MMP-9 expression through NF-κB- and AP-1-dependent pathways. PLoS ONE *8*, e57955.

Tsoi, L.C., Spain, S.L., Knight, J., Ellinghaus, E., Stuart, P.E., Capon, F., Ding, J., Li, Y., Tejasvi, T., Gudjonsson, J.E., et al.; Collaborative Association Study of Psoriasis (CASP); Genetic Analysis of Psoriasis Consortium; Psoriasis Association Genetics Extension; Wellcome Trust Case Control Consortium 2 (2012). Identification of 15 new psoriasis susceptibility loci highlights the role of innate immunity. Nat. Genet. *44*, 1341–1348.

Van Belle, A.B., de Heusch, M., Lemaire, M.M., Hendrickx, E., Warnier, G., Dunussi-Joannopoulos, K., Fouser, L.A., Renauld, J.C., and Dumoutier, L. (2012). IL-22 is required for imiquimod-induced psoriasiform skin inflammation in mice. J. Immunol. *188*, 462–469.

van den Bogaard, E.H., Bergboer, J.G., Vonk-Bergers, M., van Vlijmen-Willems, I.M., Hato, S.V., van der Valk, P.G., Schröder, J.M., Joosten, I., Zeeuwen, P.L., and Schalkwijk, J. (2013). Coal tar induces AHR-dependent skin barrier repair in atopic dermatitis. J. Clin. Invest. *123*, 917–927.

van der Fits, L., Mourits, S., Voerman, J.S., Kant, M., Boon, L., Laman, J.D., Cornelissen, F., Mus, A.M., Florencia, E., Prens, E.P., and Lubberts, E. (2009). Imiquimod-induced psoriasis-like skin inflammation in mice is mediated via the IL-23/IL-17 axis. J. Immunol. *182*, 5836–5845.

Veldhoen, M., Hirota, K., Westendorf, A.M., Buer, J., Dumoutier, L., Renauld, J.C., and Stockinger, B. (2008). The aryl hydrocarbon receptor links TH17-cell-mediated autoimmunity to environmental toxins. Nature *453*, 106–109.

Walter, A., Schäfer, M., Cecconi, V., Matter, C., Urosevic-Maiwald, M., Belloni, B., Schönewolf, N., Dummer, R., Bloch, W., Werner, S., et al. (2013). Aldara activates TLR7-independent immune defence. Nat. Commun. *4*, 1560.

Wang, A., Al-Kuhlani, M., Johnston, S.C., Ojcius, D.M., Chou, J., and Dean, D. (2013). Transcription factor complex AP-1 mediates inflammation initiated by *Chlamydia pneumoniae* infection. Cell. Microbiol. *15*, 779–794.

Wincent, E., Amini, N., Luecke, S., Glatt, H., Bergman, J., Crescenzi, C., Rannug, A., and Rannug, U. (2009). The suggested physiologic aryl hydrocarbon receptor activator and cytochrome P4501 substrate 6-formylindolo[3,2-b] carbazole is present in humans. J. Biol. Chem. *284*, 2690–2696.

Wohn, C., Ober-Blöbaum, J.L., Haak, S., Pantelyushin, S., Cheong, C., Zahner, S.P., Onderwater, S., Kant, M., Weighardt, H., Holzmann, B., et al. (2013). Langerin(neg) conventional dendritic cells produce IL-23 to drive psoriatic plaque formation in mice. Proc. Natl. Acad. Sci. USA *110*, 10723–10728.

Zenz, R., Eferl, R., Kenner, L., Florin, L., Hummerich, L., Mehic, D., Scheuch, H., Angel, P., Tschachler, E., and Wagner, E.F. (2005). Psoriasis-like skin disease and arthritis caused by inducible epidermal deletion of Jun proteins. Nature *437*, 369–375.

Immunity, Volume *40*

Supplemental Information

Activation of the Aryl Hydrocarbon Receptor Dampens the Severity of Inflammatory Skin Conditions

Paola Di Meglio, João H. Duarte, Helena Ahlfors, Nick D.L. Owens, Ying Li, Federica Villanova, Isabella Tosi, Keiji Hirota, Frank O. Nestle, Ulrich Mrowietz, Michael J. Gilchrist, and Brigitta Stockinger

Di Meglio et al.

Supplemental Information

Inventory:

Supplemental Data

Supplemental Table 1, related to Figure 1

Supplemental Table 2, related to Figure 1

Supplemental Table 3, related to Figure 1

Supplemental Figure 1, related to Figure 1

Supplemental Figure 2, related to Figure 2

Supplemental Figure 3, related to Figure 3

Supplemental Figure 4, related to Figure 4

Supplemental Figure 5, related to Figure 6

Supplemental Figure 6, related to Figure 7

Supplemental Experimental Procedures

Supplemental References

Table S1

Sample ID	Age (yrs)	Gender	PASI	Age of Onset	Treatment
**H1**	54	F	NA	NA	NA
**H2**	53	F	NA	NA	NA
**H3**	74	F	NA	NA	NA
**H4**	36	F	NA	NA	NA
**H5**	37	F	NA	NA	NA
**P1**	56	M	4.2	35	topical steroids+Vitamin D
**P2**	52	M	7.9	51	topical steroids+Vitamin D
**P3**	63	F	4.1	53	topical steroids+Vitamin D
**P4**	56	F	2.8	31	topical steroids+Vitamin D
**P5**	30	M	12.5	24	topical steroids
**P6**	56	F	4.2	53	topical Vitamin D
**P7**	60	M	15.6	44	topical steroids+Vitamin D
**P8**	45	M	9.6	38	topical steroids+Vitamin D

Table S1 related to Fig.1: Demographics of healthy donors (H) and psoriasis patients (P). PASI: Psoriasis Area Severity Index; NA: Not applicable

Table S2

Increased expression in Lesional Skin				Decreased expression in Lesional Skin			
**Gene**	**FC: L vs NL**	**p**	**FDR**	**Gene**	**FC: L vs NL**	**p**	**FDR**
S100A9	113.785	2.41E-73	4.37E-69	ZSCAN18	0.273345	4.96E-52	3.15E-48
S100A8	105.068	2.76E-73	4.37E-69	PHF17	0.386304	7.10E-41	2.51E-37
TCN1	192.55	9.32E-71	9.86E-67	IL37	0.0528742	6.59E-38	1.90E-34
TPBG	3.75752	3.61E-56	2.86E-52	TMEM99	0.264457	2.18E-37	5.76E-34
S100A7A	1050.83	2.37E-48	1.25E-44	RP11-54F2.1	0.137792	1.33E-32	2.49E-29
C10orf99	15.8586	7.96E-45	3.61E-41	AQP9	0.127827	1.54E-31	2.57E-28
GJB2	17.0579	5.51E-42	2.19E-38	RORC	0.129265	2.84E-31	4.29E-28
ADAMDEC1	19.2404	1.10E-39	3.50E-36	C1orf95	0.207568	1.96E-29	2.59E-26
KYNU	30.0741	2.19E-36	5.35E-33	F3	0.188385	2.12E-29	2.69E-26
GLYCTK	2.59476	6.06E-36	1.37E-32	C7orf41	0.392176	2.24E-28	2.73E-25
FAM110C	3.86474	9.93E-33	2.05E-29	ZNF134	0.571151	2.92E-28	3.44E-25
CXCR6	8.189	1.03E-32	2.05E-29	KRT77	0.0426745	6.26E-28	7.10E-25
S100A7	62.428	9.70E-32	1.71E-28	CDH20	0.188352	3.31E-27	3.39E-24
S100A12	155.573	1.92E-31	3.04E-28	ANKRD33B	0.123088	6.69E-27	6.44E-24
IRAK2	3.02766	5.24E-31	7.57E-28	HS3ST6	0.099026	3.41E-26	3.00E-23
PLBD1	3.58343	1.10E-29	1.52E-26	GJB4	0.0621355	1.61E-25	1.33E-22
PI3	172.237	9.53E-28	1.04E-24	ACAT1	0.522441	1.64E-25	1.33E-22
TRIM14	2.74193	2.52E-27	2.67E-24	WNK2	0.152976	2.46E-25	1.91E-22
ARNTL2	3.4689	3.63E-27	3.60E-24	RAB3B	0.0944153	3.38E-25	2.49E-22
CCRN4L	3.04026	7.40E-27	6.91E-24	RGMB	0.346188	3.68E-25	2.65E-22
MPZL2	4.98868	8.31E-27	7.53E-24	CLDN1	0.283213	7.86E-25	5.20E-22
PRKCQ	11.3831	8.95E-26	7.68E-23	UGT3A2	0.032518	9.23E-25	5.98E-22
CHFR	1.4794	2.22E-25	1.76E-22	IGFL3	0.308453	1.31E-24	8.31E-22
ABCG4	25.4933	3.16E-25	2.38E-22	SOX5	0.264699	1.44E-24	8.98E-22
TMPRSS11D	81.6717	3.92E-25	2.77E-22	ANKH	0.51391	4.68E-24	2.61E-21

Table S2 related to Fig.1: Top 25 most significantly up- (left) and down-(right) regulated genes in lesional (L) *versus* non lesional (NL) psoriasis skin. FC: Fold Change; FDR: False Discovery Rate

Table S3

Gene	p: L+Ag	p: L+Ant	p: NL+Ag	p: NL+Ant	p: H+Ag	p: H+Ant	FC: L+Ag	FC: L+Ant	FC: NL+Ag	FC: NL+Ant	FC: H+Ag	FC: H+Ant	FC: L vs NL	FC: L vs H	FC: NL vs H	p: L vs NL	FDR: L vs NL
IFIT1	3.48E-03	0.0108741	0.0170926	0.000227536	0.0225637	0.400104	0.271201	4.67802	0.521284	3.94389	0.299311	1.65426	3.98471	4.12642	1.03556	0.010481	0.0483474
RSAD2	0.0125038	0.0475	0.00356989	0.000657162	0.00647929	0.837316	0.311598	3.18077	0.355403	4.62689	0.141145	1.16174	6.63058	6.82605	1.02948	0.00259334	0.0155029
IFIT3	0.00178354	0.008592	0.0334997	0.000150454	0.0161344	0.542856	0.32585	4.28298	0.579456	4.00026	0.261064	1.45715	3.32806	3.5218	1.05821	0.000493625	0.00398706
CMPK2	5.75E-03	0.0307245	3.27E-02	0.000829602	1.85E-02	0.349753	0.416924	2.89875	0.620467	3.15291	0.313114	1.66452	5.70972	4.95197	0.867287	0.000482138	0.0039072
MX2	0.00441559	0.015765	0.000402428	0.000383315	0.0762318	0.38643	0.440633	2.92039	0.571176	2.98869	0.432097	1.57498	2.83934	2.57779	0.907883	1.75E-03	1.13E-02
CXCL11	0.0787357	0.00260271	0.0533645	0.0409604	0.00987624	0.850044	0.538549	4.89391	0.324067	2.91547	0.10063	1.16908	2.85354	4.28917	1.50311	0.0137723	0.0601424
ISG15	0.0526282	0.01692	0.02408	0.000277227	0.00478694	0.305322	0.580267	2.69065	0.621024	3.55862	0.325571	1.61215	5.20431	4.78882	0.920165	0.00015084	0.001471
BATF2	0.0376235	0.106463	0.0416507	0.0184969	0.0235779	0.398252	0.583251	1.68586	0.650493	1.91794	0.417733	1.47454	6.71214	7.88844	1.17525	2.14E-09	8.48E-08
USP18	0.0900872	0.0201236	0.16868	0.00039072	0.0219202	0.351794	0.632513	2.5312	0.789233	2.66018	0.368804	1.56976	2.33296	2.83589	1.21557	0.00191217	0.0120768
EPSTI1	0.0533567	0.0372977	0.200401	0.0106382	0.119217	0.569919	0.654629	1.9546	0.750517	1.96289	0.536965	1.32026	5.14566	4.04371	0.785849	2.85E-07	6.56E-06
MX1	0.155754	0.0245986	0.172298	0.00371374	0.0597084	0.345131	0.655059	2.42122	0.728437	2.31971	0.49316	1.47952	8.45471	7.99345	0.945444	1.23E-06	2.35E-05
HERC5	0.0490263	0.0153939	0.107631	0.00189839	0.0431622	0.412223	0.66046	2.33987	0.767212	2.13846	0.488092	1.43281	1.6389	1.44528	0.88186	0.00560793	0.0291585
SAMD9L	0.0719968	0.0139822	0.169597	0.00327813	0.0738712	0.62234	0.660822	2.45586	0.783635	2.1843	0.543905	1.25636	1.88143	1.85172	0.984211	1.48E-06	2.77E-05
OAS2	0.134484	0.03811	0.171003	0.00644379	0.0339668	0.34688	0.664964	2.07892	0.691782	2.47631	0.437252	1.53101	12.1097	9.52759	0.786774	3.50E-06	5.84E-05
OAS3	0.0859894	0.0415683	0.265737	0.00674979	0.0480101	0.475588	0.726269	1.76326	0.810531	2.00849	0.520991	1.29722	5.58755	6.44058	1.15267	1.22E-15	1.76E-13
OAS1	0.20419	0.0480811	0.358342	0.0175433	0.0375915	0.276218	0.751428	1.8323	0.831491	1.79828	0.539406	1.47208	4.36701	5.42686	1.2427	2.02E-06	3.61E-05
IFI35	0.135327	0.0161809	0.468571	0.00477355	0.00870422	0.346032	0.752037	2.26911	0.895073	1.93329	0.511845	1.36559	1.47068	1.63651	1.11275	0.000363791	0.0030786
SAMHD1	0.145706	0.0490059	6.44E-01	0.00764016	0.156807	0.519317	0.753535	1.76536	0.911706	1.88208	0.609311	1.31541	1.73972	0.858043	0.493209	2.54E-06	4.40E-05
DDX58	0.175383	0.0150247	0.0923704	0.0024866	0.0816268	0.667979	0.757376	2.24734	0.802057	1.84373	0.634436	1.17508	2.1968	2.43703	1.10935	0.000123005	0.00124191
CXCL10	0.487771	0.00328835	0.0135834	0.00924241	0.0021157	0.928375	0.79966	3.52321	0.274128	4.07699	0.0399647	1.08999	4.2683	4.21451	0.987399	3.26E-04	2.80E-03
IFIT5	0.152304	0.0311479	0.125576	0.00158227	0.398726	0.379979	0.810979	1.66286	0.839452	1.79556	0.826243	1.37541	1.7717	1.5422	0.870465	0.000172904	0.00165117
IFIH1	0.240949	0.0207676	0.0581865	0.00576315	0.0889081	0.594458	0.826696	1.60736	0.790982	1.55465	0.635887	1.22203	2.79037	2.7434	0.983168	3.66E-07	8.16E-06
PARP9	0.483088	0.0495434	0.550367	0.0263906	0.283422	0.699644	0.885571	1.58936	0.909073	1.53066	0.774291	1.12674	3.74152	3.46242	0.925403	1.31E-13	1.29E-11
TRIM21	0.421847	0.0361962	0.8446	0.0100575	0.0988905	0.172658	0.898907	1.44382	0.987141	1.31866	0.813844	1.26434	1.746	1.59127	0.911378	1.07E-07	2.76E-06
SP100	0.297059	0.0373078	0.736999	0.0171899	0.978099	0.720029	0.907692	1.28074	0.977784	1.2972	0.996368	1.07463	1.46259	1.58434	1.08325	1.46E-04	1.43E-03
PML	5.14E-01	0.0380827	5.45E-01	0.0357884	0.00216225	0.675957	0.947273	1.1998	0.951043	1.24419	0.730519	1.05116	1.90067	1.96576	1.03424	1.11E-14	1.36E-12
ABCA12	0.871735	0.0415706	0.567731	0.02446	0.8455	0.776657	0.975855	0.781377	1.06889	0.738881	1.02829	0.947053	2.40896	2.22326	0.922914	1.42E-08	4.65E-07
CERS3	0.860371	0.0470667	0.68809	0.0455622	0.760594	0.507273	0.98282	0.838116	1.03718	0.794505	0.965676	0.904386	1.75026	1.80434	1.03089	5.52E-07	1.16E-05
MAP3K9	0.940543	0.00437561	0.697828	0.034043	0.853444	0.959826	0.988056	0.683579	1.04274	0.764078	0.97056	1.00978	1.56707	1.71202	1.09249	1.22E-04	1.23E-03
ITSN2	0.862977	0.0262858	0.718483	0.0345619	0.301638	0.446254	1.01603	0.867357	1.02797	0.843763	1.12197	0.844362	1.8204	2.09055	1.1484	1.49E-13	1.45E-11
PNPT1	0.798705	0.0434721	0.72886	0.0033737	0.388444	0.660447	1.03467	1.50751	0.963264	1.54112	0.822087	1.15757	1.6149	1.62388	1.00556	0.000670717	0.0051315
SDR9C7	0.757351	0.00377752	0.27857	0.00644368	0.61257	0.568835	1.04368	0.673535	1.1808	0.642406	0.938203	0.923249	2.20369	2.07802	0.942974	2.84E-07	6.54E-06
EPN3	0.581481	0.0308122	0.0381057	0.0271062	0.11925	0.931338	1.10236	0.700177	1.29464	0.747489	0.784158	1.01736	1.75086	1.84926	1.0562	6.74E-03	3.38E-02
C17orf109	0.0388934	0.187718	0.00686563	0.000135386	0.353922	0.185742	1.40405	0.832905	1.42698	0.614318	0.827568	0.74017	1.4519	1.4531	1.00083	3.87E-03	2.15E-02
ANO8	0.00537266	0.585833	0.000286363	0.0311761	0.668587	0.458632	1.41476	0.947504	1.45735	0.816553	0.955439	0.880626	0.648481	0.840267	1.29575	0.0099654	0.0463966
CD226	0.0453382	0.0383645	0.00292798	0.507886	0.0918451	0.152844	1.54971	0.652754	2.2563	0.858376	1.53213	0.577957	3.99776	2.4301	0.607865	1.52E-13	1.46E-11
AHRR	4.93E-05	0.000454801	7.83E-05	0.00151583	2.67E-05	0.0202052	1.7016	0.616542	1.63431	0.614336	1.6939	0.790912	0.614884	0.705078	1.14668	5.85E-04	4.59E-03
AC007639.1	0.00094916	0.453371	0.00354623	0.00445649	0.0160581	0.153311	1.71587	0.890429	1.41016	0.742814	1.49826	0.831918	1.62255	1.72446	1.06281	0.000639772	0.0049292
SLC45A4	3.91E-02	0.884789	9.58E-05	2.05E-03	2.14E-01	0.0260426	1.82936	0.970944	1.67703	0.640865	1.19485	0.673148	0.516201	0.60455	1.17115	0.00280904	0.0165431
SECTM1	7.74E-05	0.0741765	0.0105874	0.291886	0.569507	0.106064	1.85991	1.48154	1.40952	0.877685	1.09692	0.754916	1.42806	0.936017	0.655444	0.0421514	0.145896
CA4	0.0198649	0.110305	0.00126367	0.914333	0.00808135	0.761636	1.958	0.567383	2.17619	1.03372	2.73737	1.17579	0.569679	0.840651	1.47566	0.0320201	0.117859

Table S3 related to Fig.1: Psoriasis-relevant genes regulated by ex vivo ligation of AhR in human skin

FC: Fold change; Ag: Agonist; Ant: Antagonist; H: Healthy; L: lesional psoriasis skin; NL: non-lesional psoriasis skin; p: *p* value; FDR: False Discovery Rate

Figure S1

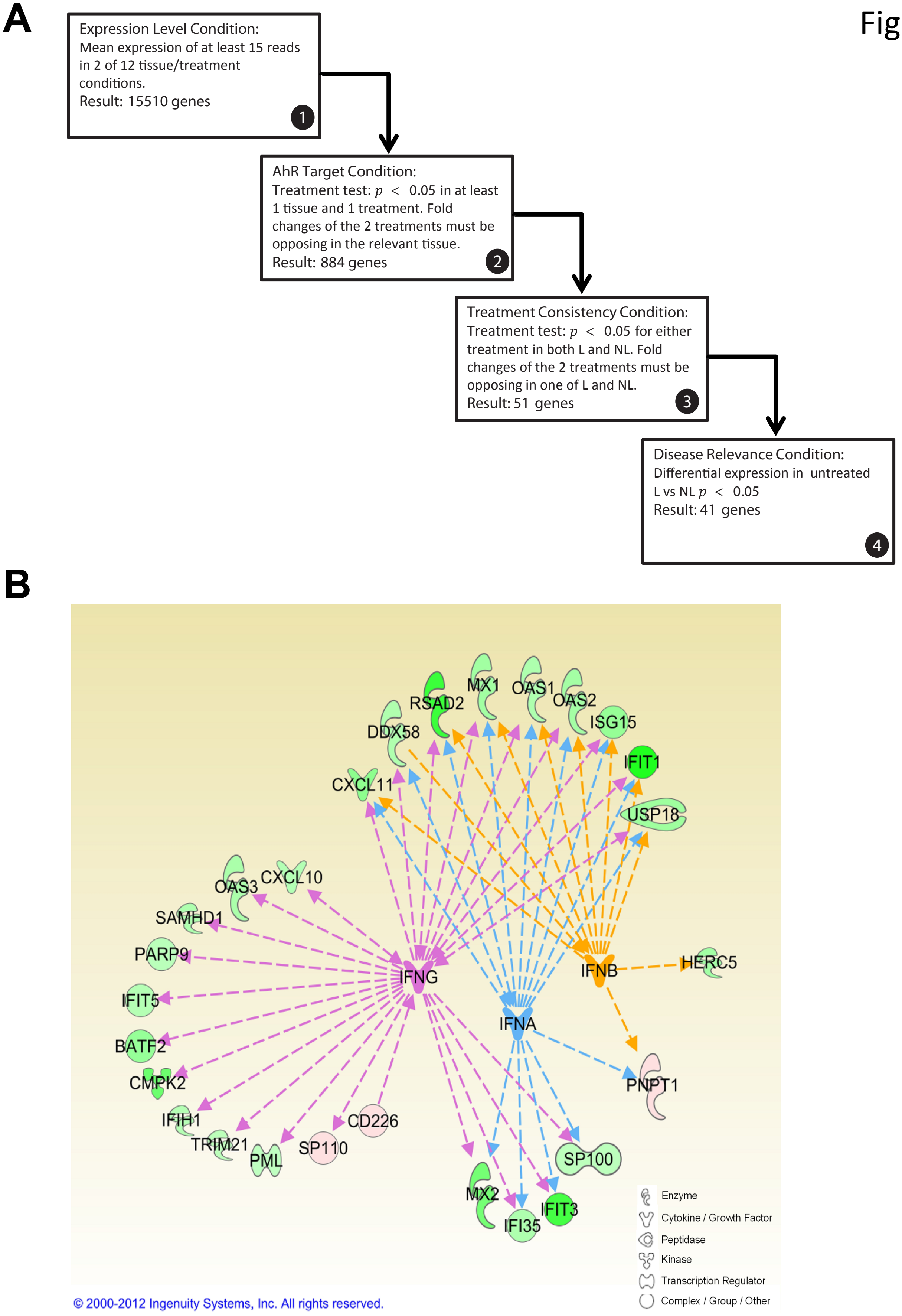

Figure S1 related to Fig.1: (A) Flow chart showing filtering criteria for RNA sequencing gene expressing analysis. (B) Network showing the molecular relationships between gene products modulated by AhR agonist in lesional skin. Genes are represented as nodes and their biological relationship is represented as an edge; the intensity of the node colour indicates the degree of up- (red) or down- (green) regulation

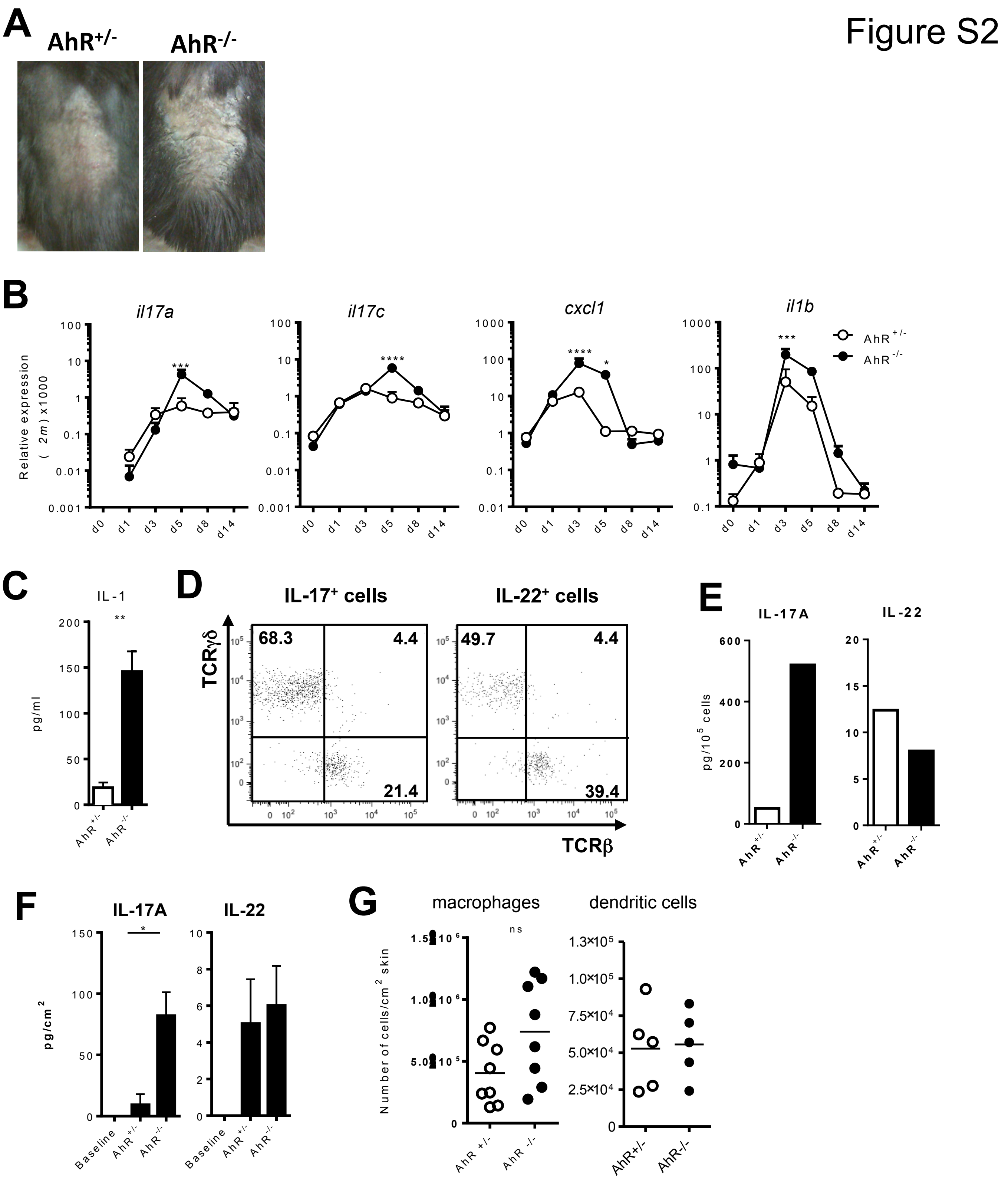

Figure S2 related to Fig.2: (A) Back skin of imiquimod-treated AhR$^{+/-}$ and AhR$^{-/-}$ mice 5 days after treatment initiation. (B) Kinetics of proinflammatory gene expression in whole-skin from imiquimod-treated AhR$^{+/-}$ and AhR$^{-/-}$ mice. (C) IL-1β protein levels from Pdbu/Ionomycin-restimulated skin cell suspension from day 5 imiquimod-treated mice. (D) FACS dot plot showing expression of TCRβ and TCRγδ in IL-17 (left) and IL-22 (right) -expressing cells in the skin of day 5 imiquimod-treated AhR$^{+/-}$ mice. (E) IL-17A and IL-22 protein levels produced by Pdbu/Ionomycin-restimulated FACS-sorted eYFP from day 5 imiquimod-treated IL-17A fate reporter mice. (F) IL-17A and IL-22 protein levels on whole skin lysates from day 5 imiquimod-treated mice. (G) Number of macrophages (CD11b^{+}F4/80^{+}) and dendritic cells (CD11c^{+}) found in imiquimod-treated skin on day 5 after initiating treatment.

Figure S3

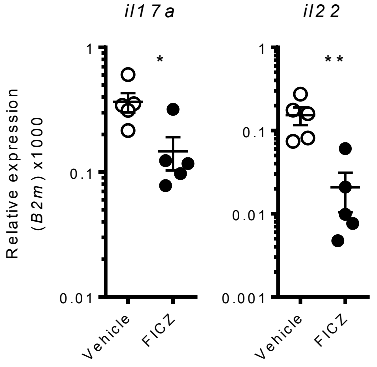

Figure S3 related to Fig.3: mRNA expression of *il17a* and *il22* in skin of imiquimod-treated mice with or without daily FICZ administration on day 5 after initiating treatment.

Figure S4

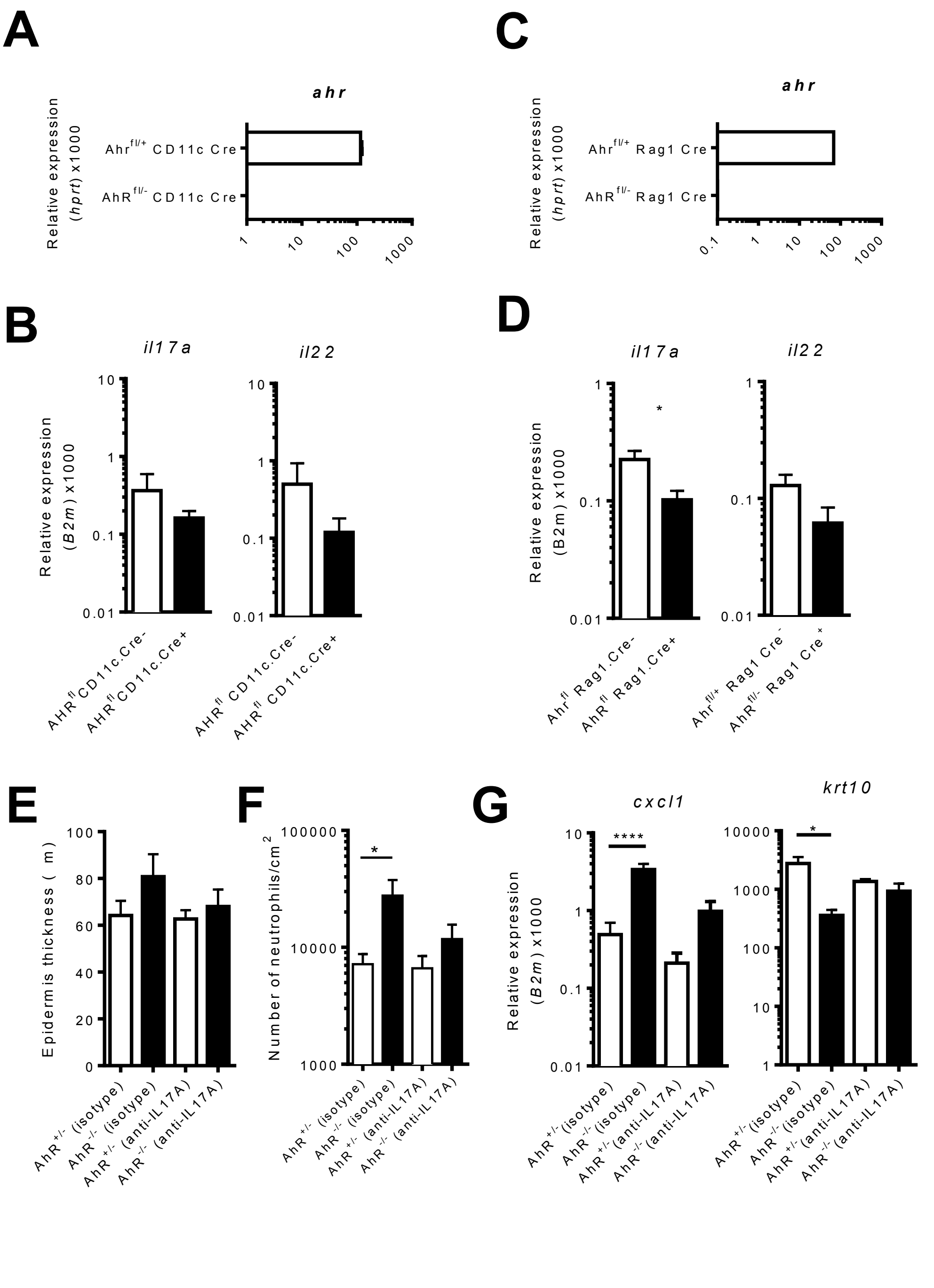

Figure S4 related to Fig.4: (A) qPCR showing deletion of AhR in sorted dendritic cells from AhR^{fl}CD11c.Cre mice. (B) mRNA expression of *il17a* and *il22* in skin of imiquimod-treated AhR^{fl}CD11c.Cre mice on day 5 after initiating treatment. (C) qPCR showing deletion of AhR in sorted T cells from AhR^{fl}CD11c.Cre mice. (D) mRNA expression of *il17a* and *il22* in skin of imiquimod-treated AhR^{fl}Rag1.Cre mice on day 5 after initiating treatment. (E) Quantification of epidermal thickness of imiquimod-treated AhR$^{+/-}$ and AhR$^{-/-}$ mice treated with either isotype or anti-IL-17A antibody,on day 5 after initiating treatment.(F) Number of neutrophils per cm^2 of skin as determined by FACS analysis of Ly6G+ cells in imiquimod-treated AhR$^{+/-}$ and AhR$^{-/-}$ mice treated with either isotype or anti-IL-17A antibody. (G) mRNA expression of cxcl1 and krt10 in skin of imiquimod-treated AhR$^{+/-}$ and AhR$^{-/-}$ mice treated with either isotype or anti-IL-17A antibody.* $p<0.05$.

Figure S5

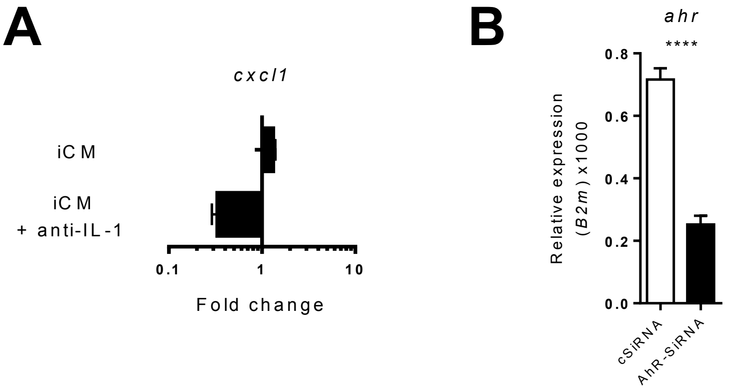

Figure S5 related to Fig.6: (A) *cxcl1* mRNA expression in AhR-deficient keratinocytes stimulated for 24 hours with iCM in the presence or absence of neutralizing anti-IL-1β (10 mg/ml). Data are expressed as fold change over keratinocytes stimulated with in-vitro-reactivated skin cells obtained from naïve wild-type mice. Plots show mean ± SEM. *n* = 2-3 wells per group. (B) *ahr* mRNA expression in human primary keratinocytes, transiently transfected for 48 hours with a non-targeting control SiRNA (cSiRNA, white bars) or in which AhR was transiently silenced (AhR-SiRNA, black bars) and stimulated for further 24 hours with human recombinant IL-1β(10 ng/ml)

Figure S6

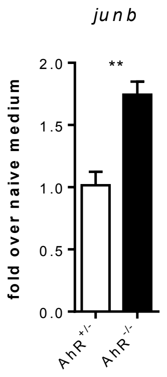

Figure S6 related to Fig.7: (A) *Junb* mRNA expression in $AhR^{+/-}$ (white bars) and $AhR^{-/-}$ (black bars) keratinocytes stimulated for 24 hours with conditioned medium from in-vitro-reactivated skin cells obtained from IMQ-treated wild-type mice. Data expressed as fold change over stimulation with medium conditioned by in-vitro-reactivated skin cells obtained from naïve wild-type mice. Results from one representative experiment of three independent experiments are shown.

Supplemental Experimental Procedures related to Experimental Procedures

Human skin biopsies culture: Psoriasis skin biopsies were quartered and one quarter of each was stored in RNA-later (Life Technologies) at 4°C until further use. The remaining three quarters were cultured with either 0.1% DMSO (vehicle control), the AhR agonist FICZ (250 nM) or the AhR antagonist CH-2233191 (3 μM) in IMDM (Sigma) containing 2% Pen-Strep-Gln solution (Sigma) and 10% knockout Serum replacement factor (Life Technologies) at 37°C in humidified 5% CO_2/95% air for 16h. Individual skin biopsies from healthy donors were processed in the same ways as those of patients.

Animals: Mice were bred in the NIMR animal facility under specified pathogen free conditions. All animal experiments were done according to institutional guidelines and Home Office regulations. C57Bl/6, B6.CD45.1, *Ahr*$^{-/-}$ (Schmidt et al., 1996) (Jackson Stock 002727 B6;129-$^{AhRtm3.1Bra/}$J), *Rag1.Cre*(McCormack et al., 2003), *Cd11c.Cre* (Jackson stock 008068 B6.Cg-Tg($^{Itgax-cre}$)1-1Reiz/J(Caton et al., 2007), *Ahr*$^{fl/fl}$ (Jackson Stock 006203 B6.129(FVB-Ahr$^{tm3.1Bra/J}$), B6.*Rag1*$^{-/-}$and *Ahr*$^{-/-}$*Rag1*$^{-/-}$ mice were used in this study. Mice were bred in the NIMR animal facility under specified pathogen free conditions. All animal experiments were done according to institutional guidelines and Home Office regulations.

Generation of BM chimeras: BM cells were obtained from femurs and tibias of donor mice. Sub-lethally irradiated Rag1 deficient recipient mice were reconstituted by intravenous injection of $1x10^7$ BM cells.

RNA isolation and Quantitative RT-PCR (qRT-PCR): Total RNA was obtained using the Mirvana Kit (Life Technologies) (human skin), or a Polytron PT 3000 tissue homogenizer

(Kinematika) along with TRIzol (Life technologies) (mouse skin) or RNeasy Mini Plus Kit (Qiagen) (*in vitro* cultured KCs), each according to the manufacturer's instructions. mRNA was reverse transcribed into cDNA and gene expression was assessed by quantitative RT-PCR using Taqman assays (Life technologies) according to the manufacturers' instructions. For each sample, mRNA abundance was normalized to the amount of Human acidic ribosomal protein *(HuPO)* or mouse beta 2-microglobulin (*β2m*). Data analysis was performed using either the ΔΔCt method and results are expressed as agonist or antagonist fold change versus vehicle control (human skin samples), or the ΔCt method and results are expressed as mRNA relative expression to *β2m* x1000 in arbitrary units (mouse skin samples), or the ΔΔCt method and results for each genotype are expressed as fold change over respective conditioned medium from naïve mice (*in vitro* keratinocytes).

cDNA Libraries preparation:

cDNA libraries were prepared using the Illumina TruSeqTM RNA sample preparation kit (Low-Throughput protocol) according to manufacturer's instructions. Briefly, 500µg of total RNA sample was used for poly-A mRNA selection using streptavidin-coated magnetic beads. The resulting RNA sample was subjected to thermal mRNA fragmentation using Elute, Prime, Fragment Mix from the kit. cDNA was synthesized from enriched and fragmented RNA using reverse transcriptase Super-Script II (Life technologies) according to manufacturer's instructions. The cDNA was further converted into double stranded DNA using the reagents supplied in the kit. The resulting dsDNA was subjected end-repair, dA-tailing, ligation of platform-specific adaptors, purification reactions and library amplification (15 cycles).

RNA-sequencing (Accession number GSE47944)

Libraries were barcoded and run 4 per lane on an Illumina HiSeq 2000. Sequencing yielded an average of 35 million paired end reads per library. Read pairs were mapped to the Ensembl Human GRCh37 transcriptome using bowtie with the options "-v 3 -a --best -y -I 0 -X 10000". Any read pair mapping only to one or more transcripts of a gene constituted a unique gene count, and any read pair mapping to transcripts of different genes was discarded. On average 60.5% of reads produced unique mappings. Read counts were normalised by the total unique mapping reads per library.

RNA sequencing differential expression analysis

We tested for the influence of AhR agonist and antagonist whilst controlling for any tissue culture effect. We first estimate the perturbation due to culture (if any), then we estimate the fold change and significance of the AhR treatment beyond that of the culture. Let g_{ijkp} be the normalised count of gene i in tissue $j = \{\text{"L, NL, N"}\}$ under treatment k = {U = Untreated, C = DMSO, T=AhR Agonist/Antagonist} in patient p, we assume g_{ijkp} is negative binomially distributed (Cameron and Trived, 1998) with mean μ_{ijk} and variance $\mu_{ijk} + \alpha_{ij}\mu_{ijk}^2$, with α_{ij} the dispersion parameter for gene i in tissue j. We consider the null hypothesis a gene's expression is the result of the mean untreated expression and any culture effects: $\log \mu_{ijk} = \mu_{ij} + x_{Ck}\beta_{C_{ij}}$; and the alternative hypothesis that a gene's expression is due to mean untreated expression, culture and treatment effects: $\log \mu_{ijk} = \mu_{ij} + x_{Ck}\beta_{C_{ij}} + x_{Tk}\beta_{T_{ij}}$. Where, μ_{ij} is the mean untreated expression; $\beta_{C_{ij}}$ and $\beta_{T_{ij}}$ are the log fold changes due to culture and treatment respectively; $x_{Ck} = 1$ when $k = C, T$ and 0 otherwise; $x_{Tk} = 1$ when $k = T$ and 0 otherwise. We compute maximum likelihood estimates of parameters $\alpha_{ij}, \mu_{ij}, \beta_{Cij}$ under the null, and similarly estimates of $\alpha_{ij}, \mu_{ij}, \beta_{Cij}, \beta_{Tij}$ under the alternative hypothesis. We perform a likelihood ratio test: if ℓ_N and ℓ_A are the log-

likelihoods of the null and alternative models respectively then the test statistic $-2(\ell_N - \ell_A)$ is assumed χ_1^2 distributed under the null hypothesis. We apply the test to the 15510 genes that have a mean expression of at least 15 reads in 2 of the 12 tissue/treatment combinations. We select the genes for which we reject the null with $p < 0.05$ in any treatment and tissue, we further require the treatment fold changes must be opposing in the relevant tissue (i.e. β_{Tij} must have different signs for agonist and antagonist). This results in 884 AhR-modulated genes (Fig. 1A). We next filtered for consistency of treatment by requiring one of the two treatments to be significant in both lesional (L) and non-lesional(NL), and that the treatment fold changes must be opposing in one of these two tissues. This results in 51 genes of which we select the 41 most likely to be disease relevant by retaining those differentially expressed in an Untreated L v NL comparison with $p < 0.05$ determined by DESeq (Anders and Huber, 2010).

Microarray gene expression analysis (Accession number GSE47607)

Total RNA was used for the Affymetrix sample preparations (Affymetrix) according to the manufacturer's instructions. Three biological repeats were hybridized to GeneChip Mouse Genome 430 2.0 Arrays. Microarray data were RMA normalized using the R package affy (Bolstad *et al.*, 2003) that is a part of the Bioconductor project (http://www.bioconductor.org). Fold changes were calculated as 2|SLR| if SLR>0 and -2|SLR| if SLR<0, where SLR is the average signal log ratio over the biological replicates. The R package RankProd(Hong *et al.*, 2006) was used to identify regulated genes. Genes having the estimated percentage of false positive predictions pfp<0.01 and fold change FC≥1.5 were considered to be differentially expressed. The gene annotations were obtained from the NetAffx database(Liu *et al.*, 2003). Visualization was performed using Eisen's

Treeview(Eisen *et al.*, 1998). The microarray data were also analyzed for psoriasis associated genes using Ingenuity Pathways Analysis (Ingenuity® Systems, www.ingenuity.com).

Keratinocyte cell culture: For murine primary keratinocyte *in vitro* culture, tails and shaved dorsal mouse skin were processed by mechanical removal of subcutanous fat tissue and floated on 0.5% w/v trypsin (Sigma) for 1 h at 37°C. Epidermal sheets were mechanically separated and suspended in high calcium medium (EMEM medium (Lonza) with 8% calcium-chelated fetal calf serum, 2% Pen-Strep-Gln solution (Sigma) (cEMEM) and 1.3mM $CaCl_2$), submitted to gentle vortexing for 1 min, filtered, and cells seeded in fibronectin- and collagen-coated (BD Biosciences and Life Technologies, respectively) 24-well plates at $3x10^5$ cells/ml in medium-calcium medium (cEMEM supplemented with 0.2mM $CaCl_2$). The following day non-adherent cells were washed away with PBS and cells cultured in low-calcium medium (cEMEM supplemented with 0.05 mM $CaCl_2$). Cells were expanded until reaching 70% confluence and then stimulated with conditioned media (see below) or 10ng/ml IL-1β (R&D Systems), with or without 1 μM Tanshinone IIA (Enzo) for 24 hours.

Human primary keratinocyte were isolated from discarded healthy skin as previously described (Laggner et al., 2011) and cultured in KGM medium (KBM medium, supplemented with KGM-bullet kit, both Lonza) for 2-3 passages. For transient silencing of AhR, cells were seeded in 24-well plates at $35x10^4$ cells/ml and cultured until 65% confluent. Cells were then transfected with 33nM ON-TARGETplus SMARTpool siRNA for human AhR or with ON-TARGETplus Non-targeting siRNA (both Thermo Fisher Scientific Biosciences) using Lipofectamine 2000 (Life Technologies) in KBM medium. Forty-eight hours later, medium was replaced with KGM and cells were stimulated with 10ng/ml human IL-1β for further 24 hours. The human keratinocyte HaCaT cell line, in which AhR had been stable silenced (AhR-silenced HaCaT) or which had been transfected with an empty vector (EV-HaCaT)

(Fritsche et al., 2007), were cultured in DMEM medium supplemented with 10% fetal bovine serum, 2% Pen-Strep-Gln solution, 1 mM sodium pyruvate, 800 μg/ml geneticin (Sigma). Cells were seeded in 24-well plates at $5x10^4$ cells/ml and stimulated the following day with 10ng/ml human IL-1β for 24 hours.

***In vivo* IL-17a blockade in the imiquimod model of psoriasiform-like skin inflammation**: *Ahr*$^{+/-}$ and *Ahr*$^{-/-}$, treated daily for 5 consecutive days with IMQ, received 500 μg/mouse anti-IL-17a antibody or isotype control (both BioXcell) intraperitoneally on the day the IMQ treatment was started and then on day 3 of treatment.

Supplementary References

Anders, S., and Huber, W. (2010). Differential expression analysis for sequence count data. Genome Biol *11*, R106.
Bolstad, B.M., Irizarry, R.A., Astrand, M., and Speed, T.P. (2003). A comparison of normalization methods for high density oligonucleotide array data based on variance and bias. Bioinformatics *19*, 185-193.
Cameron, A.C., and Trived, P.K. (1998). Regression Analysis of Count Data (Cambridge University Press).
Caton, M.L., Smith-Raska, M.R., and Reizis, B. (2007). Notch-RBP-J signaling controls the homeostasis of CD8- dendritic cells in the spleen. The Journal of experimental medicine *204*, 1653-1664.
Eisen, M.B., Spellman, P.T., Brown, P.O., and Botstein, D. (1998). Cluster analysis and display of genome-wide expression patterns. Proc Natl Acad Sci U S A *95*, 14863-14868.
Fritsche, E., Schafer, C., Calles, C., Bernsmann, T., Bernshausen, T., Wurm, M., Hubenthal, U., Cline, J.E., Hajimiragha, H., Schroeder, P.*, et al.* (2007). Lightening up the UV response by identification of the arylhydrocarbon receptor as a cytoplasmatic target for ultraviolet B radiation. Proc Natl Acad Sci U S A *104*, 8851-8856.
Hong, F., Breitling, R., McEntee, C.W., Wittner, B.S., Nemhauser, J.L., and Chory, J. (2006). RankProd: a bioconductor package for detecting differentially expressed genes in meta-analysis. Bioinformatics *22*, 2825-2827.
Laggner, U., Di Meglio, P., Perera, G.K., Hundhausen, C., Lacy, K.E., Ali, N., Smith, C.H., Hayday, A.C., Nickoloff, B.J., and Nestle, F.O. (2011). Identification of a novel proinflammatory human skin-homing Vgamma9Vdelta2 T cell subset with a potential role in psoriasis. J Immunol *187*, 2783-2793.
Liu, G., Loraine, A.E., Shigeta, R., Cline, M., Cheng, J., Valmeekam, V., Sun, S., Kulp, D., and Siani-Rose, M.A. (2003). NetAffx: Affymetrix probesets and annotations. Nucleic Acids Res *31*, 82-86.
McCormack, M.P., Forster, A., Drynan, L., Pannell, R., and Rabbitts, T.H. (2003). The LMO2 T-cell oncogene is activated via chromosomal translocations or retroviral insertion during gene therapy but has no mandatory role in normal T-cell development. Mol Cell Biol *23*, 9003-9013.
Schmidt, J.V., Su, G.H., Reddy, J.K., Simon, M.C., and Bradfield, C.A. (1996). Characterization of a murine Ahr null allele: involvement of the Ah receptor in hepatic growth and development. Proc Natl Acad Sci U S A *93*, 6731-6736.
